# Supplementary material for: Post-translational modifications of Drosophila melanogaster HOX protein, Sex combs reduced
Source: PLoS One. 2020 Jan 13;15(1):e0227642. doi: 10.1371/journal.pone.0227642 (PMC6957346; doi:10.1371/journal.pone.0227642)
Supplement: S3 Fig — The figure shows a region of 41–80 of α-casein (full protein in S2A Fig) and each blue line underneath the primary protein sequence represents a chemically distinct peptide identified by MS/MS analysis. The peptides are heavily modified, and the modifications are indicated by letters or symbols on the blue lines. On the right is the legend for all modifications shown in the figure. Amino acid substitutions have been excluded from the figure. (PDF) [file pone.0227642.s003.pdf]

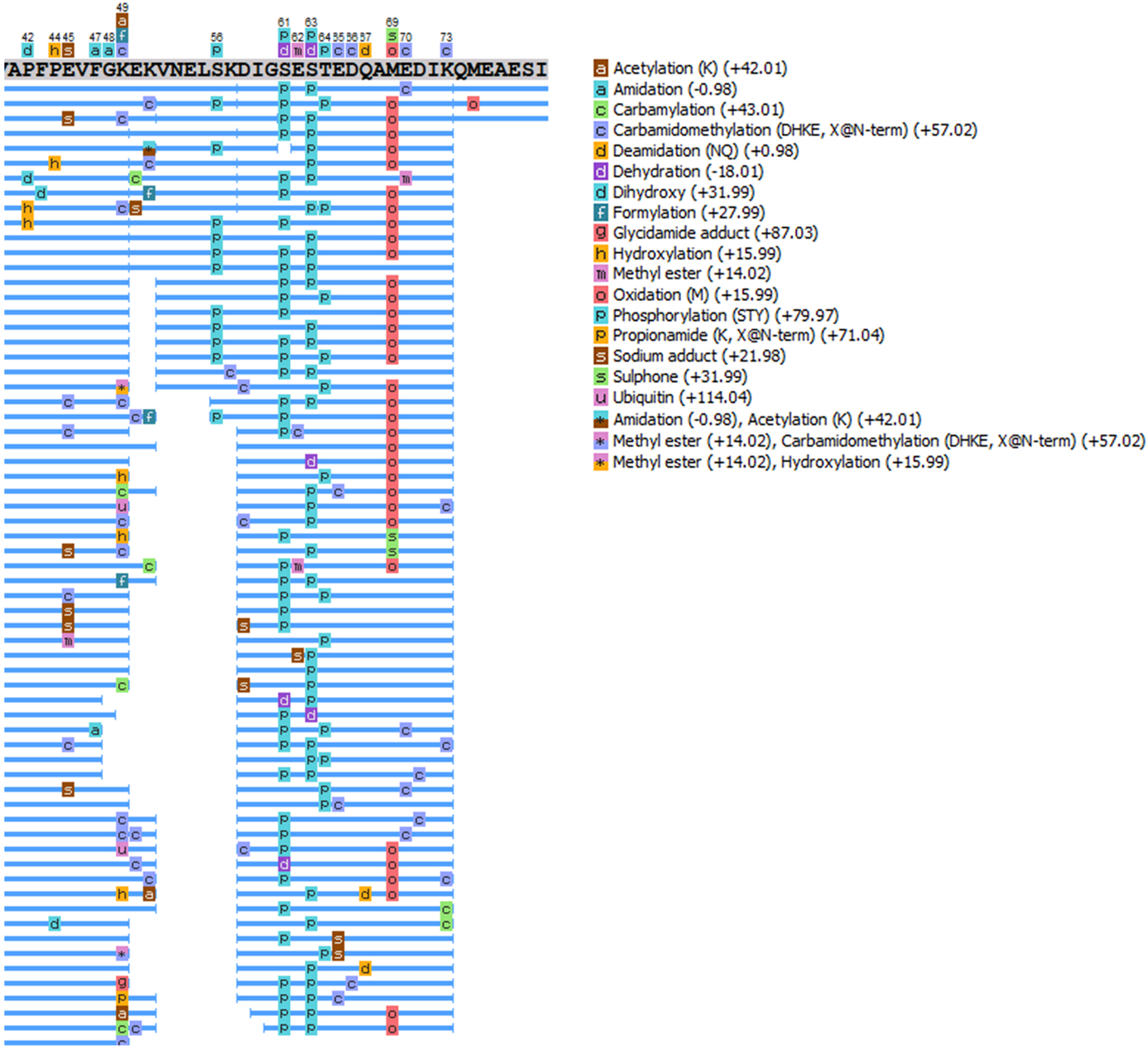

APFPEVFGKEKVNELSKDIGSESTEDQAMEDIKQMEAESI
42 44 45 47 48 49 56 61 62 63 64 65 66 67 69 70 73
a Acetylation (K) (+42.01)
a Amidation (-0.98)
c Carbamylation (+43.01)
c Carbamidomethylation (DHKE, X@N-term) (+57.02)
d Deamidation (NQ) (+0.98)
d Dehydration (-18.01)
d Dihydroxy (+31.99)
f Formylation (+27.99)
g Glycidamide adduct (+87.03)
h Hydroxylation (+15.99)
m Methyl ester (+14.02)
o Oxidation (M) (+15.99)
p Phosphorylation (STY) (+79.97)
p Propionamide (K, X@N-term) (+71.04)
s Sodium adduct (+21.98)
s Sulphone (+31.99)
u Ubiquitin (+114.04)
* Amidation (-0.98), Acetylation (K) (+42.01)
* Methyl ester (+14.02), Carbamidomethylation (DHKE, X@N-term) (+57.02)
* Methyl ester (+14.02), Hydroxylation (+15.99)
