# Supplementary material for: Post-translational modifications of Drosophila melanogaster HOX protein, Sex combs reduced
Source: PLoS One. 2020 Jan 13;15(1):e0227642. doi: 10.1371/journal.pone.0227642 (PMC6957346; doi:10.1371/journal.pone.0227642)
Supplement: S4 Fig — MS2 spectra for the three phosphopeptides identified by LC-MS/MS are shown. (A) Phosphorylation of Serine 185. (B) Phosphorylation of Serine 201. The inset box shows fragment ions with m/z 1690 to 1790. (C) Phosphorylation of Threonine 315. (D) Phosphorylation of Serine 316. (E) Phosphorylation of Threonine 317. The inset box shows fragment ions with m/z 900 to 1400. (F) Phosphorylation of Threonine 324. The peptide sequence and m/z ratio are indicated on the top of the spectra. Positions of fragmentation are shown with vertical lines in the peptide sequence. The box on the right summarizes the evidences of phosphorylation. The relevant fragment ions and their m/z ratios supporting phosphorylation are labelled in the spectra. (PDF) [file pone.0227642.s004.pdf]

**A** MS[2] *m/z* 1118.73

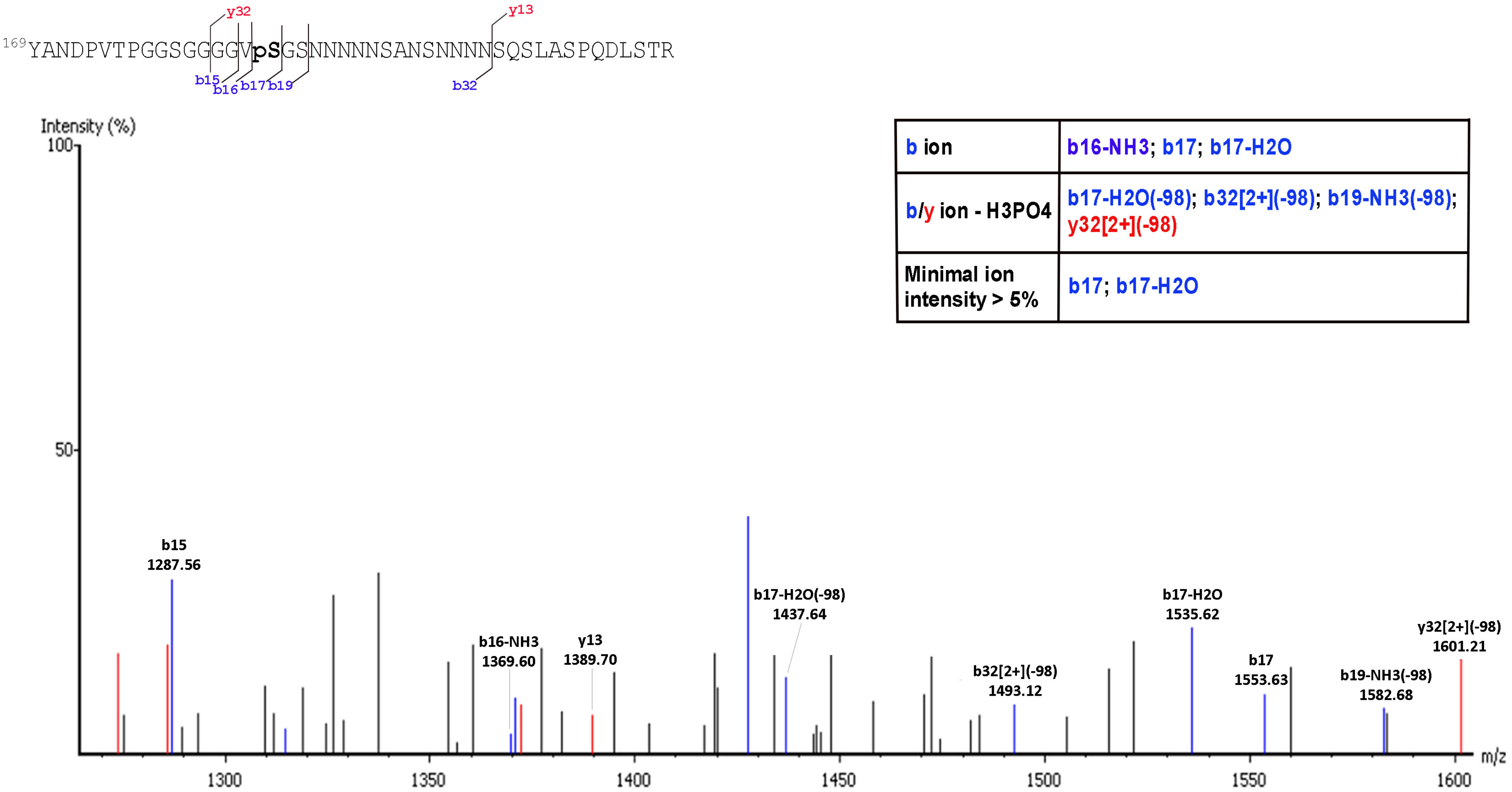


| | |
|---|---|
| b ion | b16-NH3; b17; b17-H2O |
| b/y ion - H3PO4 | b17-H2O(-98); b32[2+](-98); b19-NH3(-98); y32[2+](-98) |
| Minimal ion intensity > 5% | b17; b17-H2O |

## B MS$^2$ *m/z* 1118.98

[169]YANDPVTPGGSGGGGVSGSNNNNNSANSnNNN**pS**QSLASPQDLSTR

(Fragment ions marked on the sequence: y28, y16, y13, y12; b30, b35, b38)

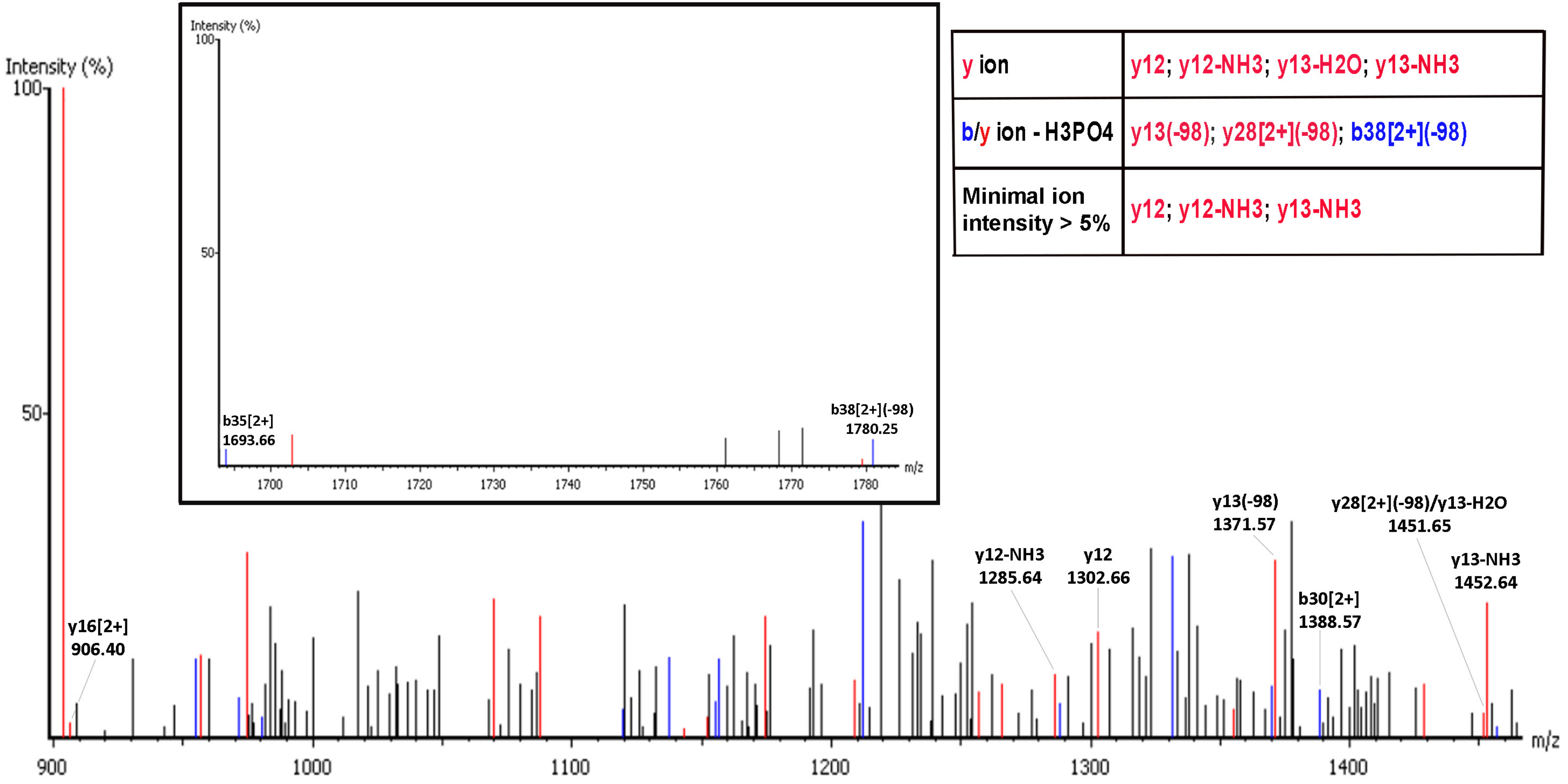


| | |
|---|---|
| y ion | y12; y12-NH3; y13-H2O; y13-NH3 |
| b/y ion - H3PO4 | y13(-98); y28[2+](-98); b38[2+](-98) |
| Minimal ion intensity > 5% | y12; y12-NH3; y13-NH3 |

## C MS$^2$ *m/z* 441.96

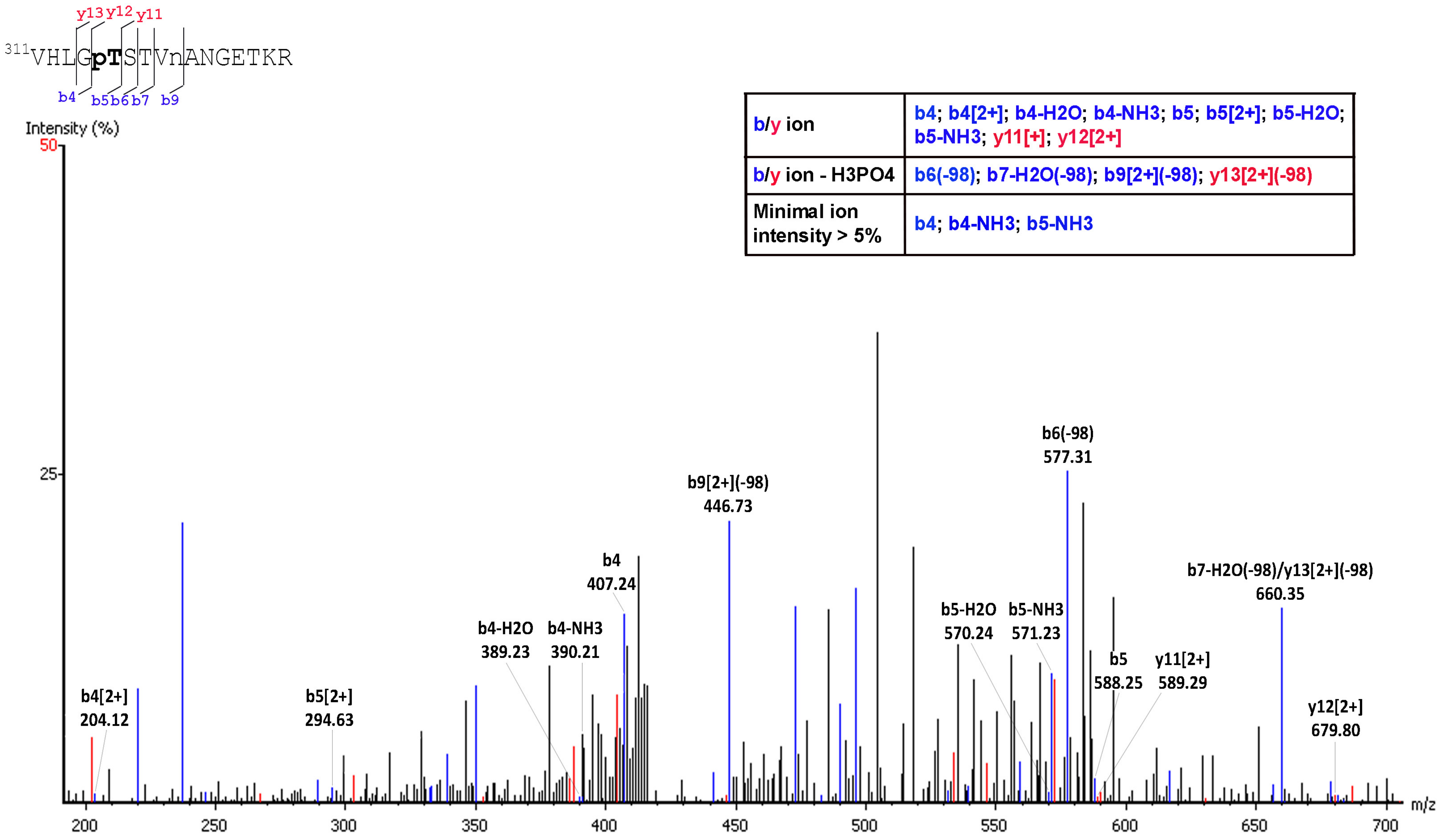


| | |
|---|---|
| b/y ion | b4; b4[2+]; b4-H2O; b4-NH3; b5; b5[2+]; b5-H2O; b5-NH3; y11[+]; y12[2+] |
| b/y ion - H3PO4 | b6(-98); b7-H2O(-98); b9[2+](-98); y13[2+](-98) |
| Minimal ion intensity > 5% | b4; b4-NH3; b5-NH3 |

# D MS$^2$ *m/z* 442.21

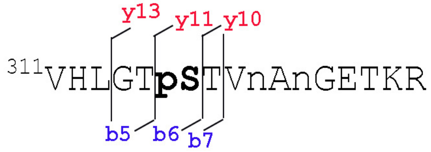


| | |
|---|---|
| b/y ion | b5; b5[2+]; b5-H2O; b5-NH3; b6; b6[2+]; b6-H2O; b6-NH3; y10[2+]; y11[2+] |
| b/y ion - H3PO4 | b6(-98); b7(-98); b7[2+](-98); b7-H2O(-98); y13[2+](-98) |
| Minimal ion intensity > 5% | b5; b5-H2O; b5-NH3; y11[2+] |
| No. of spectra | 2 |

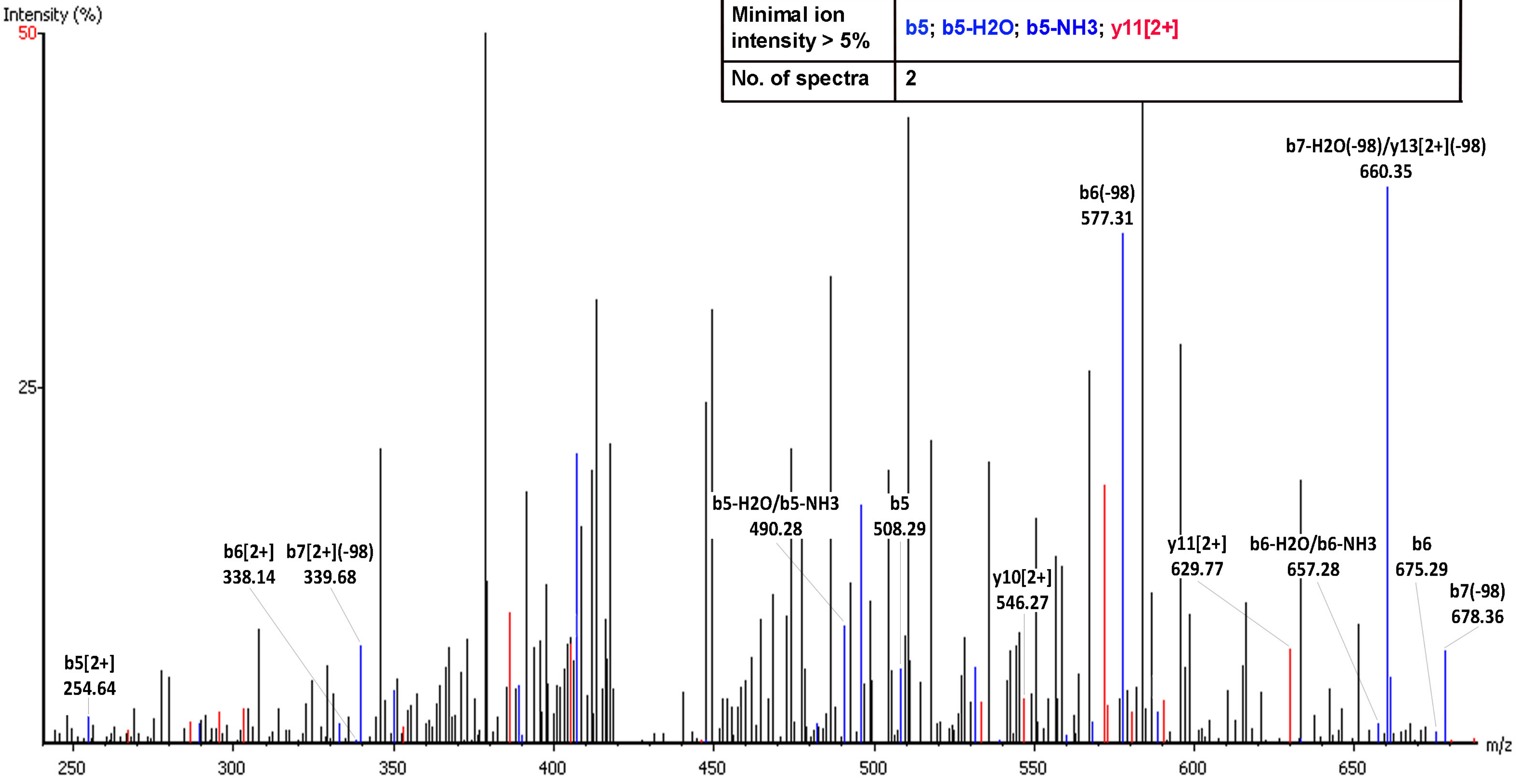

## E MS$^2$ *m/z* 441.96

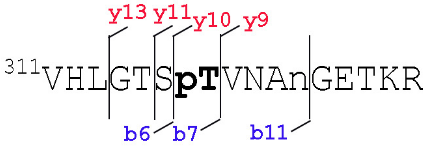


| b/y ion | b6; b6[2+]; b6-H2O; b6-NH3; b7; b7[2+]; b7-NH3; y9[2+]; y10[2+] |
|---|---|
| b/y ion - H3PO4 | b7[2+](-98); b7-H2O(-98); b11-H2O(-98); y13[2+](-98); y13-NH3(-98) |
| Minimal ion intensity > 5% | b6; b6-H2O; b6-NH3; b7[2+]; y9[2+]; y10[2+] |

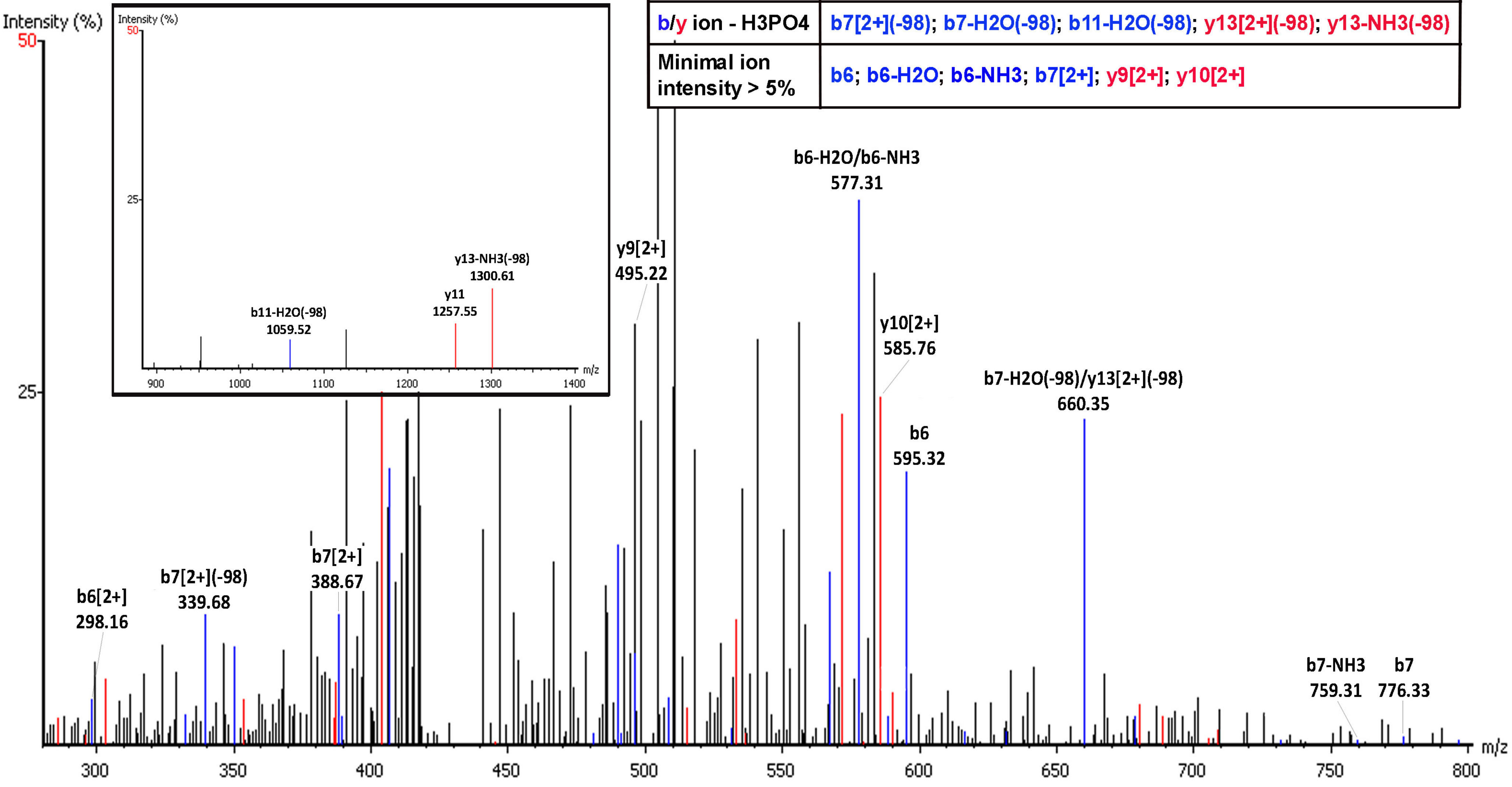

**F** MS$^2$ *m/z* 442.21

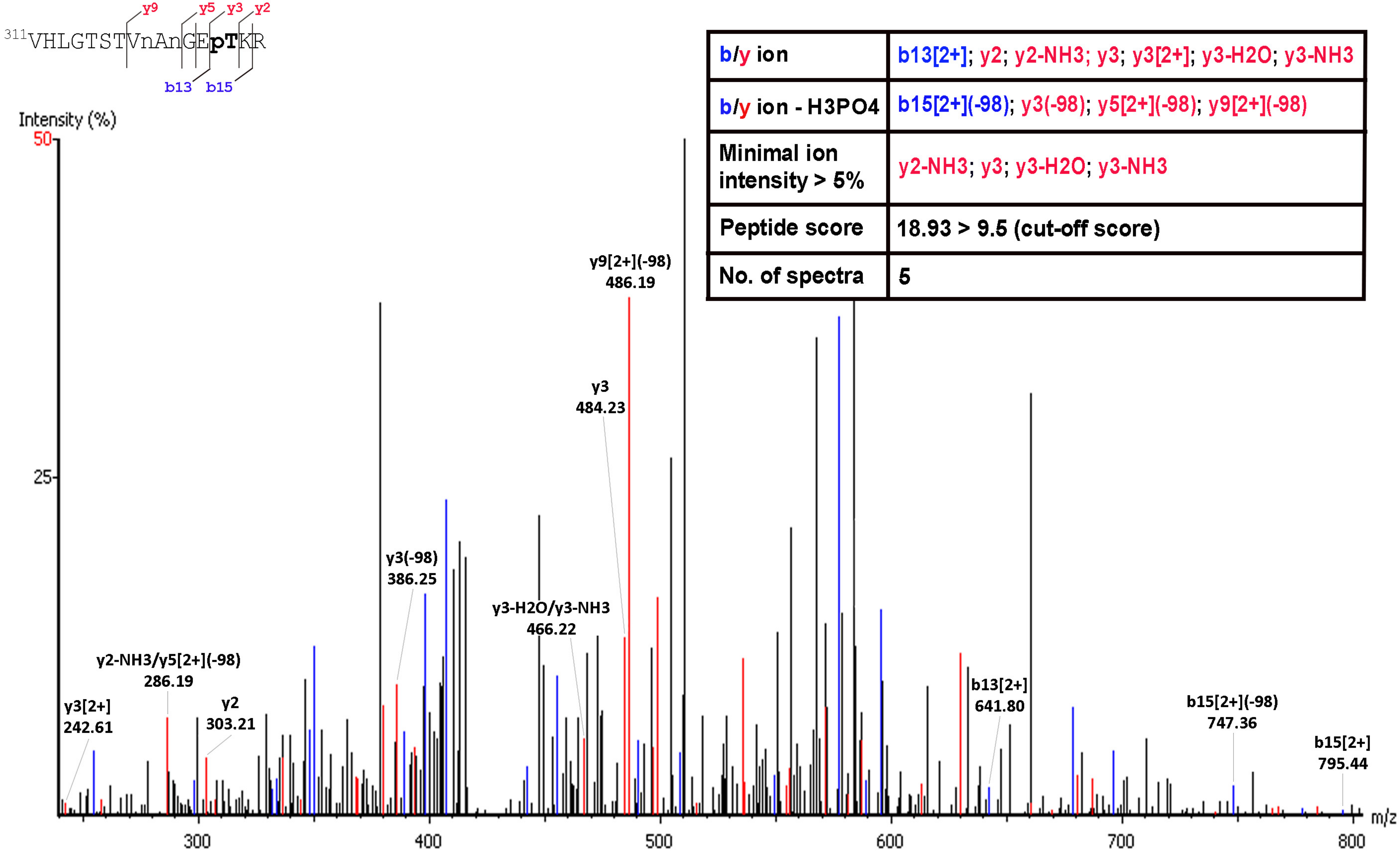


| | |
|---|---|
| b/y ion | b13[2+]; y2; y2-NH3; y3; y3[2+]; y3-H2O; y3-NH3 |
| b/y ion - H3PO4 | b15[2+](-98); y3(-98); y5[2+](-98); y9[2+](-98) |
| Minimal ion intensity > 5% | y2-NH3; y3; y3-H2O; y3-NH3 |
| Peptide score | 18.93 > 9.5 (cut-off score) |
| No. of spectra | 5 |
