# Supplementary material for: Post-translational modifications of Drosophila melanogaster HOX protein, Sex combs reduced
Source: PLoS One. 2020 Jan 13;15(1):e0227642. doi: 10.1371/journal.pone.0227642 (PMC6957346; doi:10.1371/journal.pone.0227642)

A MS^2 *m/z* 906.99

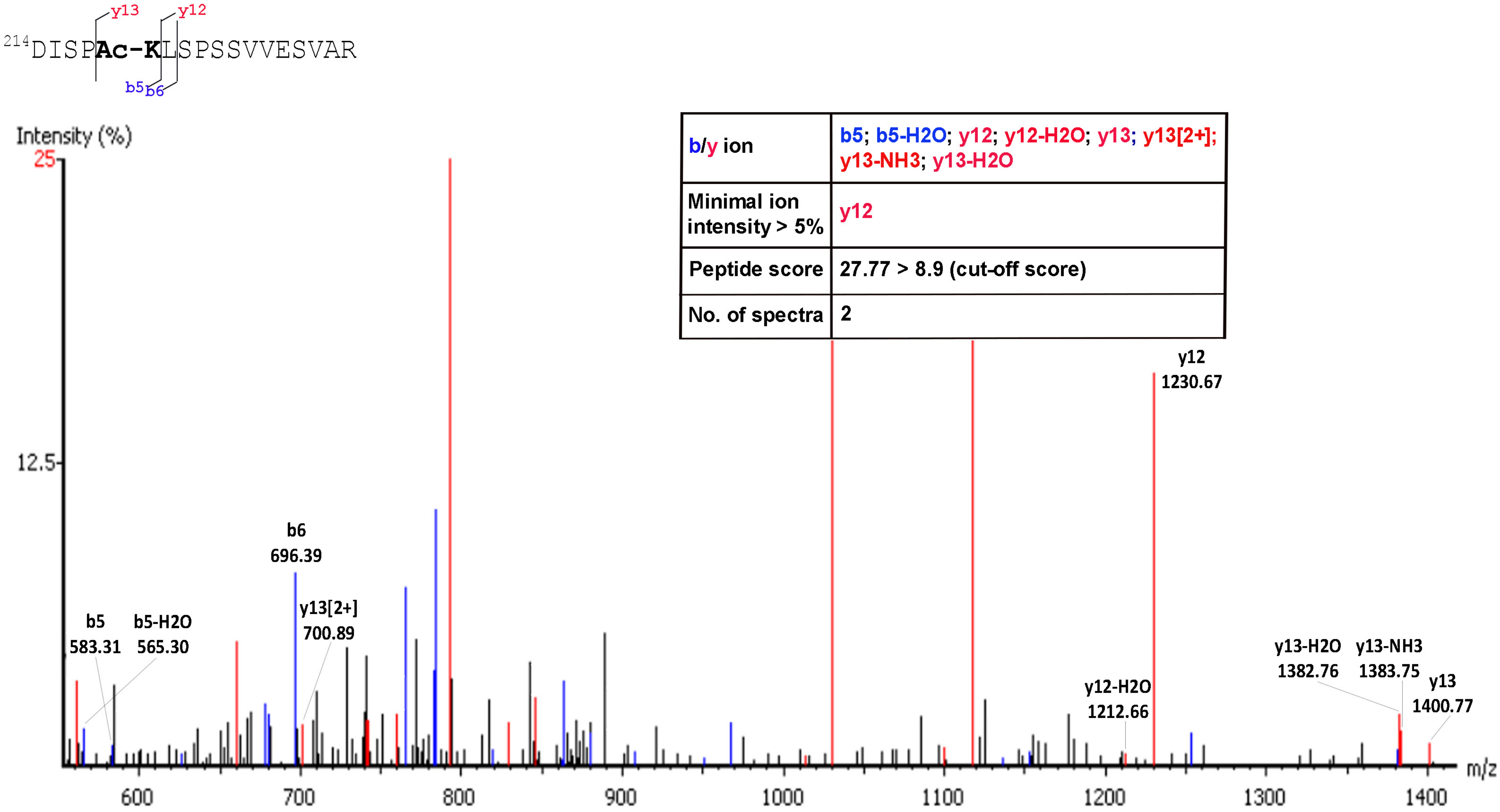

b/y ion	b5; b5-H2O; y12; y12-H2O; y13; y13[2+]; y13-NH3; y13-H2O
Minimal ion intensity > 5%	y12
Peptide score	27.77 > 8.9 (cut-off score)
No. of spectra	2

B MS^2 *m/z* 636.84

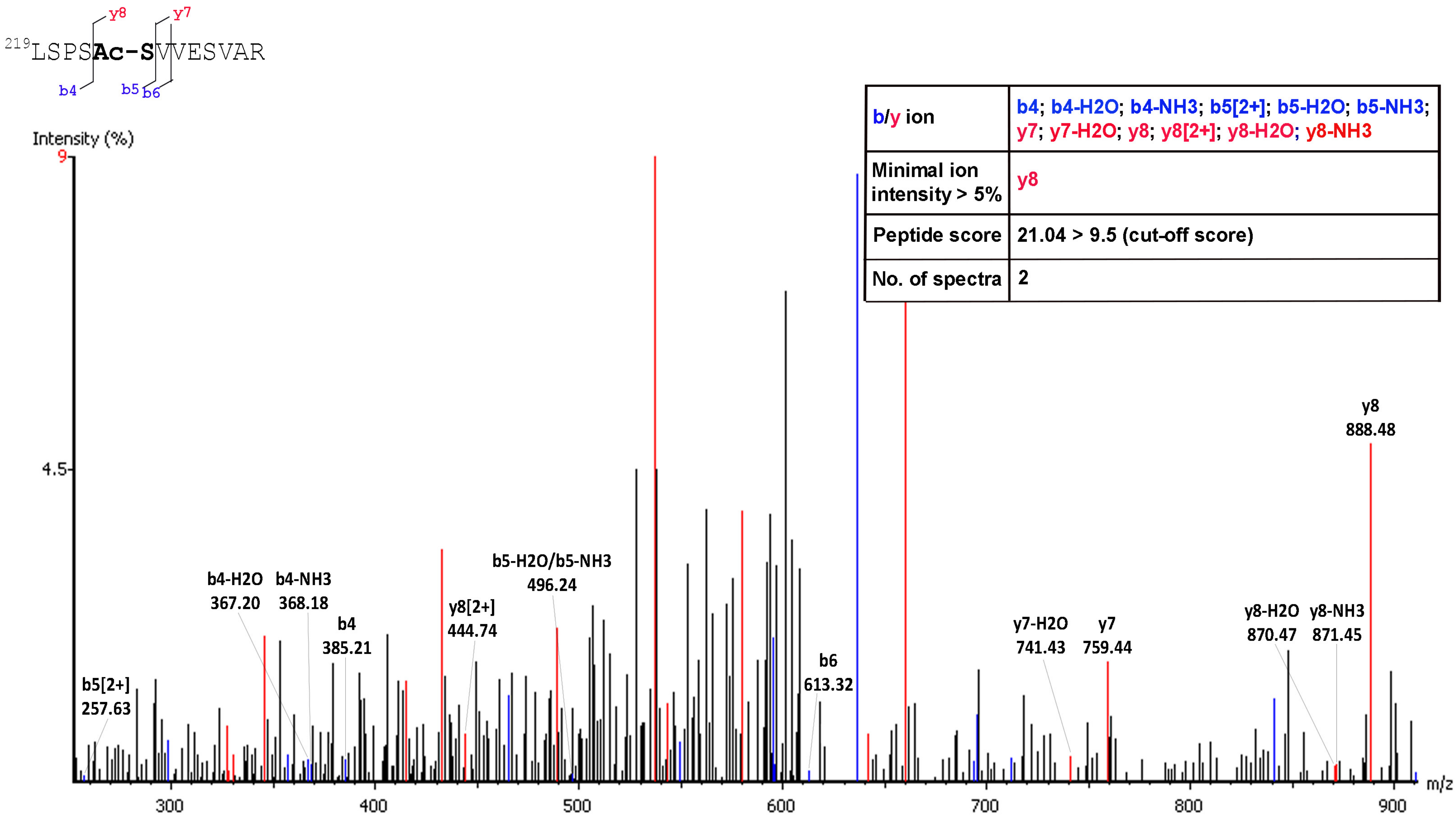

b/y ion	b4; b4-H2O; b4-NH3; b5[2+]; b5-H2O; b5-NH3; y7; y7-H2O; y8; y8[2+]; y8-H2O; y8-NH3
Minimal ion intensity > 5%	y8
Peptide score	21.04 > 9.5 (cut-off score)
No. of spectra	2

C MS^2 *m/z* 680.36

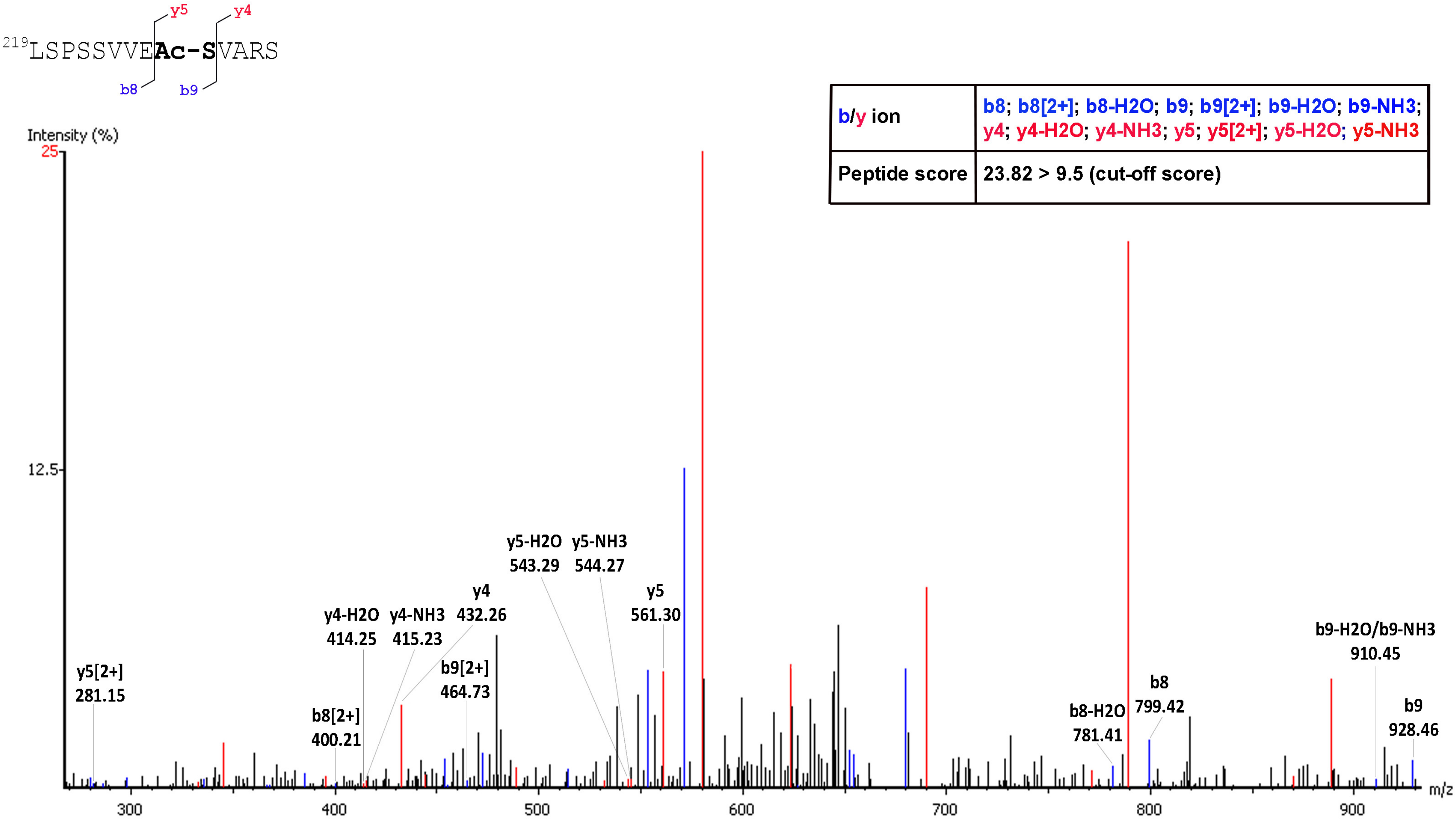

b/y ion	b8; b8[2+]; b8-H2O; b9; b9[2+]; b9-H2O; b9-NH3; y4; y4-H2O; y4-NH3; y5; y5[2+]; y5-H2O; y5-NH3
Peptide score	23.82 > 9.5 (cut-off score)

D MS^2 m/z 758.38

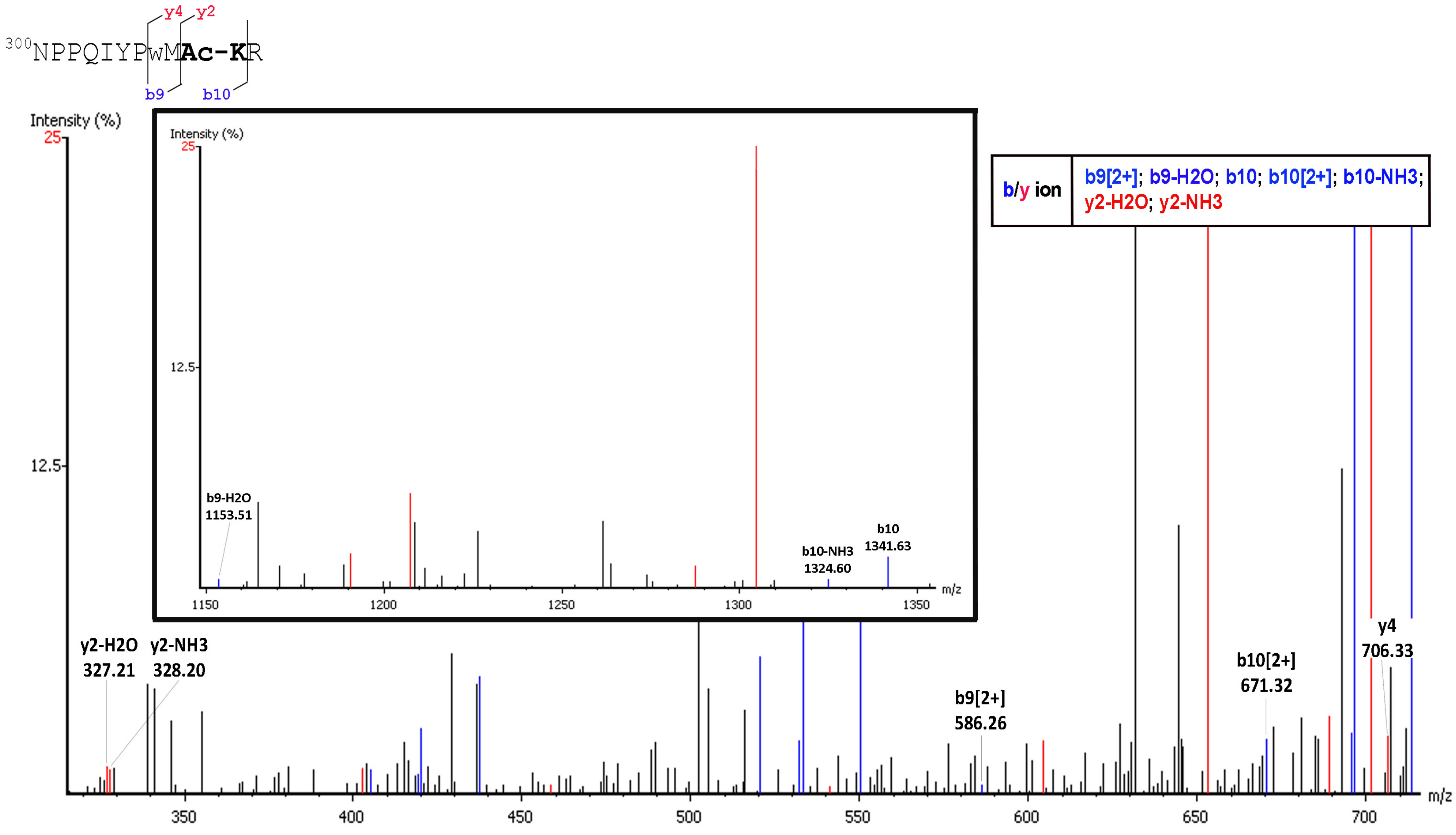

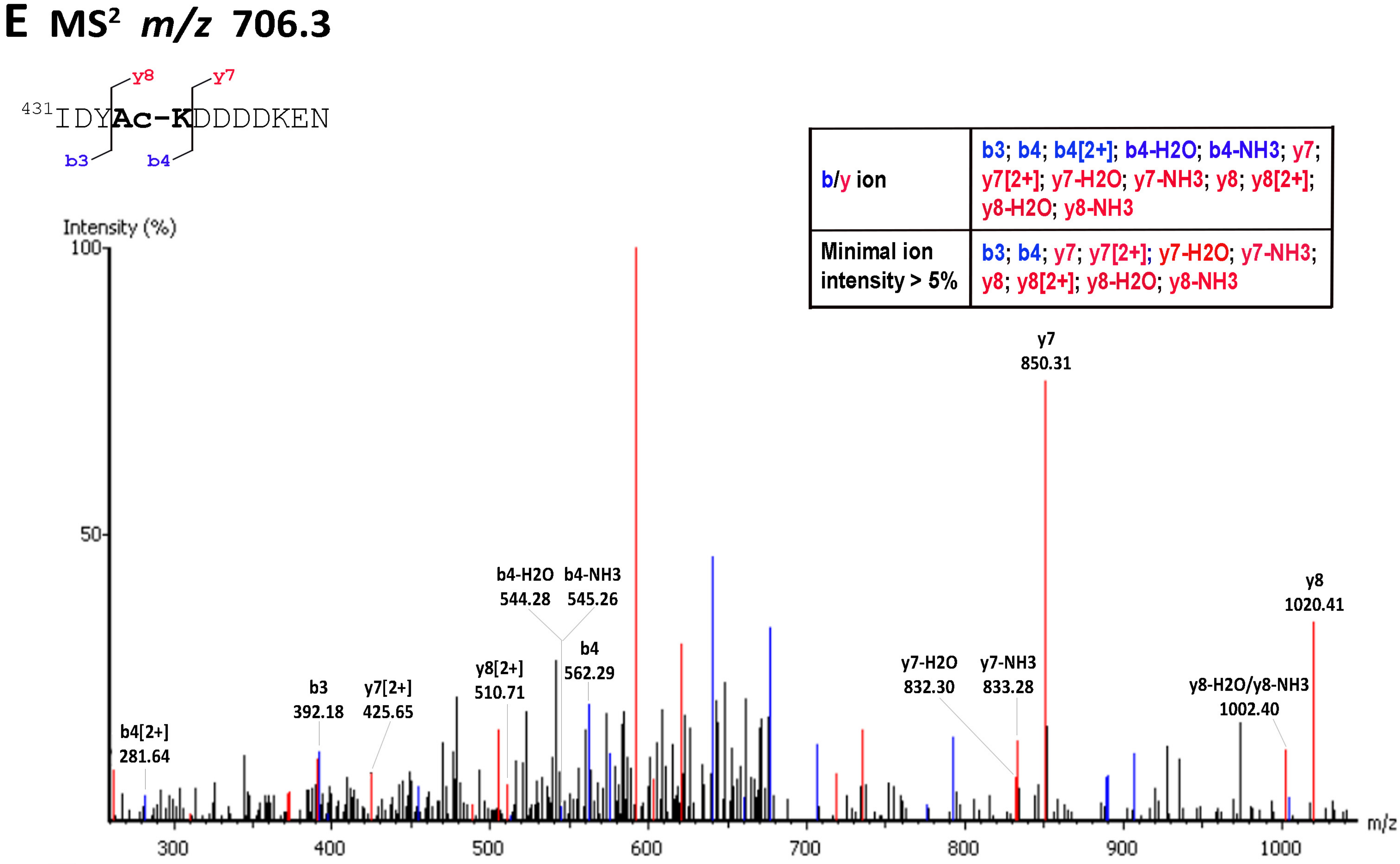
E MS2 m/z 706.3
431IDYAc-KDDDDKEN
y8
y7
b3
b4
b/y ion
b3; b4; b4[2+]; b4-H2O; b4-NH3; y7; y7[2+]; y7-H2O; y7-NH3; y8; y8[2+]; y8-H2O; y8-NH3
Minimal ion intensity > 5%
b3; b4; y7; y7[2+]; y7-H2O; y7-NH3; y8; y8[2+]; y8-H2O; y8-NH3
Intensity (%)
100
50
y7 850.31
y8 1020.41
b4-H2O 544.28
b4-NH3 545.26
b4 562.29
y8[2+] 510.71
b3 392.18
y7[2+] 425.65
b4[2+] 281.64
y7-H2O 832.30
y7-NH3 833.28
y8-H2O/y8-NH3 1002.40
300
400
500
600
700
800
900
1000
m/z

F MS^2 *m/z* 699.29

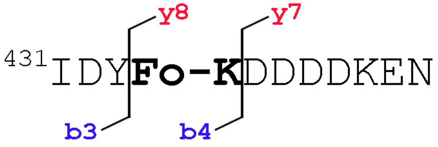

b/y ion	b3; b3[2+]; b3-H2O; b4; b4-H2O; b4-NH3; y7; y7[2+]; y7-H2O; y7-NH3; y8; y8[2+]; y8-H2O; y8-NH3
Minimal ion intensity > 5%	b3; b3-H2O; b4; b4-H2O; y7; y7-H2O; y7-NH3; y8; y8[2+]; y8-NH3
No. of spectra	2

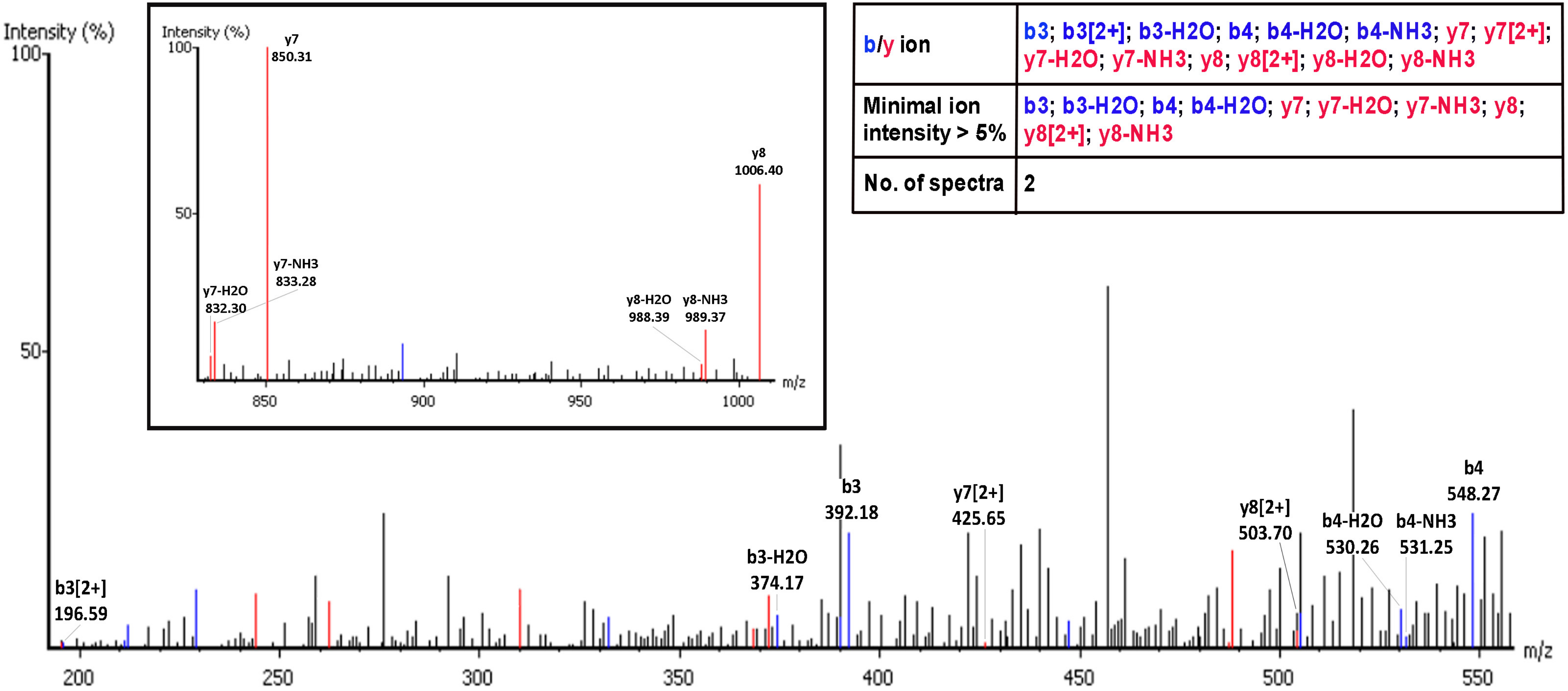

Supplement: S5 Fig — MS2 spectra of the peptide identified by LC-MS/MS is shown. (A) Acetylation of Lysine 218. (B) Acetylation of Serine 223. (C) Acetylation of Serine 227. (D) Acetylation of Lysine 309. The inset box shows fragment ions with m/z 1150 to 1350. (E) Acetylation of Lysine 434. (F) Acetylation of Lysine 439. The inset box shows fragment ions with m/z 390 to 580. The peptide sequence and m/z ratio are indicated at the top of the spectra. Positions of fragmentation are shown with vertical lines in the peptide sequence. The box on the right summarizes the evidence confirming acetylation. The relevant fragment ions and their m/z ratios supporting acetylation are labelled in the spectra. (PDF) [file pone.0227642.s005.pdf]
