# Supplementary material for: Post-translational modifications of Drosophila melanogaster HOX protein, Sex combs reduced
Source: PLoS One. 2020 Jan 13;15(1):e0227642. doi: 10.1371/journal.pone.0227642 (PMC6957346; doi:10.1371/journal.pone.0227642)
Supplement: S6 Fig — MS2 spectra of the peptide identified by LC-MS/MS is shown. (A) Formylation of Lysine 218. The inset box shows fragment ions with m/z 300 to 700. (B) Formylation of Lysine 309. The inset box shows fragment ions with m/z 1100 to 1300. (C) Formylation of Lysine 325. The inset box shows fragment ions with m/z 700 to 1400. (D) Formylation of Lysine 341. (E) Formylation of Lysine 369. The inset box shows fragment ions with m/z 700 to 1100. (F) Formylation of Lysine 434. The inset box shows fragment ions with m/z 830 to 1010. (G) Formylation of Lysine 439. The inset box shows fragment ions with m/z 950 to 1200. The peptide sequence and m/z ratio are indicated at the top of the spectra. Positions of fragmentation are shown with vertical lines in the peptide sequence. The box on the right summarizes the evidence confirming formylation. The relevant fragment ions and their m/z ratios supporting formylation are labelled in the spectra. (PDF) [file pone.0227642.s006.pdf]

A MS² *m/z* 899.98

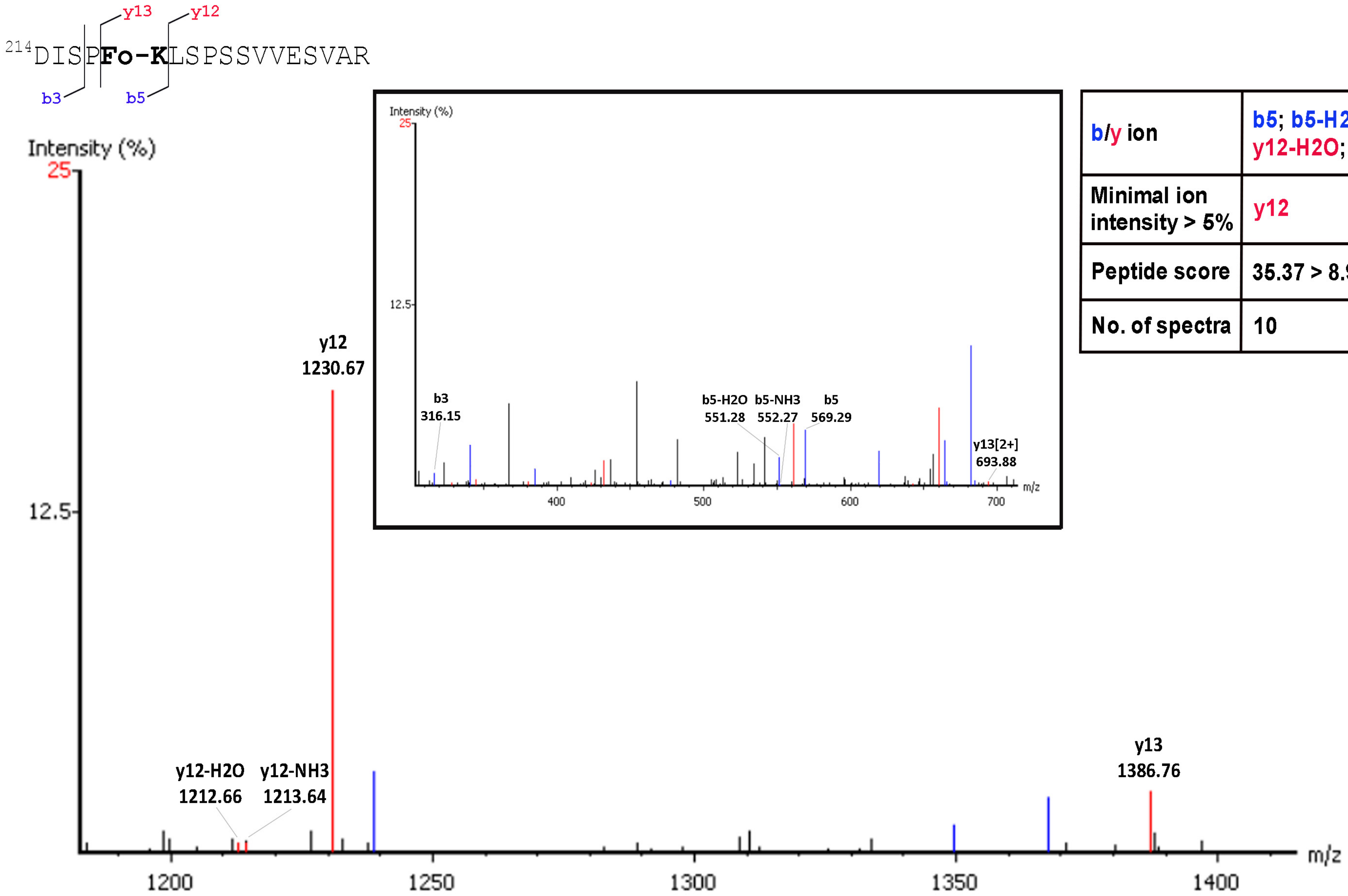

b/y ion	b5; b5-H2O; b5-NH3; y12; y12-H2O; y12-NH3; y13; y13[2+]
Minimal ion intensity > 5%	y12
Peptide score	35.37 > 8.9 (cut-off score)
No. of spectra	10

B MS^2 *m/z* 737.37

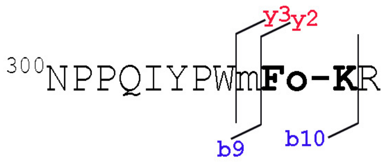

b/y ion	b9; b9[2+]; b9-H20; b10; b10[2+]; b10-NH3; y2; y2-H2O; y2-NH3
Peptide score	21.42 > 8.9 (cut-off score)
No. of spectra	2

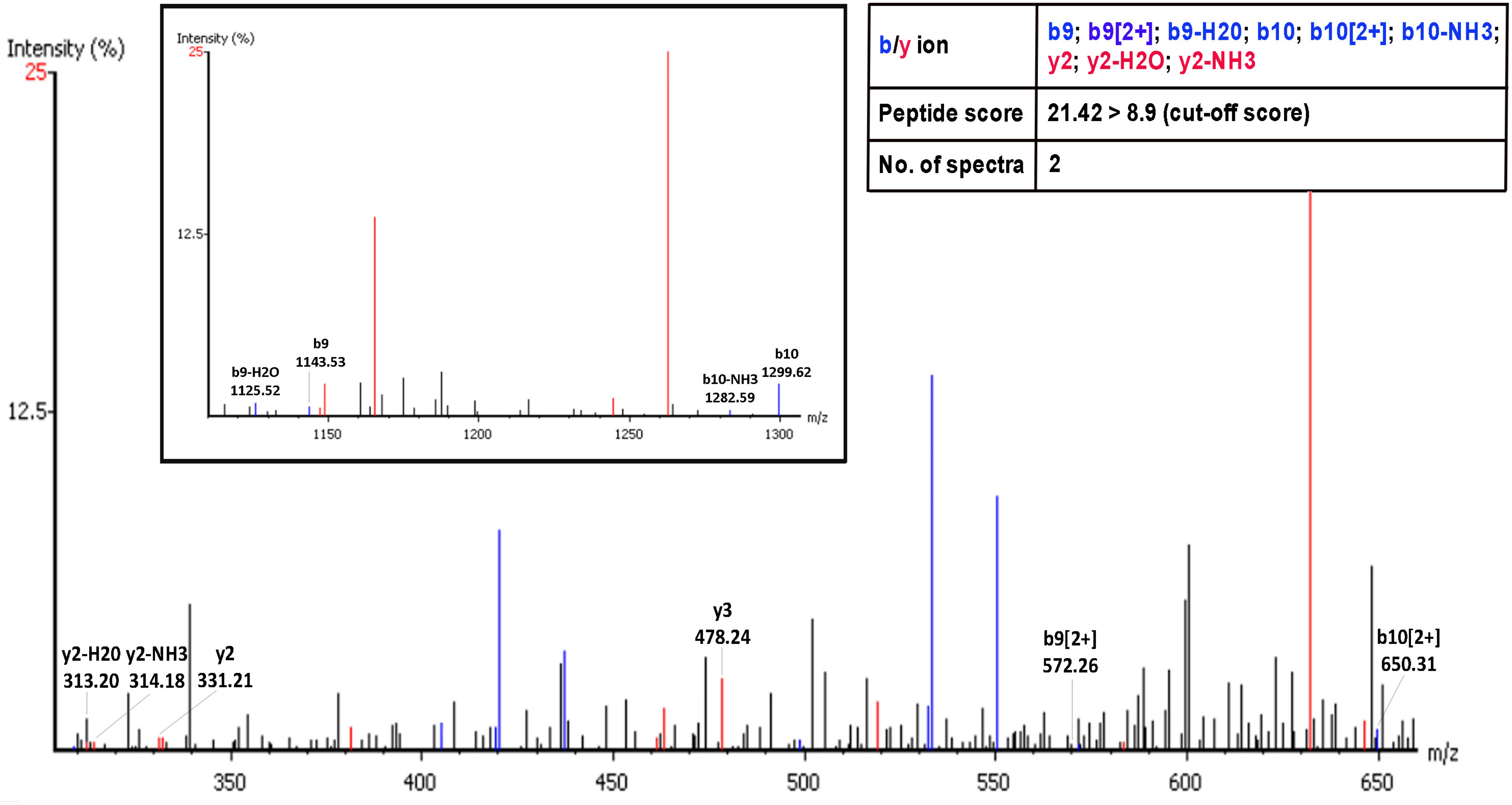

C MS^2 *m/z* 571.3

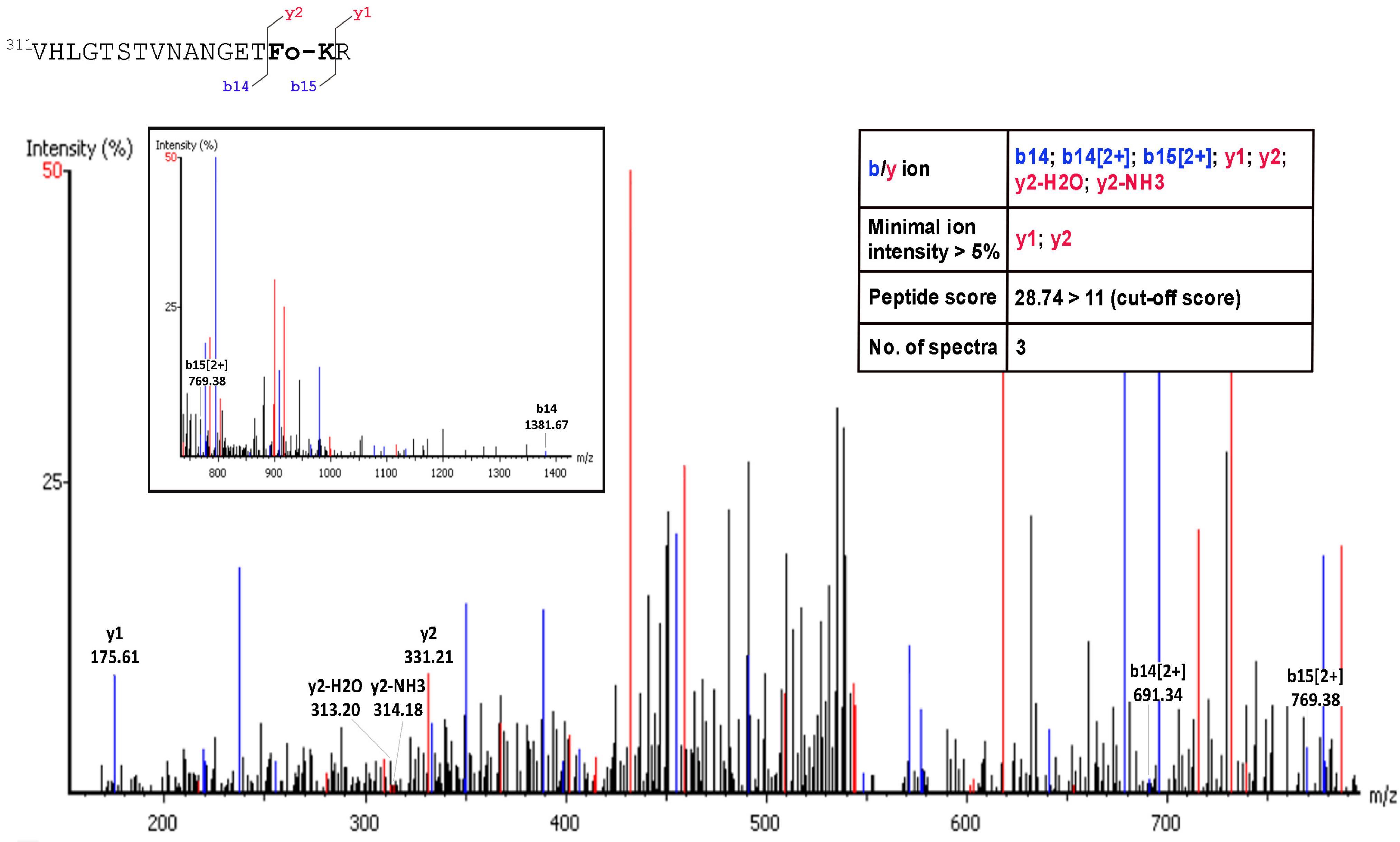

b/y ion	b14; b14[2+]; b15[2+]; y1; y2; y2-H2O; y2-NH3
Minimal ion intensity > 5%	y1; y2
Peptide score	28.74 > 11 (cut-off score)
No. of spectra	3

D MS^2 *m/z* 627.98

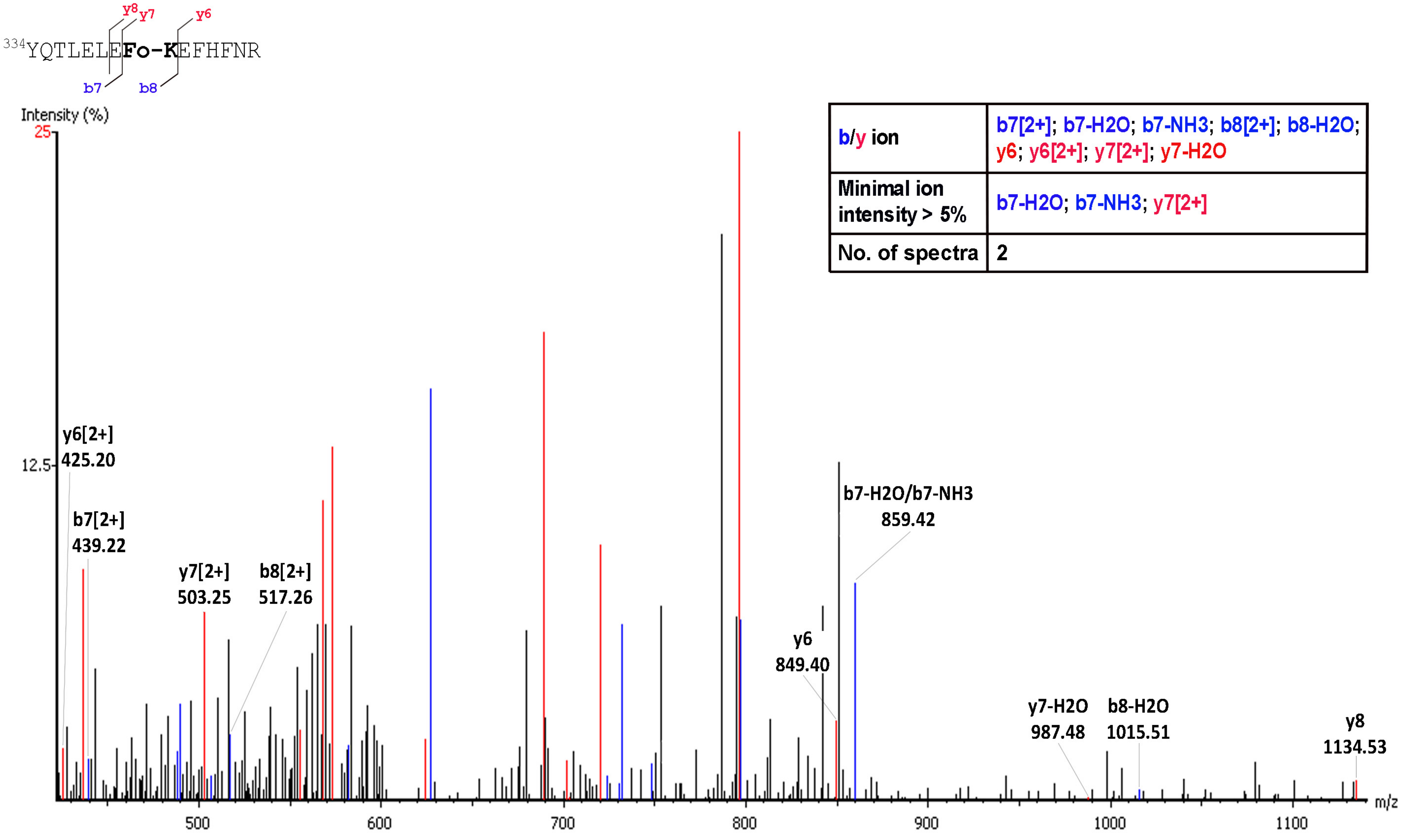

b/y ion	b7[2+]; b7-H2O; b7-NH3; b8[2+]; b8-H2O; y6; y6[2+]; y7[2+]; y7-H2O
Minimal ion intensity > 5%	b7-H2O; b7-NH3; y7[2+]
No. of spectra	2

E MS^2 *m/z* 630.85

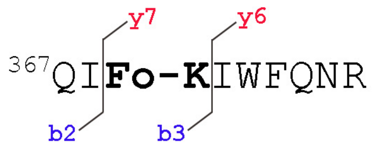

b/y ion	b2; b2-H2O; b2-NH3; b3; b3-H2O; b3-NH3; y6; y6[2+]; y7
Minimal ion intensity > 5%	b2; b3; b3-H2O; b3-NH3; y6; y7
No. of spectra	3

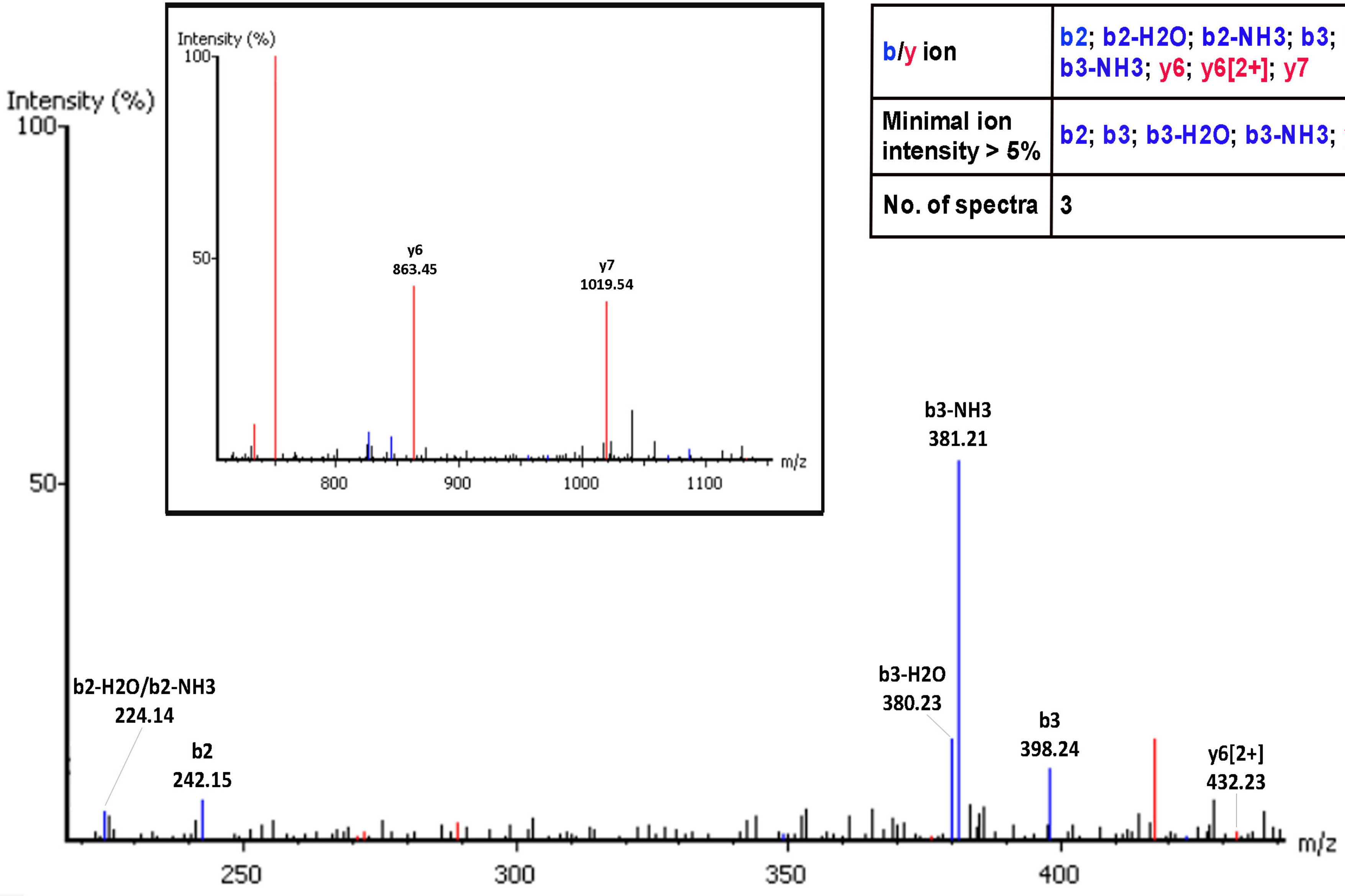

F MS^2 *m/z* 699.29

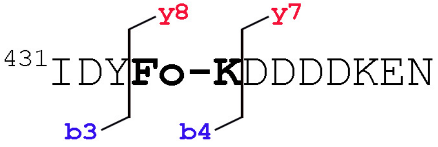

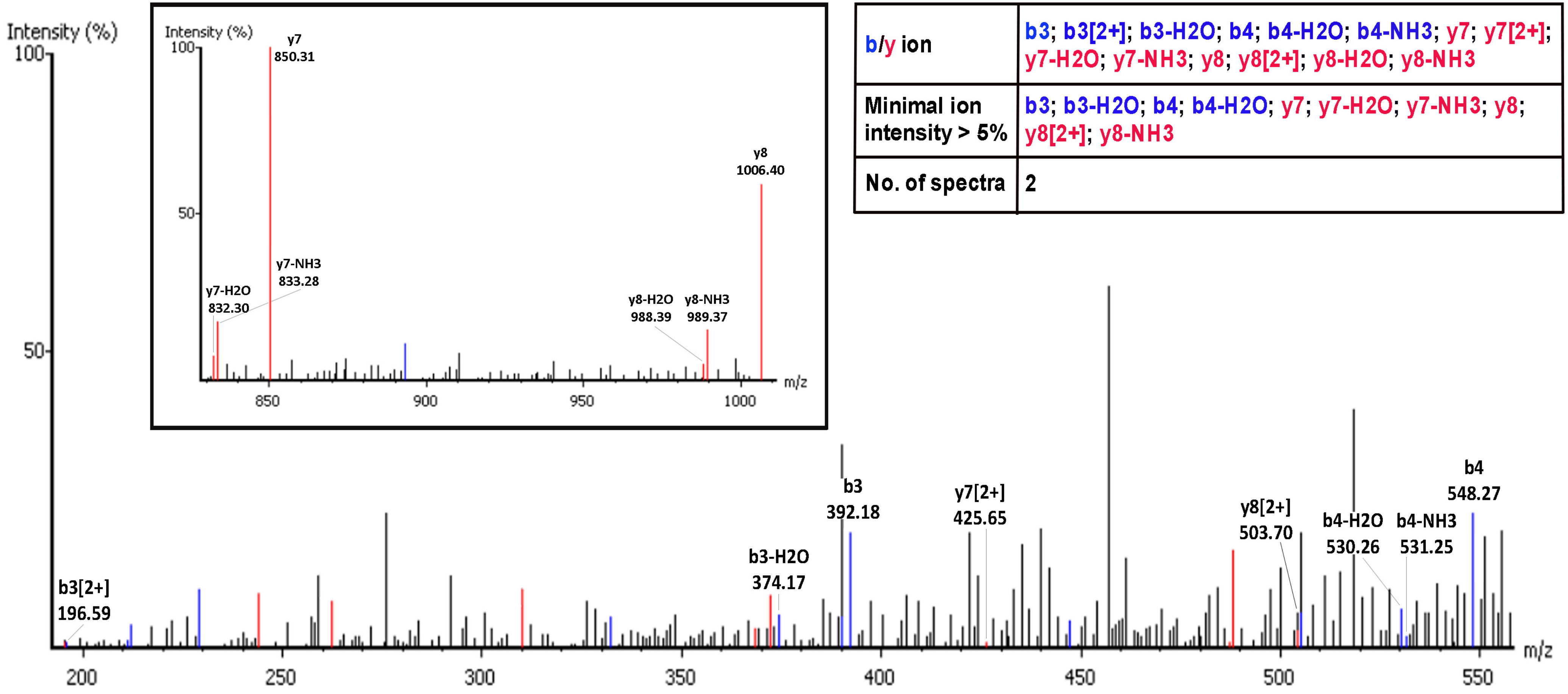

b/y ion	b3; b3[2+]; b3-H2O; b4; b4-H2O; b4-NH3; y7; y7[2+]; y7-H2O; y7-NH3; y8; y8[2+]; y8-H2O; y8-NH3
Minimal ion intensity > 5%	b3; b3-H2O; b4; b4-H2O; y7; y7-H2O; y7-NH3; y8; y8[2+]; y8-NH3
No. of spectra	2

G MS^2 *m/z* 699.29

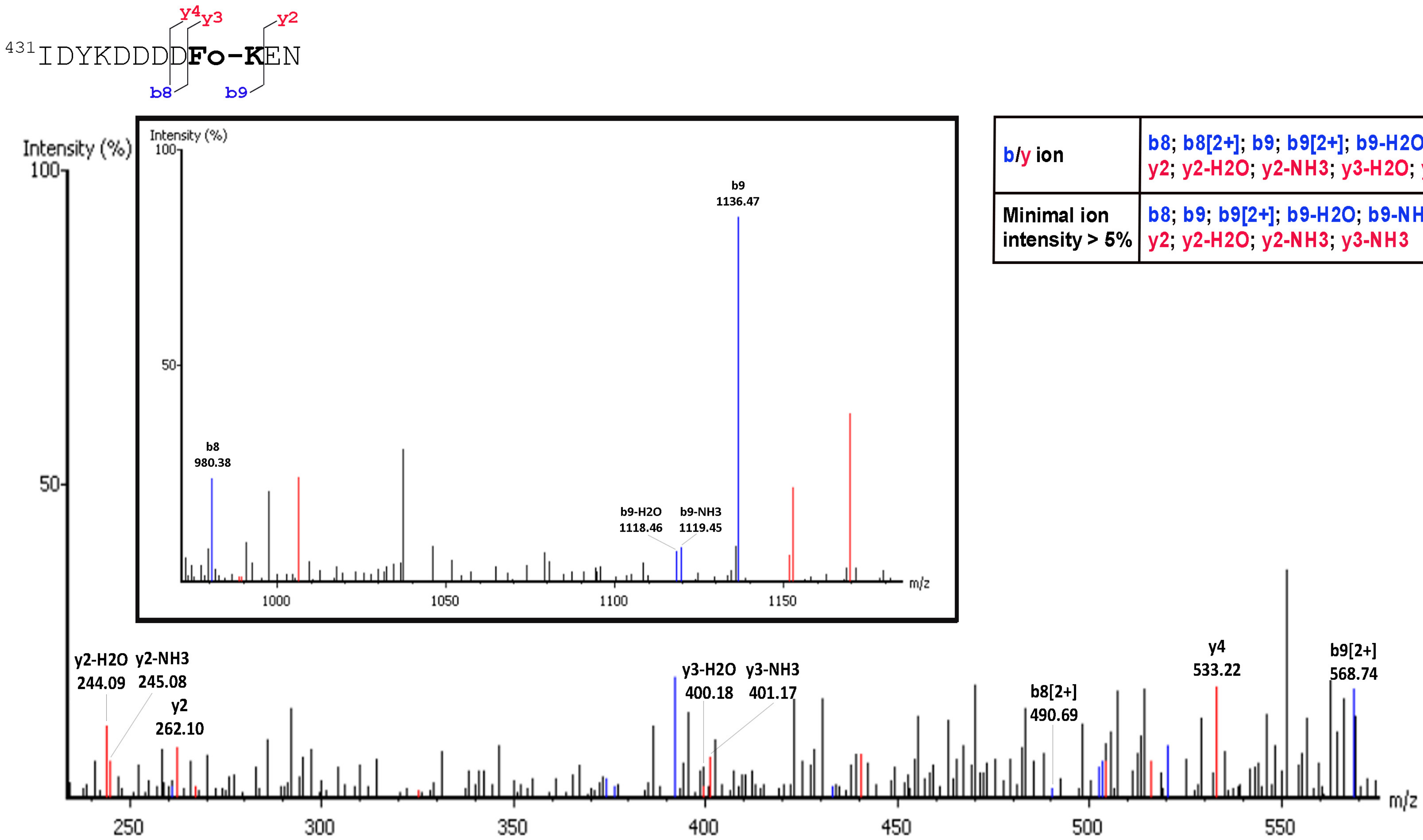

b/y ion	b8; b8[2+]; b9; b9[2+]; b9-H2O; b9-NH3; y2; y2-H2O; y2-NH3; y3-H2O; y3-NH3
Minimal ion intensity > 5%	b8; b9; b9[2+]; b9-H2O; b9-NH3; y2; y2-H2O; y2-NH3; y3-NH3
