# Supplementary material for: Post-translational modifications of Drosophila melanogaster HOX protein, Sex combs reduced
Source: PLoS One. 2020 Jan 13;15(1):e0227642. doi: 10.1371/journal.pone.0227642 (PMC6957346; doi:10.1371/journal.pone.0227642)
Supplement: S7 Fig — MS2 spectra of the peptide identified by LC-MS/MS is shown. (A) Methylation of Lysine 19. (B) Methylation of Serine 166. (C) Methylation of Lysine 168. (D) Methylation of Threonine 364. The inset box shows fragment ions with m/z 1000 to 1150. The peptide sequence and m/z ratio are indicated at the top of the spectra. Positions of fragmentation are shown with vertical lines in the peptide sequence. The box on the right summarizes the evidence confirming methylation. The relevant fragment ions and their m/z ratios supporting methylation are labelled in the spectra. (PDF) [file pone.0227642.s007.pdf]

A MS^2 m/z 714.34

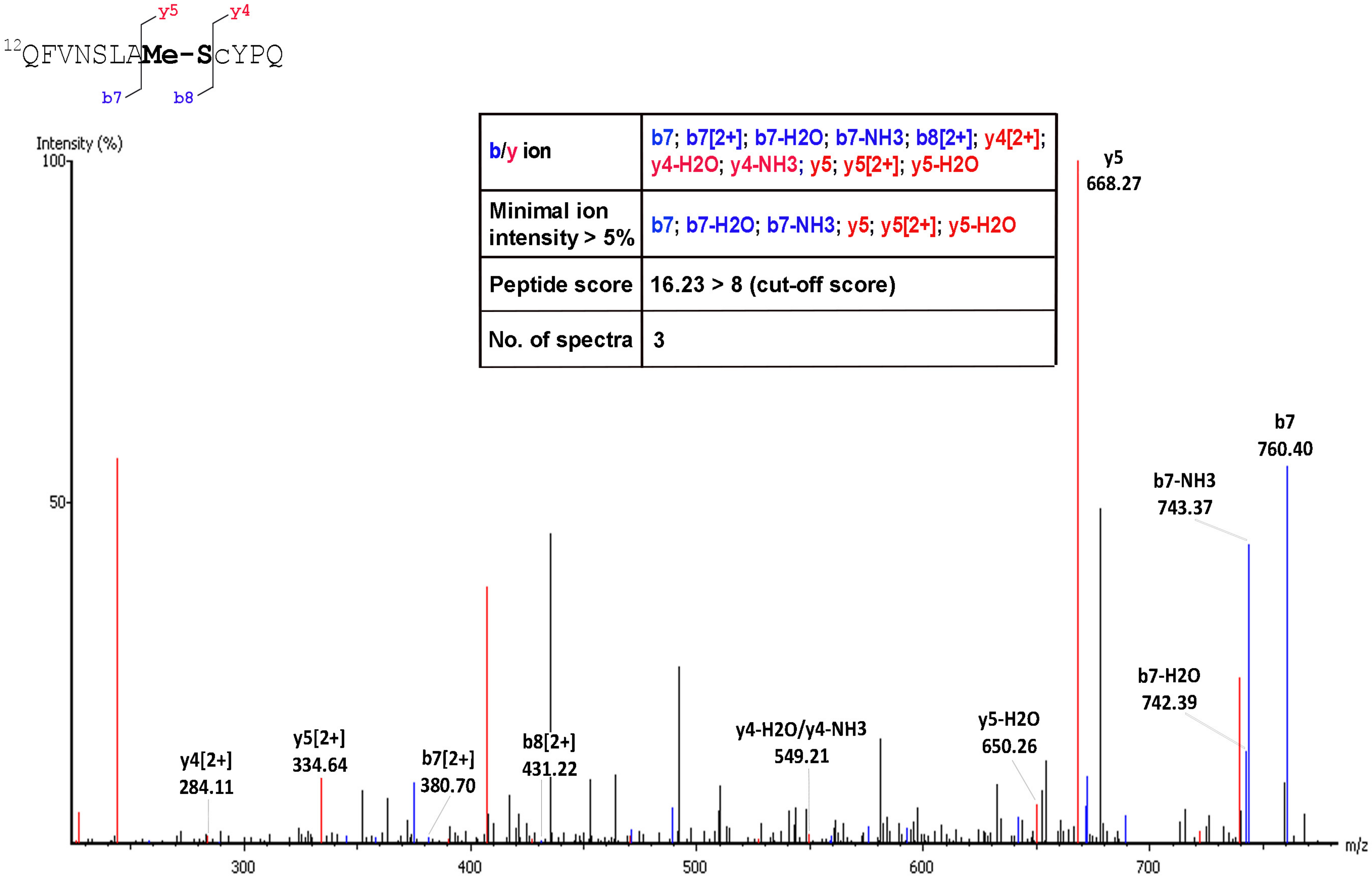

b/y ion	b7; b7[2+]; b7-H2O; b7-NH3; b8[2+]; y4[2+]; y4-H2O; y4-NH3; y5; y5[2+]; y5-H2O
Minimal ion intensity > 5%	b7; b7-H2O; b7-NH3; y5; y5[2+]; y5-H2O
Peptide score	16.23 > 8 (cut-off score)
No. of spectra	3

B **MS² *m/z* 435.21**

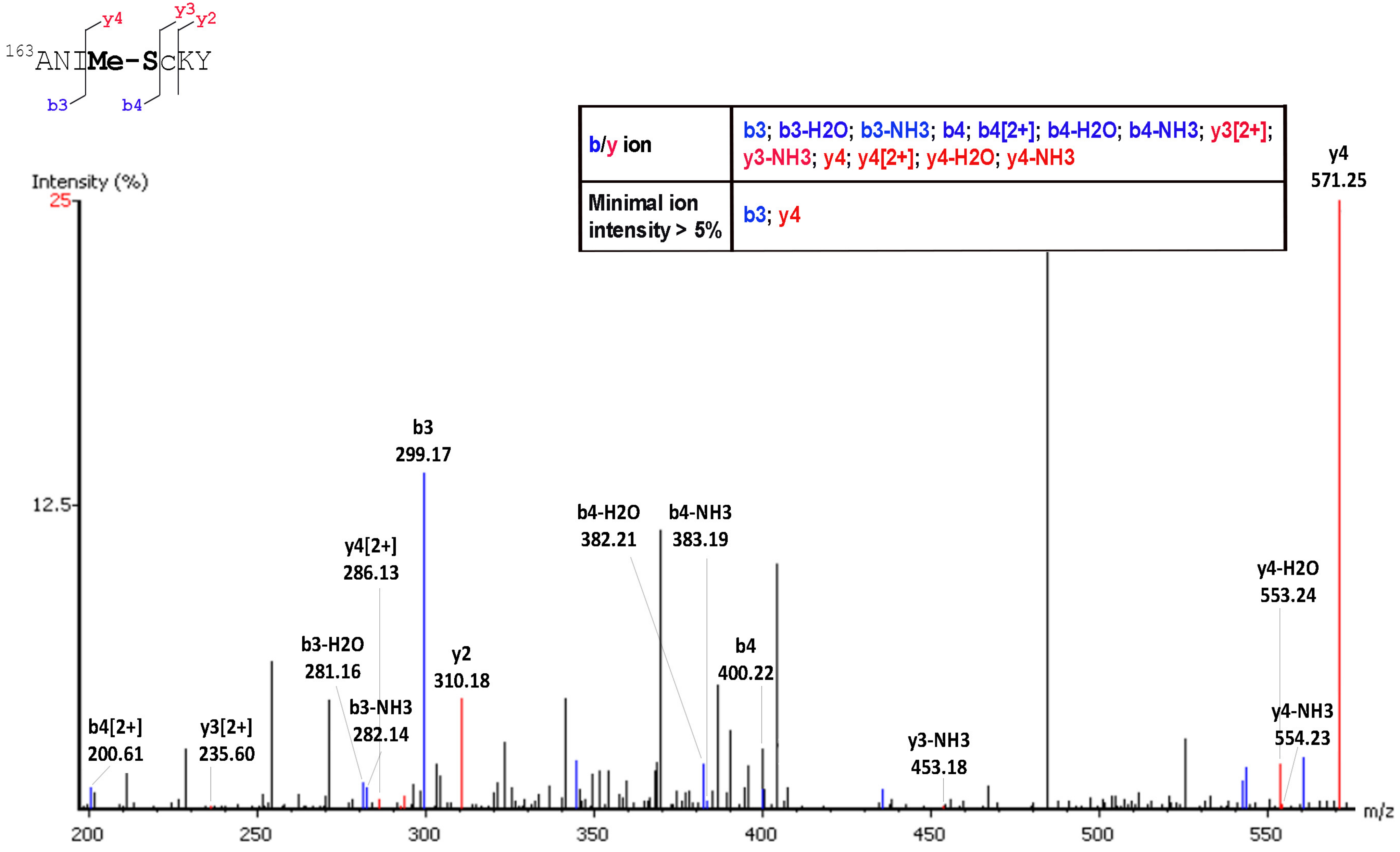

C MS^2 *m/z* 435.21

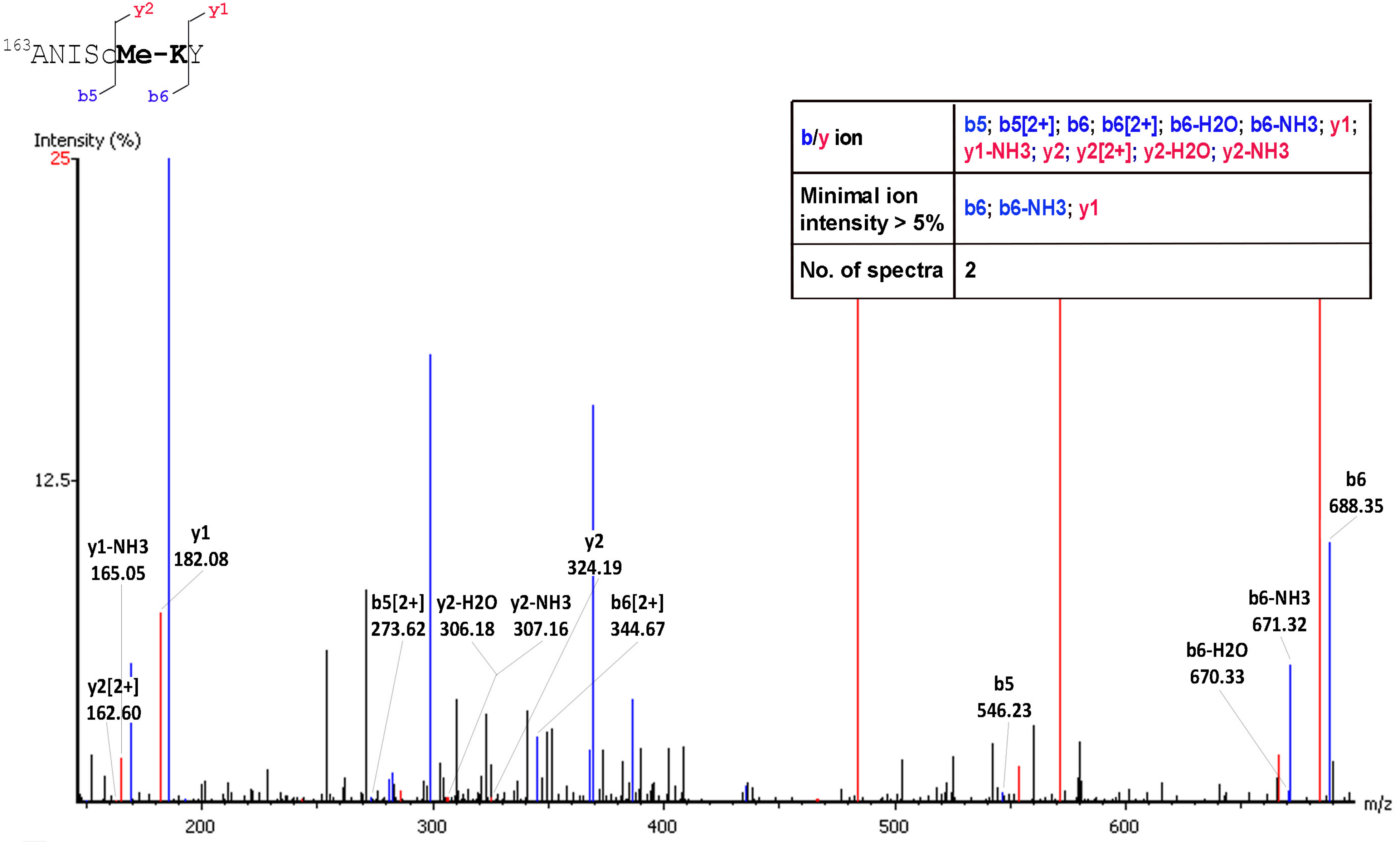

b/y ion	b5; b5[2+]; b6; b6[2+]; b6-H2O; b6-NH3; y1; y1-NH3; y2; y2[2+]; y2-H2O; y2-NH3
Minimal ion intensity > 5%	b6; b6-NH3; y1
No. of spectra	2

D MS² m/z 720.39

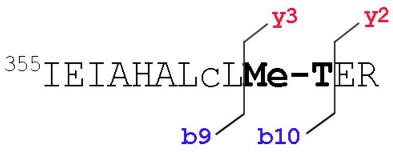

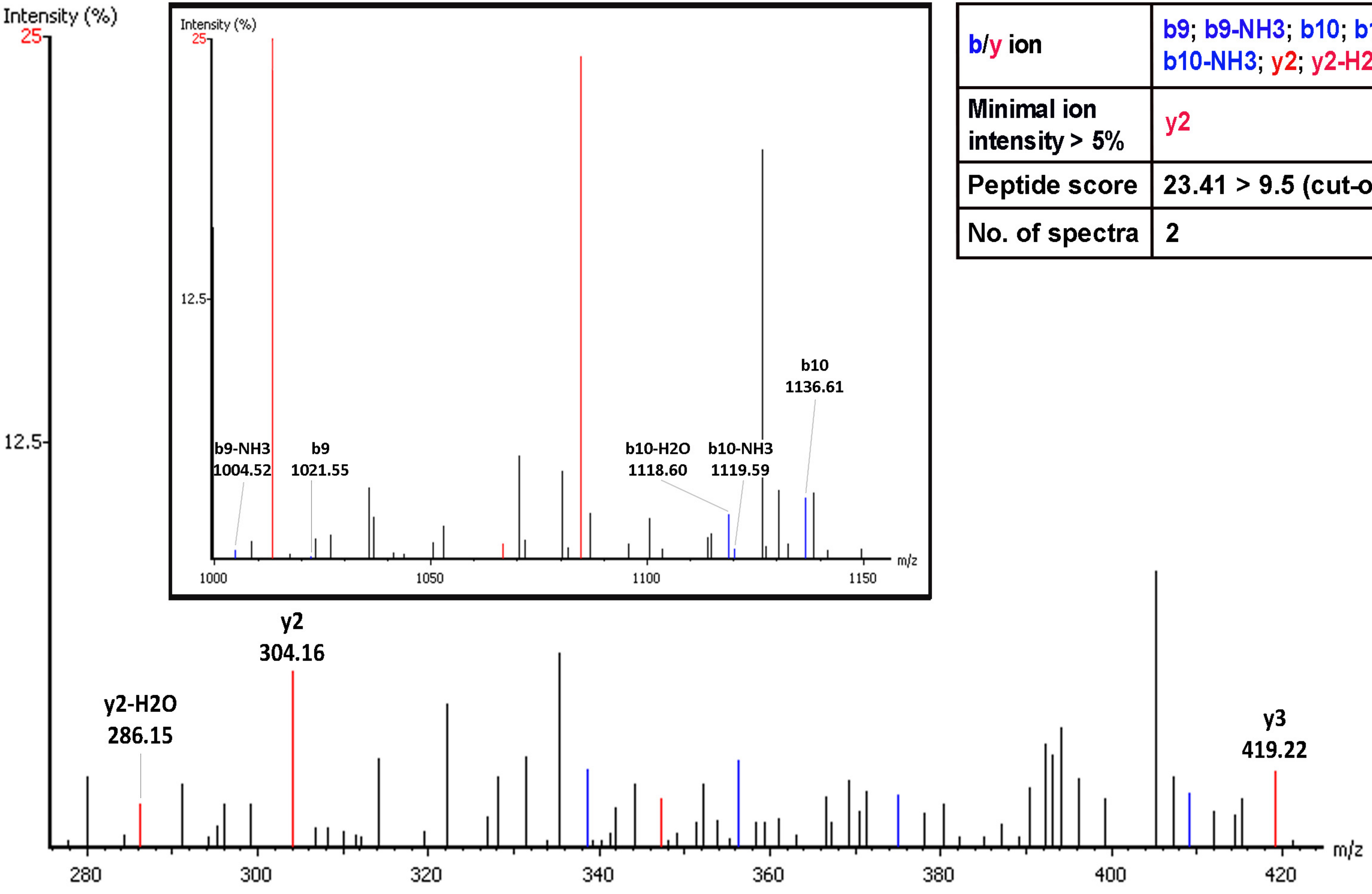

b/y ion	b9; b9-NH3; b10; b10-H2O; b10-NH3; y2; y2-H2O; y3
Minimal ion intensity > 5%	y2
Peptide score	23.41 > 9.5 (cut-off score)
No. of spectra	2
