# Supplementary material for: Post-translational modifications of Drosophila melanogaster HOX protein, Sex combs reduced
Source: PLoS One. 2020 Jan 13;15(1):e0227642. doi: 10.1371/journal.pone.0227642 (PMC6957346; doi:10.1371/journal.pone.0227642)
Supplement: S8 Fig — MS2 spectra of the peptide identified by LC-MS/MS is shown. (A) Carboxylation of Aspartic acid 108. (B) Carboxylation of Lysine 298. The inset box shows fragment ions with m/z 470 to 618. (C) Carboxylation of Tryptophan 307. The inset box shows fragment ions with m/z 780 to 1030. (D) Carboxylation of Lysine 309. The inset box shows fragment ions with m/z 990 to 1300. (E) Carboxylation of Glutamic acid 323. (F) Carboxylation of Lysine 325. (G) Carboxylation of Lysine 369. The inset box shows fragment ions with m/z 840 to 1020. The peptide sequence and m/z ratio are indicated at the top of the spectra. Positions of fragmentation are shown with vertical lines in the peptide sequence. The box on the right summarizes the evidence confirming carboxylation. The relevant fragment ions and their m/z ratios supporting carboxylation are labelled in the spectra. (PDF) [file pone.0227642.s008.pdf]

A MS^2 *m/z* 827.71

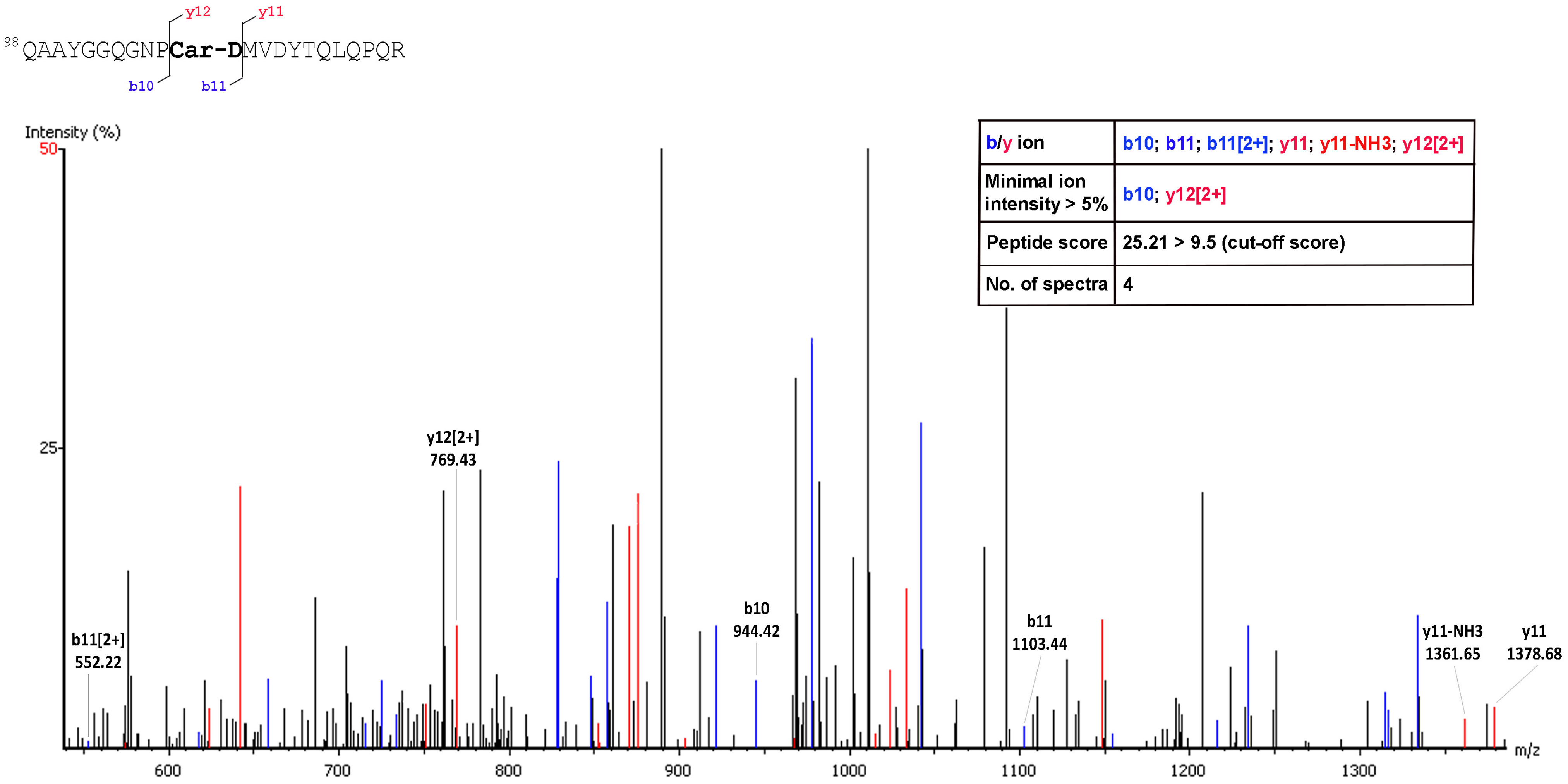

b/y ion	b10; b11; b11[2+]; y11; y11-NH3; y12[2+]
Minimal ion intensity > 5%	b10; y12[2+]
Peptide score	25.21 > 9.5 (cut-off score)
No. of spectra	4

B MS^2 *m/z* 815.4

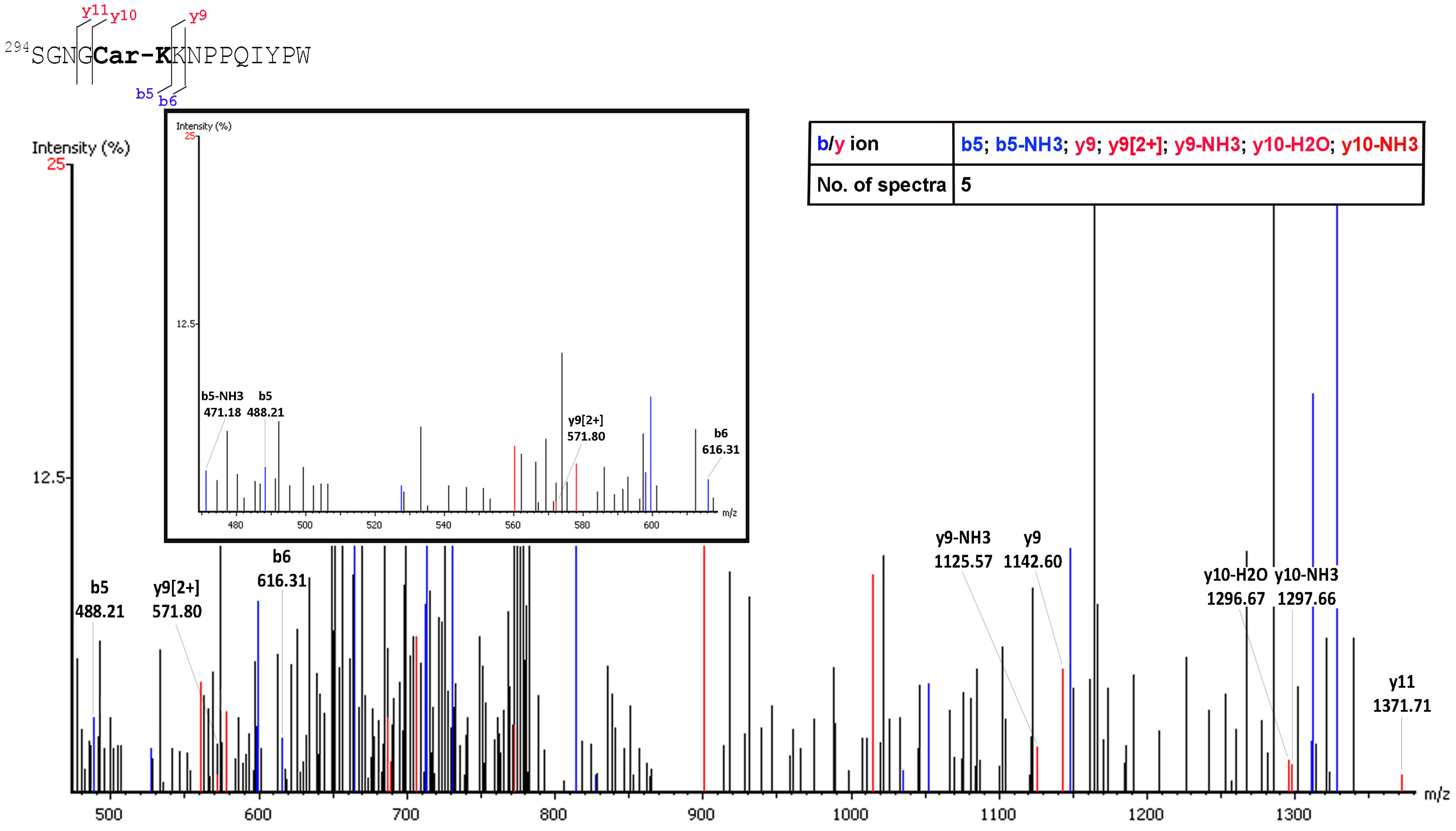

y11 y10 y9
294SGNGCar-KKNPPQIYPW
b5 b6
Intensity (%)
25
12.5
b5 488.21
y9[2+] 571.80
b6 616.31
y9-NH3 1125.57
y9 1142.60
y10-H2O 1296.67
y10-NH3 1297.66
y11 1371.71
m/z
500 600 700 800 900 1000 1100 1200 1300
b5-NH3 471.18
b5 488.21
y9[2+] 571.80
b6 616.31
480 500 520 540 560 580 600
b/y ion: b5; b5-NH3; y9; y9[2+]; y9-NH3; y10-H2O; y10-NH3
No. of spectra: 5

C MS^2 *m/z* 491.92

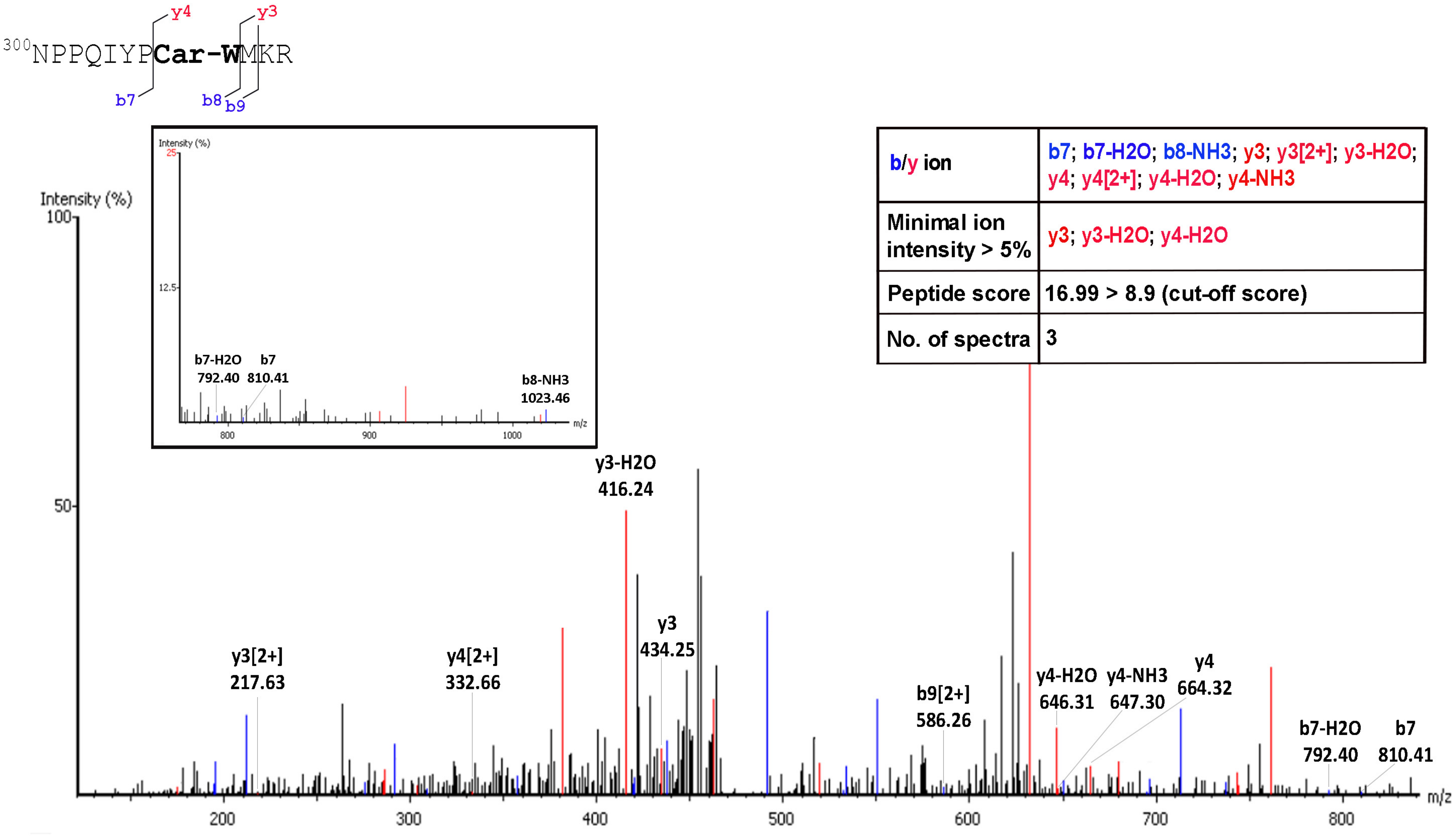

b/y ion	b7; b7-H2O; b8-NH3; y3; y3[2+]; y3-H2O; y4; y4[2+]; y4-H2O; y4-NH3
Minimal ion intensity > 5%	y3; y3-H2O; y4-H2O
Peptide score	16.99 > 8.9 (cut-off score)
No. of spectra	3

D MS^2 *m/z* 737.37

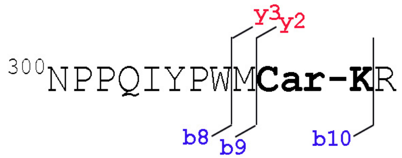

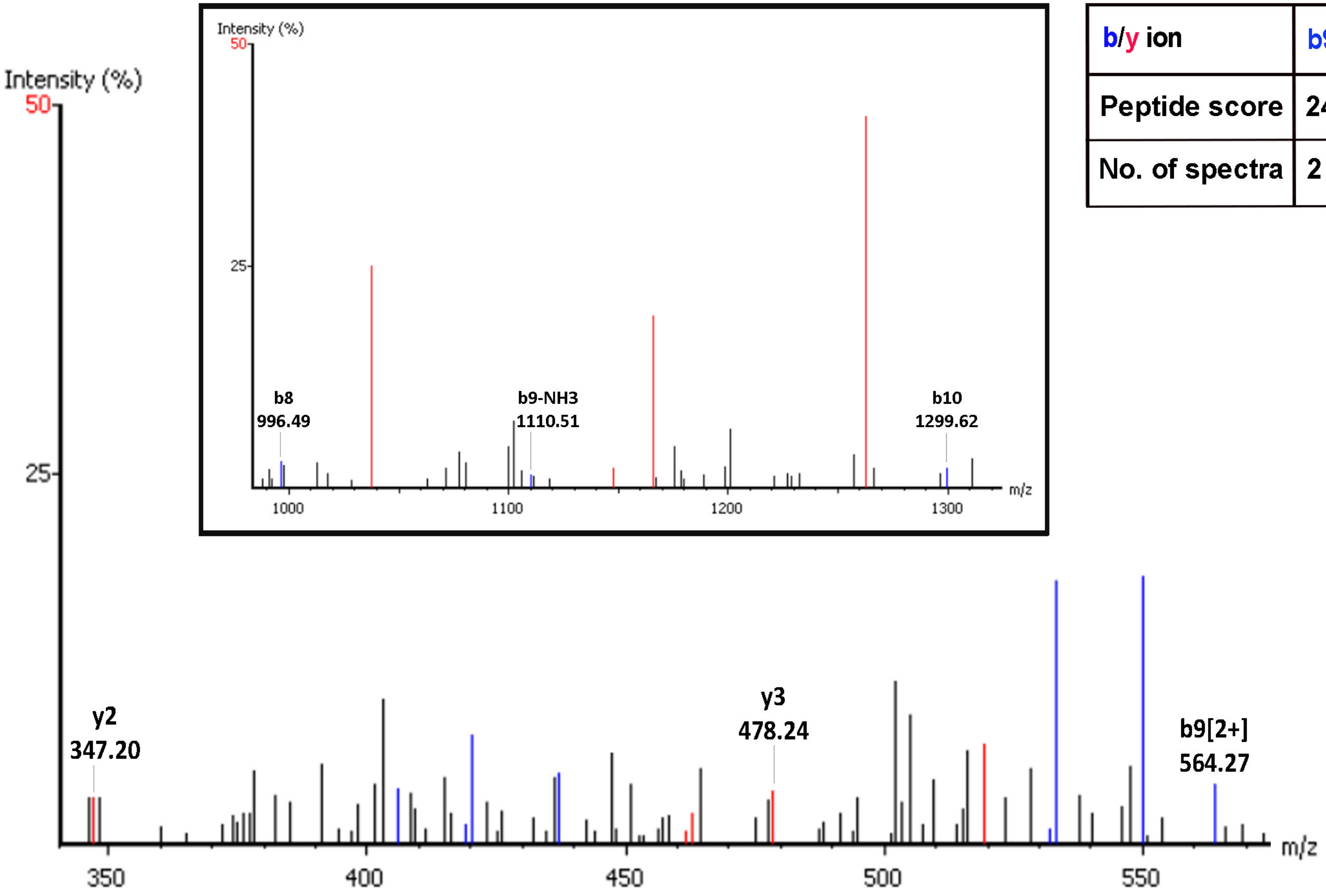

b/y ion	b9[2+]; b9-NH3; b10; y2
Peptide score	24.22 > 11 (cut-off score)
No. of spectra	2

E MS² *m/z* 864.93

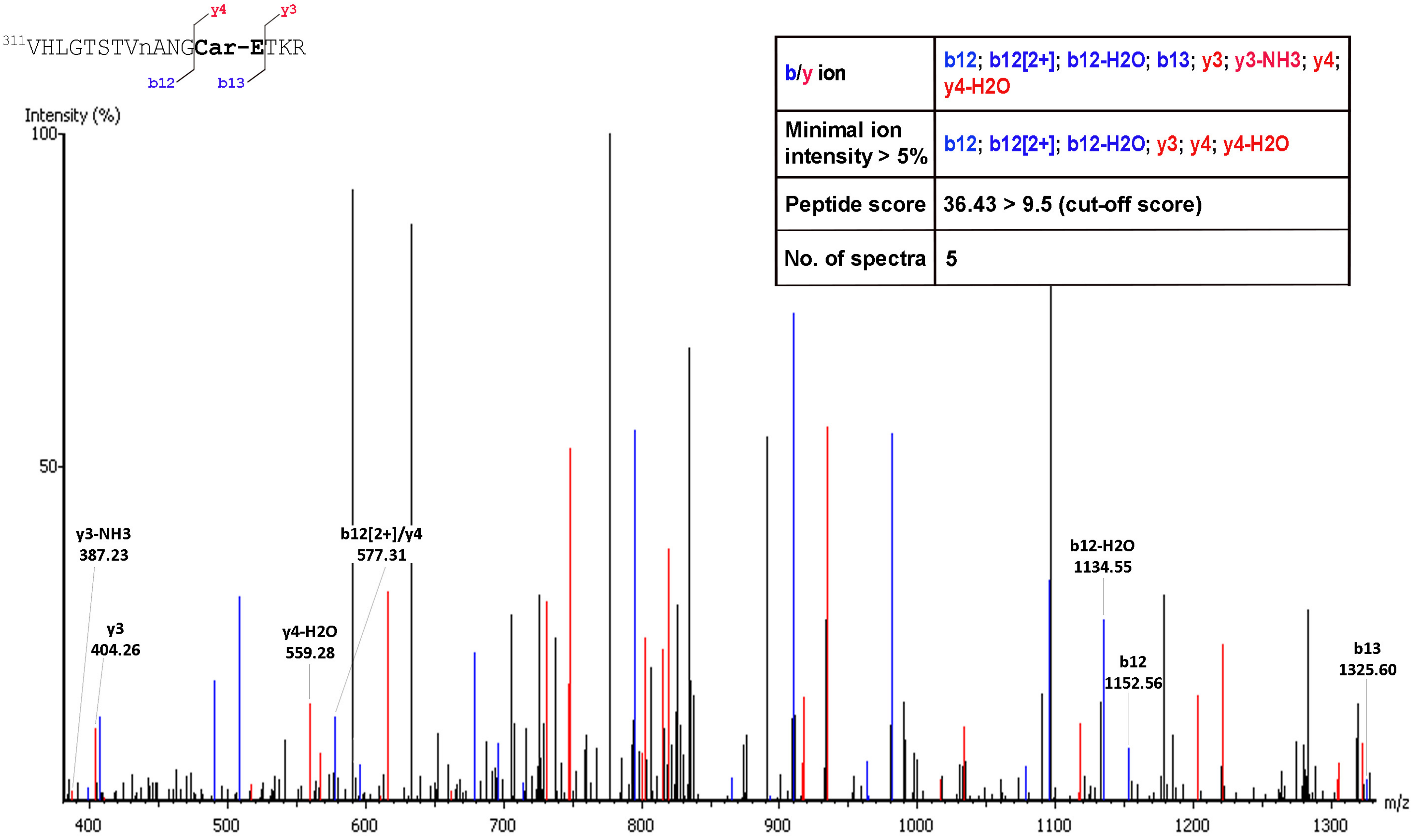

b/y ion	b12; b12[2+]; b12-H2O; b13; y3; y3-NH3; y4; y4-H2O
Minimal ion intensity > 5%	b12; b12[2+]; b12-H2O; y3; y4; y4-H2O
Peptide score	36.43 > 9.5 (cut-off score)
No. of spectra	5

F MS^2 m/z 595.63

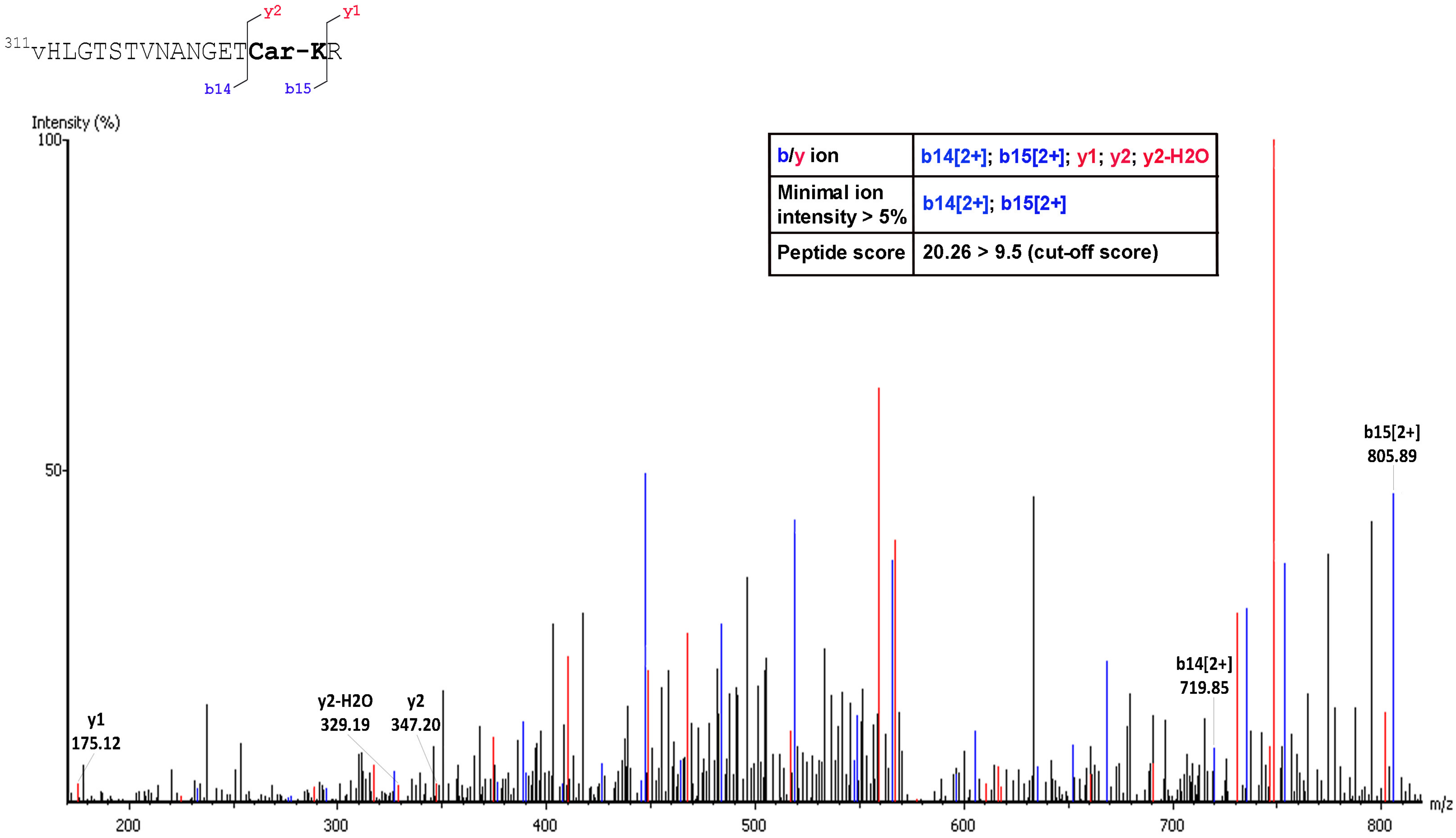

b/y ion	b14[2+]; b15[2+]; y1; y2; y2-H2O
Minimal ion intensity > 5%	b14[2+]; b15[2+]
Peptide score	20.26 > 9.5 (cut-off score)

G MS^2 *m/z* 638.85

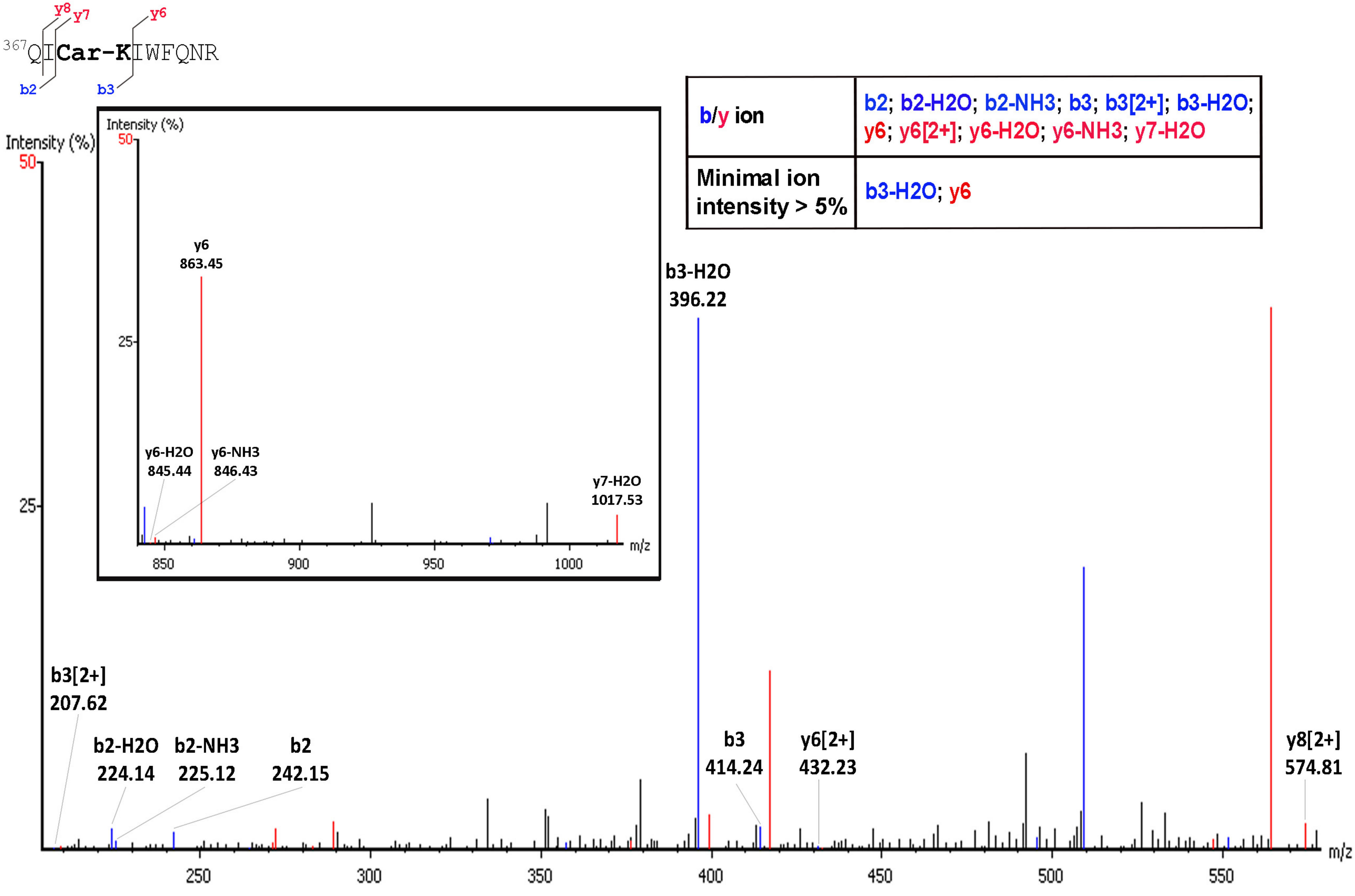

b/y ion	b2; b2-H2O; b2-NH3; b3; b3[2+]; b3-H2O; y6; y6[2+]; y6-H2O; y6-NH3; y7-H2O
Minimal ion intensity > 5%	b3-H2O; y6
