# Supplementary material for: Post-translational modifications of Drosophila melanogaster HOX protein, Sex combs reduced
Source: PLoS One. 2020 Jan 13;15(1):e0227642. doi: 10.1371/journal.pone.0227642 (PMC6957346; doi:10.1371/journal.pone.0227642)

A MS^2 *m/z* 801.35

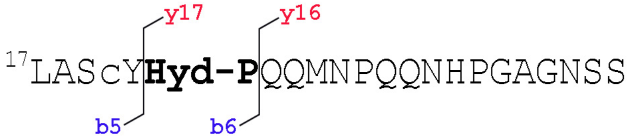

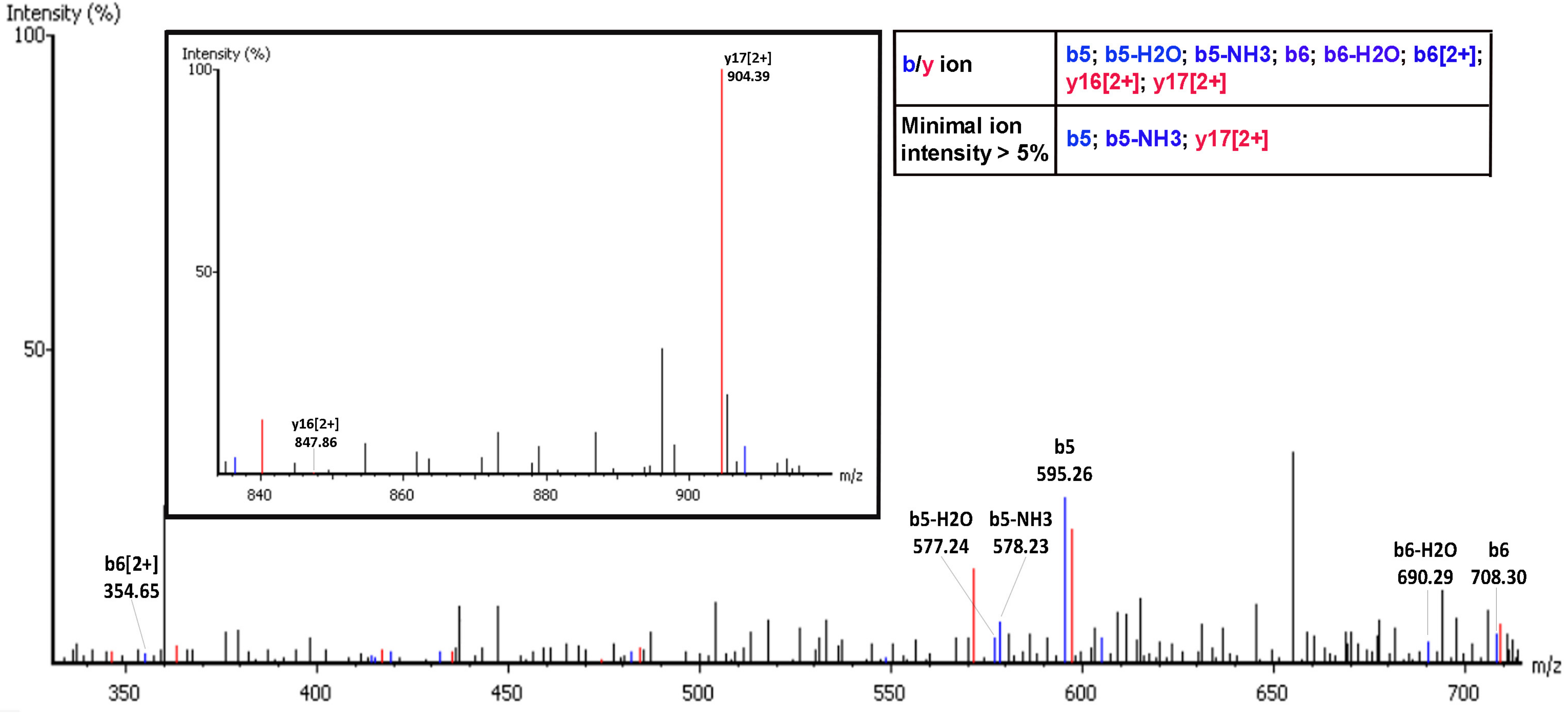

b/y ion	b5; b5-H2O; b5-NH3; b6; b6-H2O; b6[2+]; y16[2+]; y17[2+]
Minimal ion intensity > 5%	b5; b5-NH3; y17[2+]

B MS^2 *m/z* 766.36

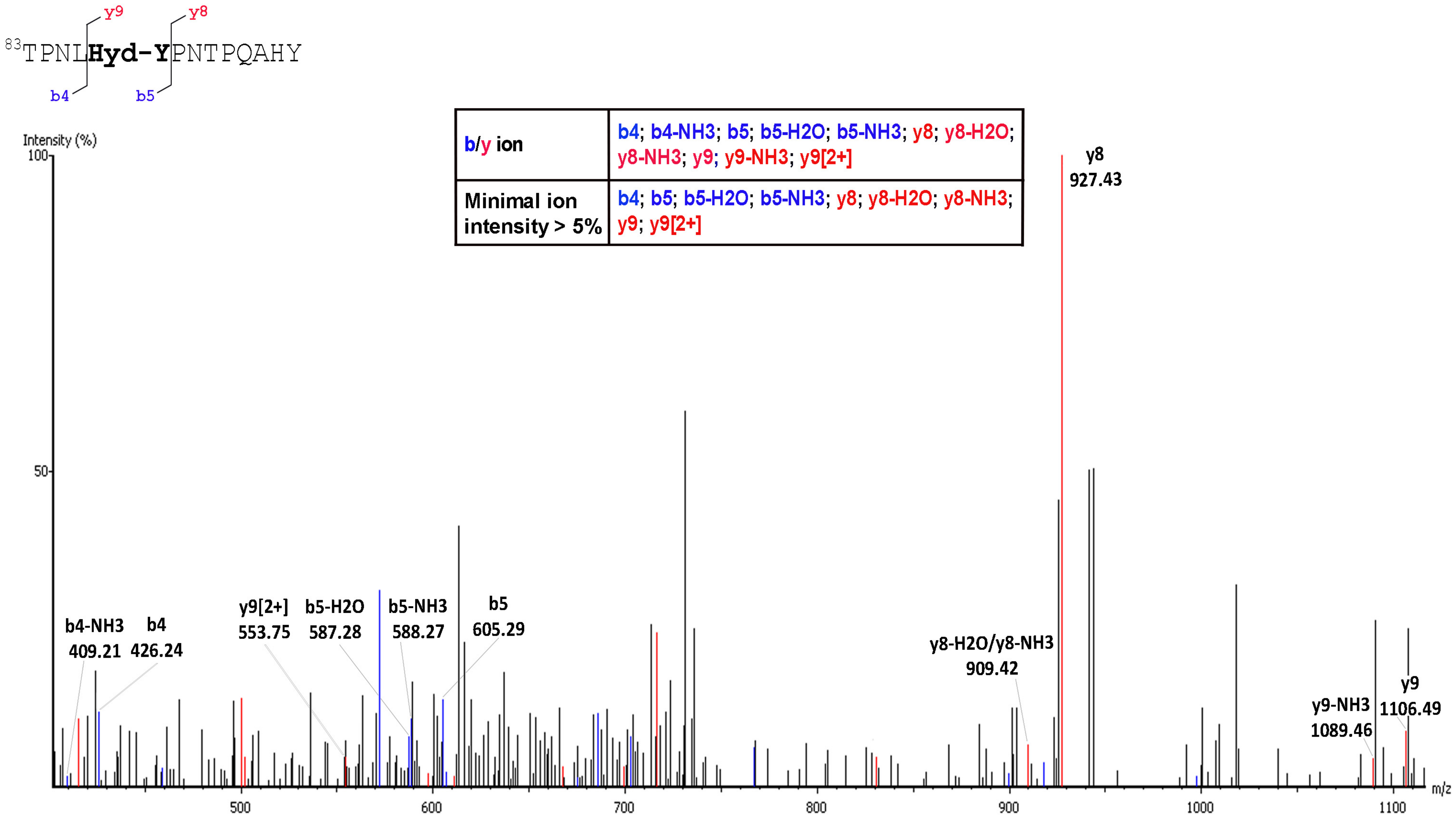

b/y ion	b4; b4-NH3; b5; b5-H2O; b5-NH3; y8; y8-H2O; y8-NH3; y9; y9-NH3; y9[2+]
Minimal ion intensity > 5%	b4; b5; b5-H2O; b5-NH3; y8; y8-H2O; y8-NH3; y9; y9[2+]

C MS^2 *m/z* 780.82

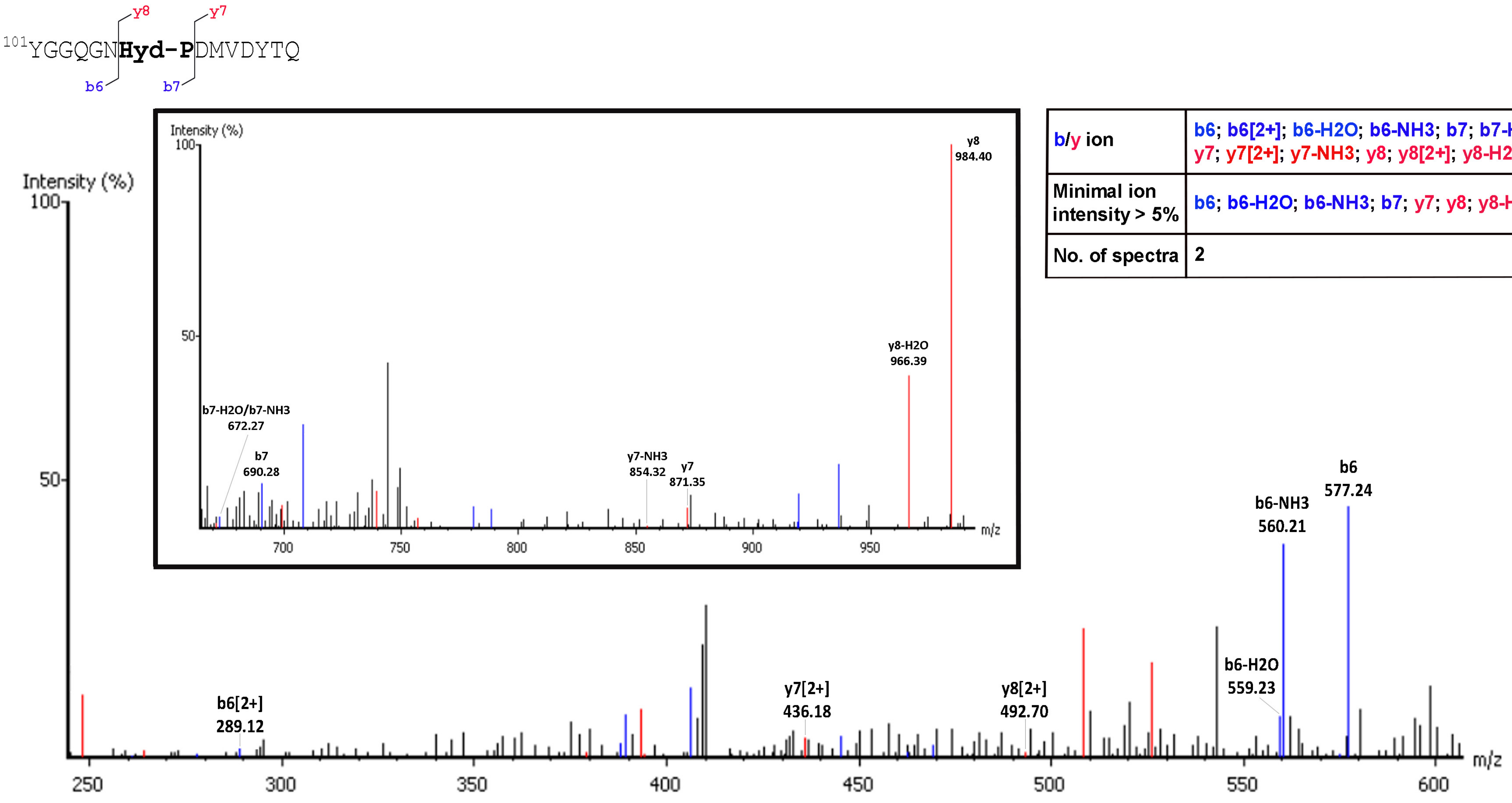

b/y ion	b6; b6[2+]; b6-H2O; b6-NH3; b7; b7-H2O; b7-NH3; y7; y7[2+]; y7-NH3; y8; y8[2+]; y8-H2O
Minimal ion intensity > 5%	b6; b6-H2O; b6-NH3; b7; y7; y8; y8-H2O
No. of spectra	2

D MS^2 *m/z* 699.29

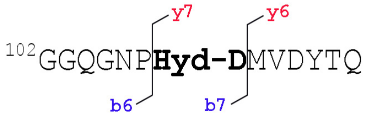

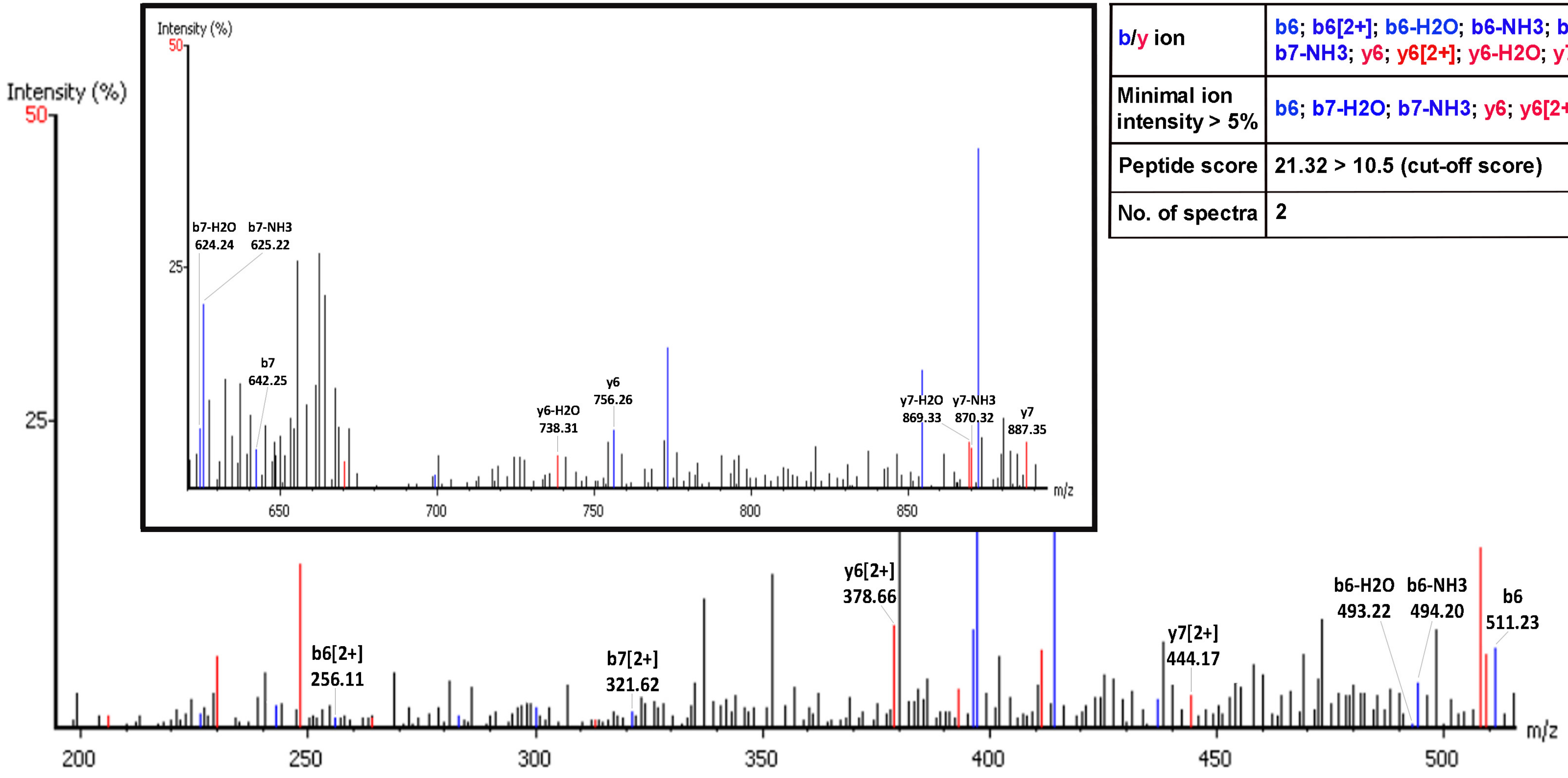

b/y ion	b6; b6[2+]; b6-H2O; b6-NH3; b7; b7[2+]; b7-H2O; b7-NH3; y6; y6[2+]; y6-H2O; y7; y7[2+]; y7-H2O; y7-NH3
Minimal ion intensity > 5%	b6; b7-H2O; b7-NH3; y6; y6[2+]; y7; y7-H2O
Peptide score	21.32 > 10.5 (cut-off score)
No. of spectra	2

E MS² *m/z* 673.98

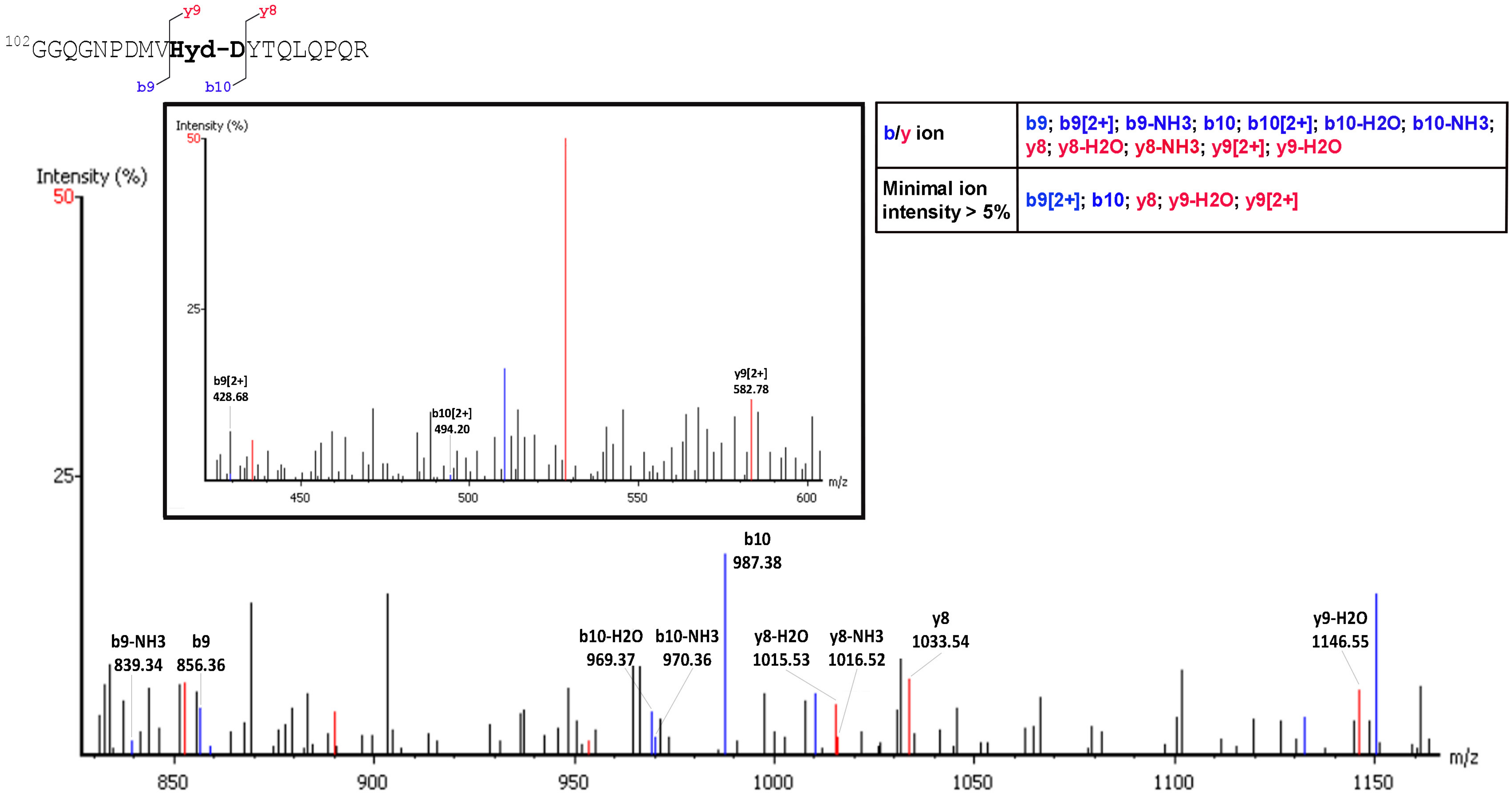

b/y ion	b9; b9[2+]; b9-NH3; b10; b10[2+]; b10-H2O; b10-NH3; y8; y8-H2O; y8-NH3; y9[2+]; y9-H2O
Minimal ion intensity > 5%	b9[2+]; b10; y8; y9-H2O; y9[2+]

F MS^2 *m/z* 1095.43

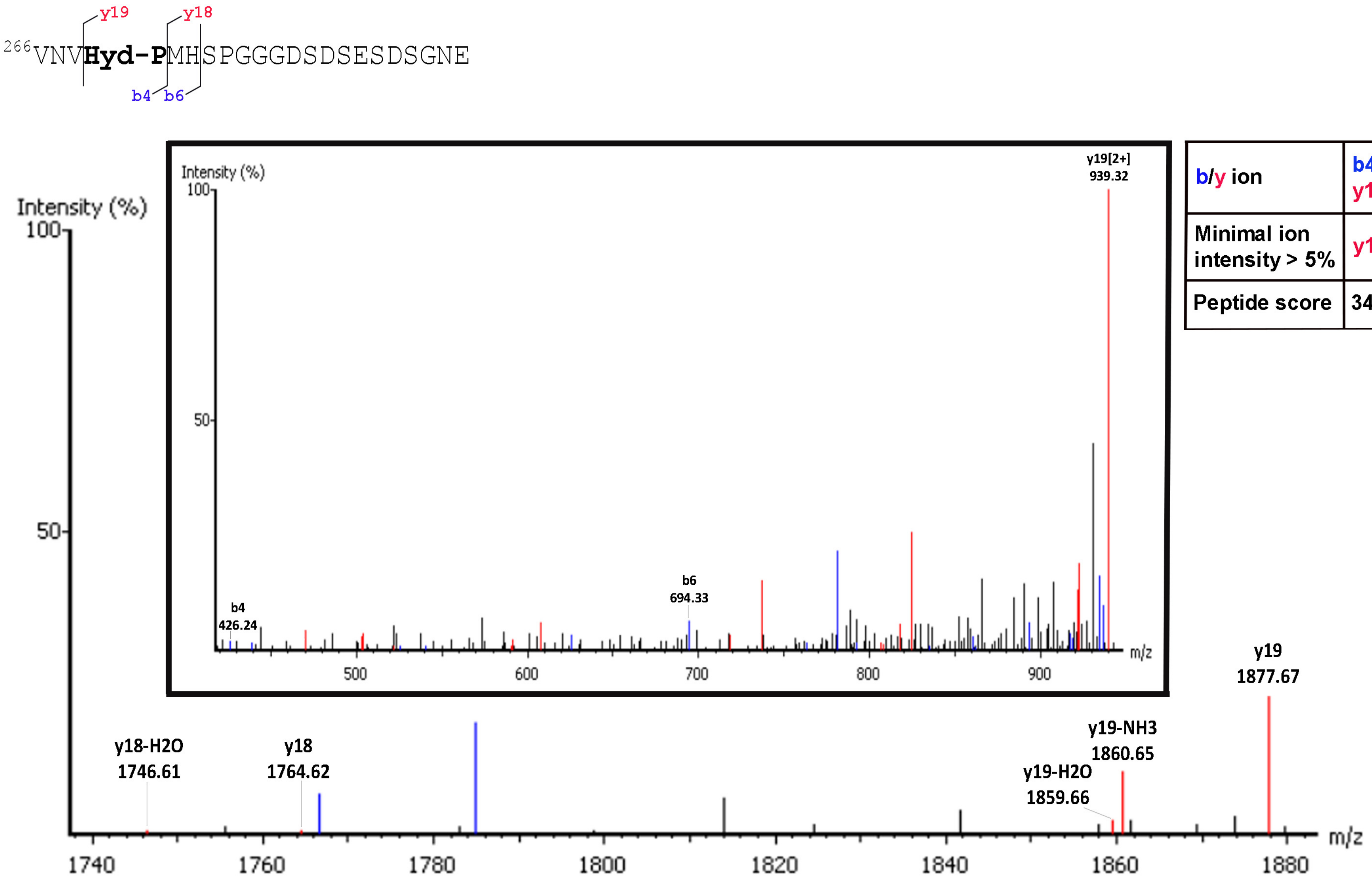

b/y ion	b4; y18; y18-H2O; y19; y19[2+]; y19-H2O; y19-NH3
Minimal ion intensity > 5%	y19; y19[2+]; y19-NH3
Peptide score	34.16 > 17.9 (cut-off score)

G **MS^2 *m/z* 645.32**

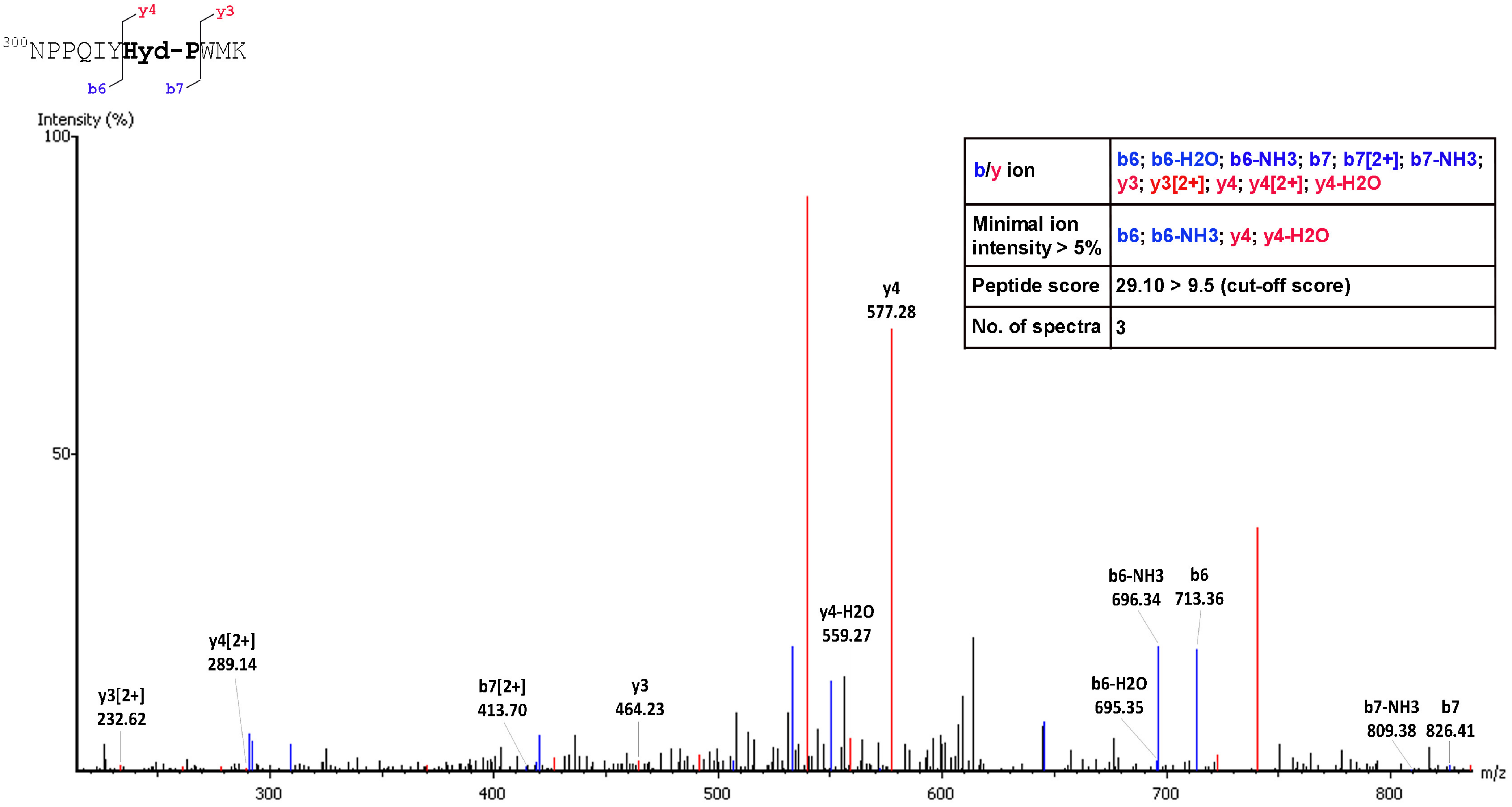

b/y ion	b6; b6-H2O; b6-NH3; b7; b7[2+]; b7-NH3; y3; y3[2+]; y4; y4[2+]; y4-H2O
Minimal ion intensity > 5%	b6; b6-NH3; y4; y4-H2O
Peptide score	29.10 > 9.5 (cut-off score)
No. of spectra	3

H MS^2 *m/z* 482.58

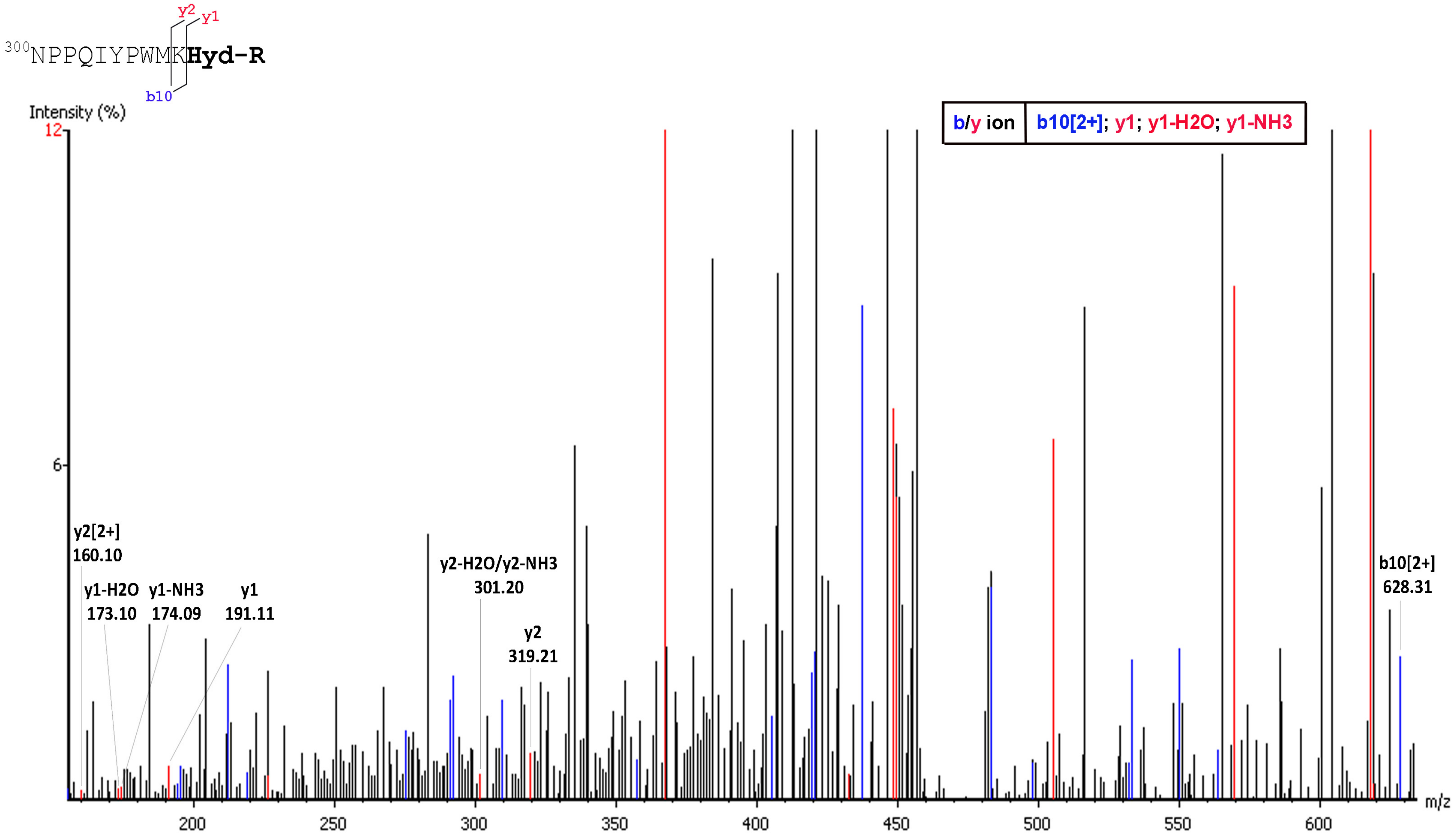

I MS^2 *m/z* 586.3

^{311}VHLGTSTVNA**Hyd-N**GeTKR (fragment cleavage marks: b10, b11, y6, y4)

b/y ion	b10; b10[2+]; b10-H2O; b10- NH3; b11[2+]; y6; y6[2+]; y6-NH3
Minimal ion intensity > 5%	b11[2+]; y6; y6[2+]; y6-NH3

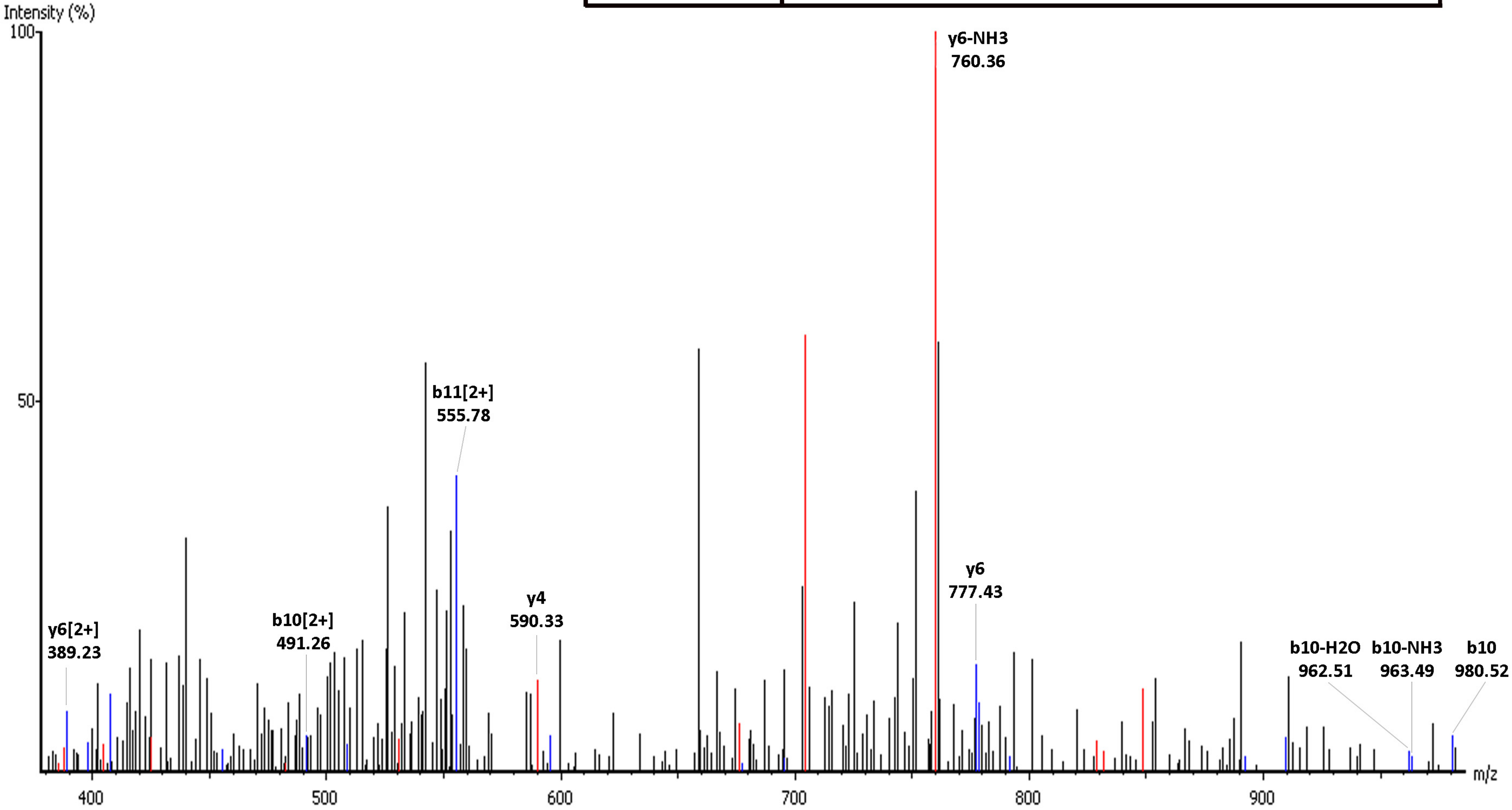

J MS^2 *m/z* 567.62

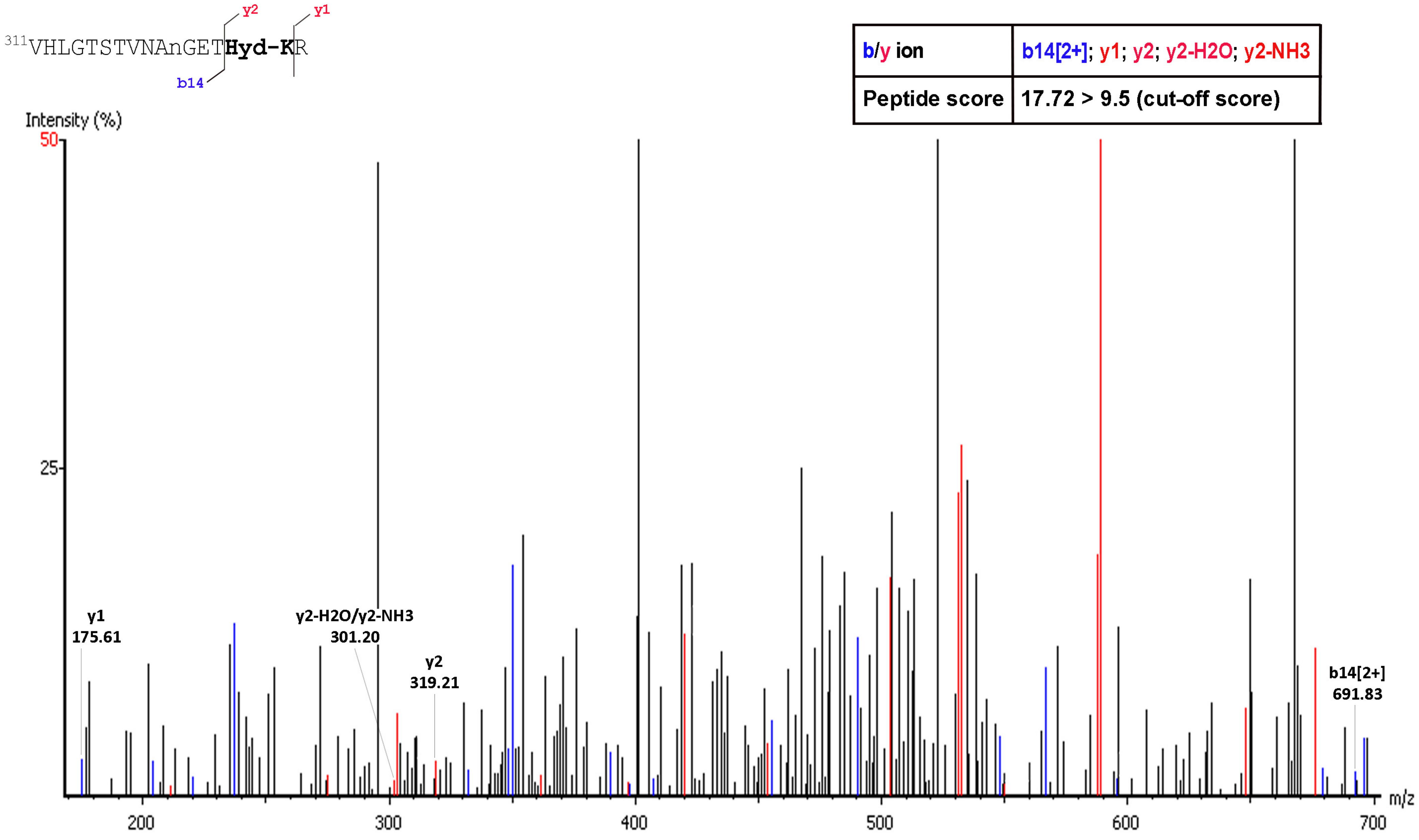

K MS^2 *m/z* 549.95

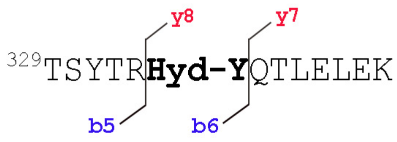

b/y ion	b5; b5[2+]; b5-H2O; b5-NH3; b6; b6[2+]; y7; y7[2+]; y7-H2O; y8; y8[2+]; y8-H2O
No. of spectra	2

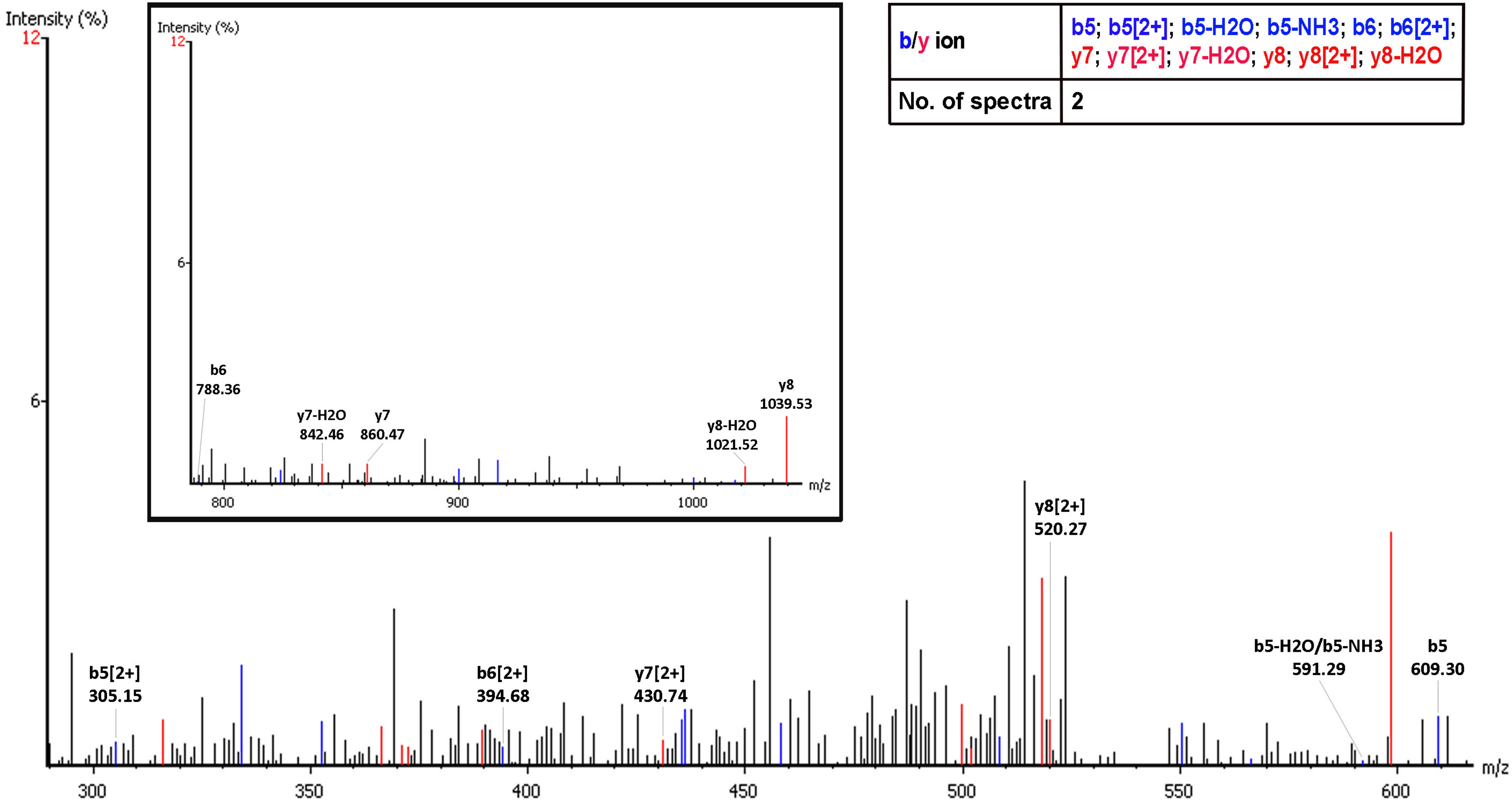

L MS^2 *m/z* 481.25

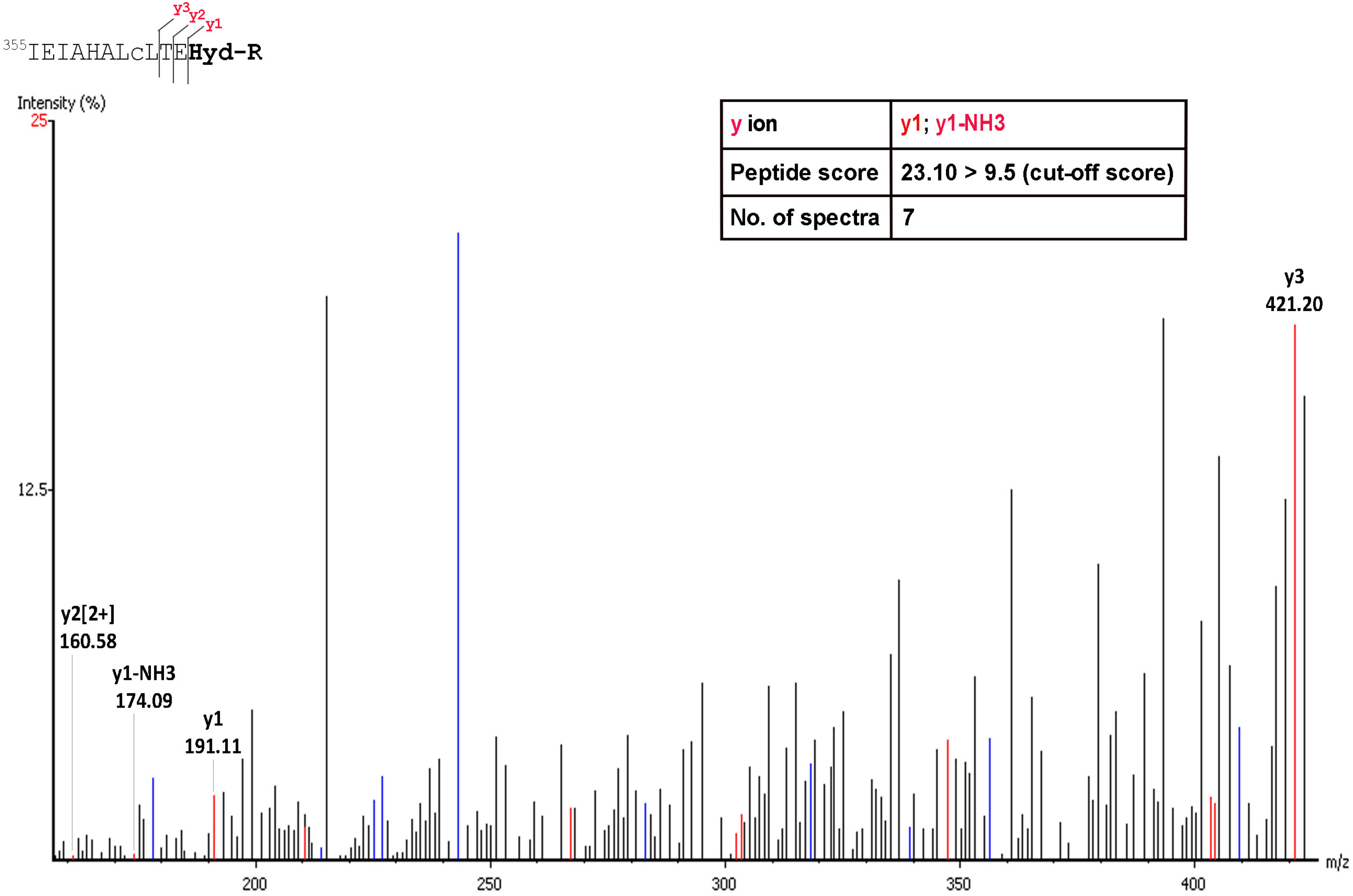

y ion	y1; y1-NH3
Peptide score	23.10 > 9.5 (cut-off score)
No. of spectra	7

M MS^2 m/z 603.78

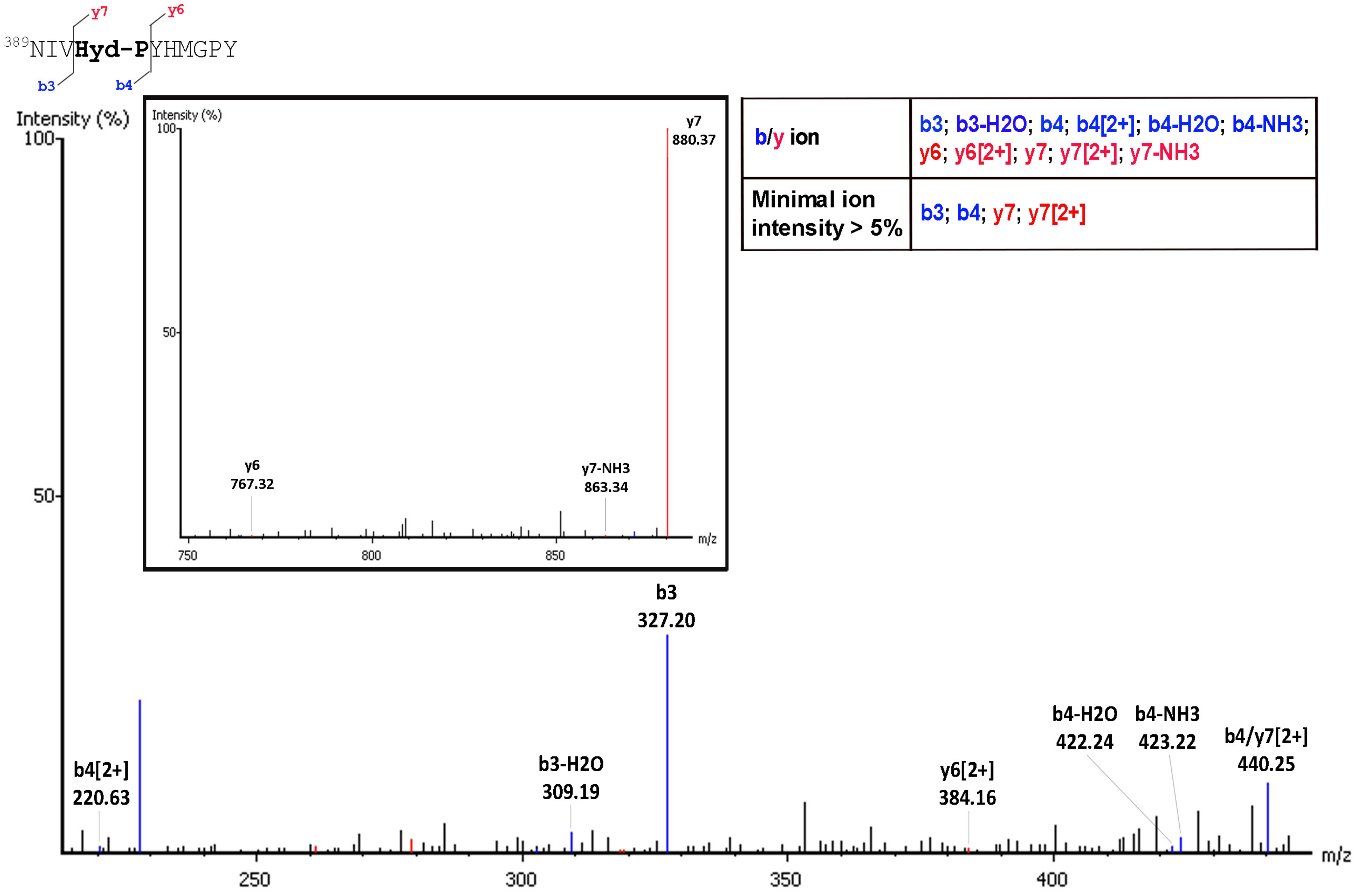

b/y ion	b3; b3-H2O; b4; b4[2+]; b4-H2O; b4-NH3; y6; y6[2+]; y7; y7[2+]; y7-NH3
Minimal ion intensity > 5%	b3; b4; y7; y7[2+]

N MS^2 *m/z* 603.78

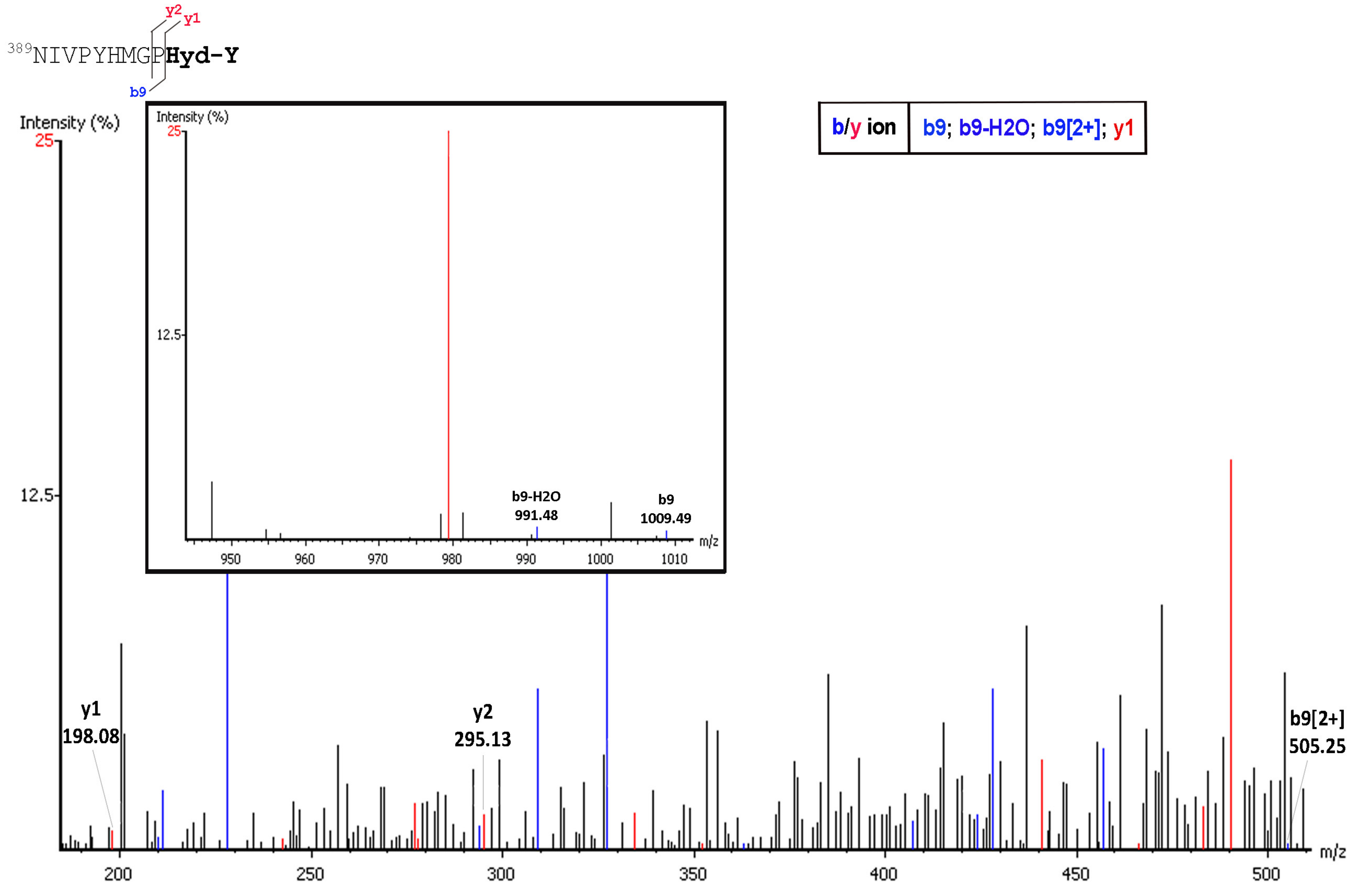

Supplement: S9 Fig — MS2 spectra of the peptide identified by LC-MS/MS is shown. (A) Hydroxylation of Proline 22. The inset box shows fragment ions with m/z 835 to 920. (B) Hydroxylation of Tyrosine 87. (C) Hydroxylation of Proline 107. The inset box shows fragment ions with m/z 670 to 1000. (D) Hydroxylation of Aspartic acid 108. The inset box shows fragment ions with m/z 620 to 900. (E) Hydroxylation of Aspartic acid 111. The inset box shows fragment ions with m/z 420 to 600. (F) Hydroxylation of Proline 269. The inset box shows fragment ions with m/z 420 to 940. (G) Hydroxylation of Proline 306. (H) Hydroxylation of R310. (I) Hydroxylation of N321. (J) Hydroxylation of K325. (K) Hydroxylation of Y334. The inset box shows fragment ions with m/z 788 to 1040. (L) Hydroxylation of R366. (M) Hydroxylation of P392. The inset box shows fragment ions with m/z 750 to 890. (N) Hydroxylation of Y398. The inset box shows fragment ions with m/z 945 to 1010. The peptide sequence and m/z ratio are indicated at the top of the spectra. Positions of fragmentation are shown with vertical lines in the peptide sequence. The box on the right summarizes the evidence confirming hydroxylation. The relevant fragment ions and their m/z ratios supporting hydroxylation are labelled in the spectra. (PDF) [file pone.0227642.s009.pdf]
