# Supplementary figures and images for: Post-translational modifications of Drosophila melanogaster HOX protein, Sex combs reduced
Source: PLoS One. 2020 Jan 13;15(1):e0227642. doi: 10.1371/journal.pone.0227642 (PMC6957346; doi:10.1371/journal.pone.0227642)

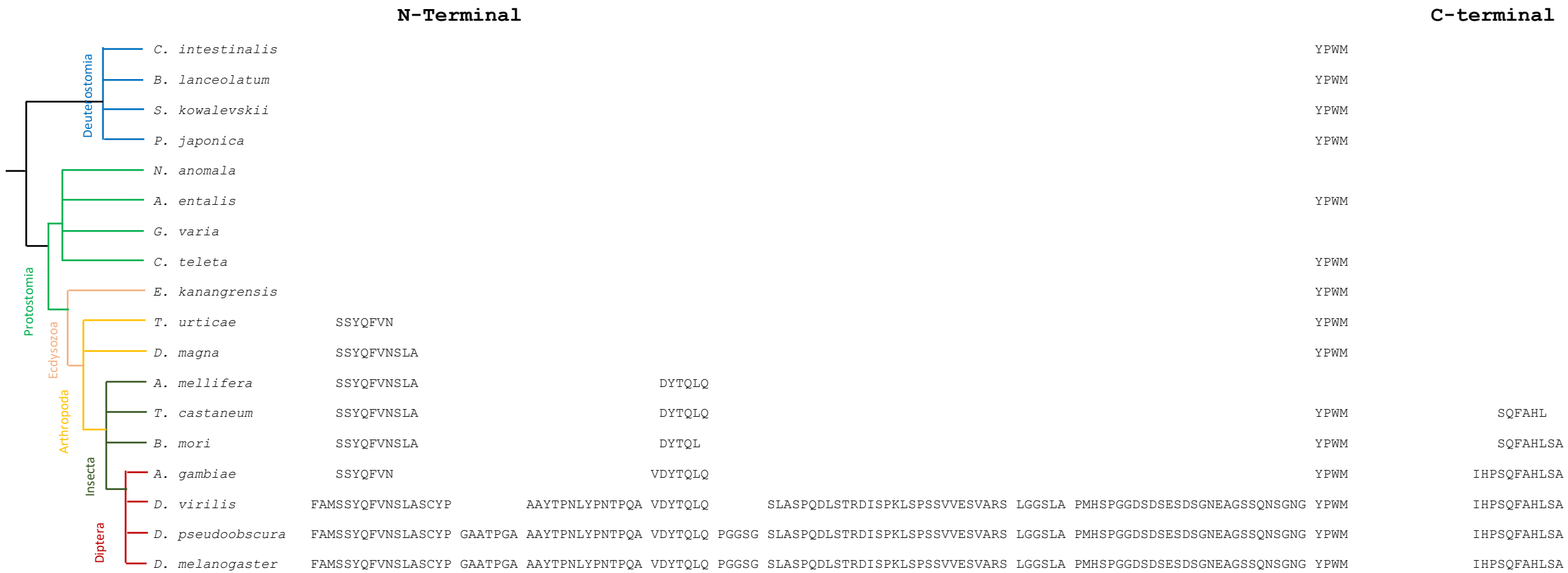

SCR

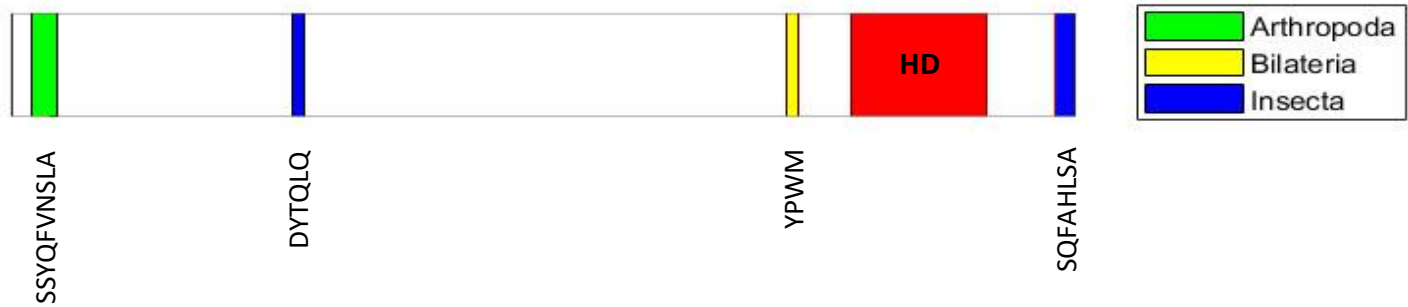

Supplement: S10 Fig — SLiMs of D. melanogaster SCR which are conserved across various taxonomic groups were aligned using multiple sequence alignment tools, MAFFT v. 7 and Clustal Omega. The phylogenetic tree on the left is not drawn to scale and it merely depicts the relationship among the organisms and not evolutionary time of divergence. The block diagram below the aligned sequences shows the HD and four SLiMs conserved beyond Diptera. (PDF) [file pone.0227642.s010.pdf]
