# Supplementary material for: Post-translational modifications of Drosophila melanogaster HOX protein, Sex combs reduced
Source: PLoS One. 2020 Jan 13;15(1):e0227642. doi: 10.1371/journal.pone.0227642 (PMC6957346; doi:10.1371/journal.pone.0227642)
Supplement: S1 Raw Images — (PDF) [file pone.0227642.s011.pdf]

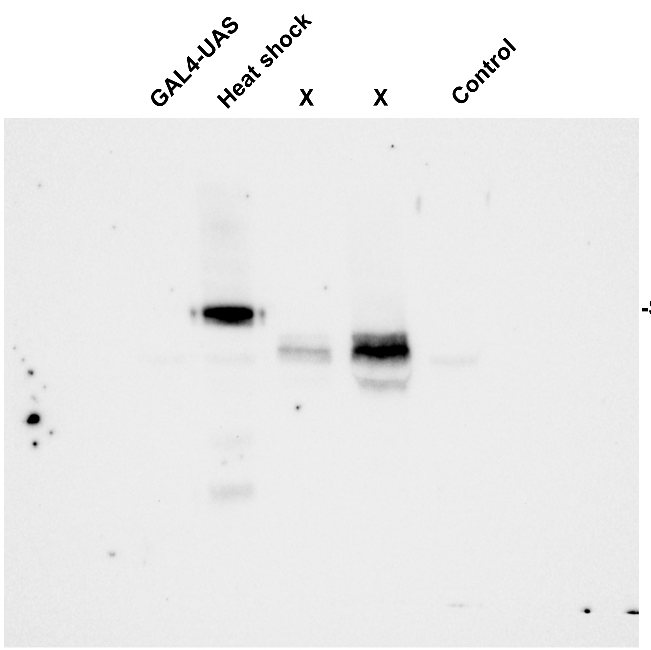

Fig 1B. Comparison of the expression of SCRTT protein from heat-shock and *UAS* promoters (Western Blot)

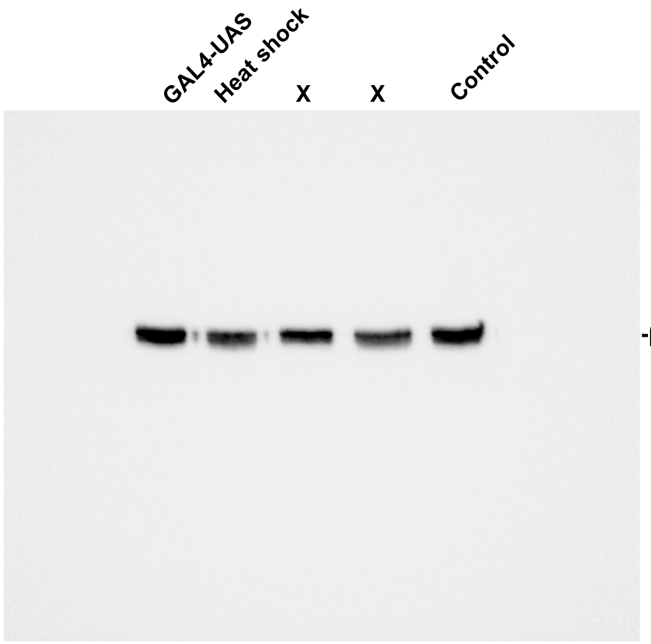

Fig 1B (bottom part). β-tubulin signals as loading control (Western Blot)

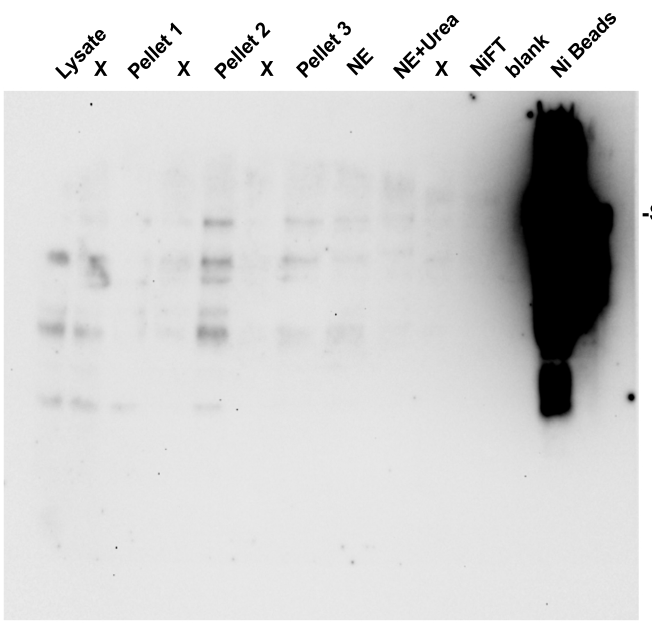

Fig 3B. Overexposed Western Blot showing SCRTT at 62 kDa in the Ni beads fraction

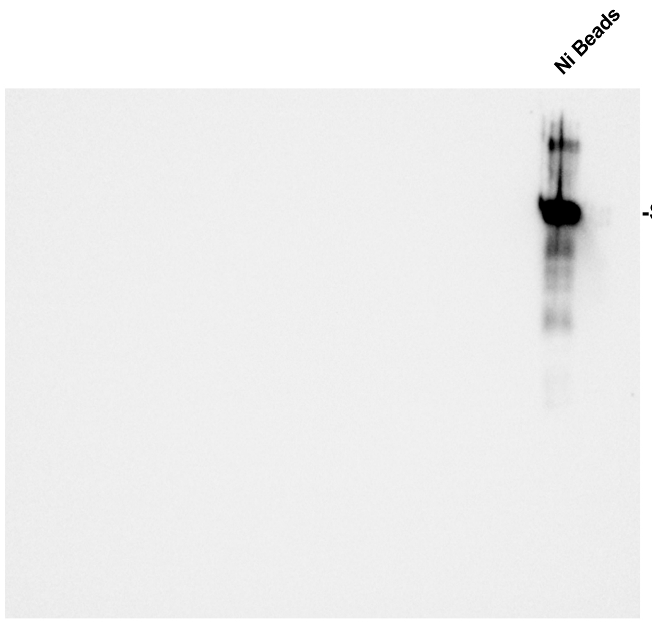

Fig 3C. Autoexposed Western Blot showing SCRTT at 62 kDa in the Ni beads fraction (only the Ni beads fraction has a signal)

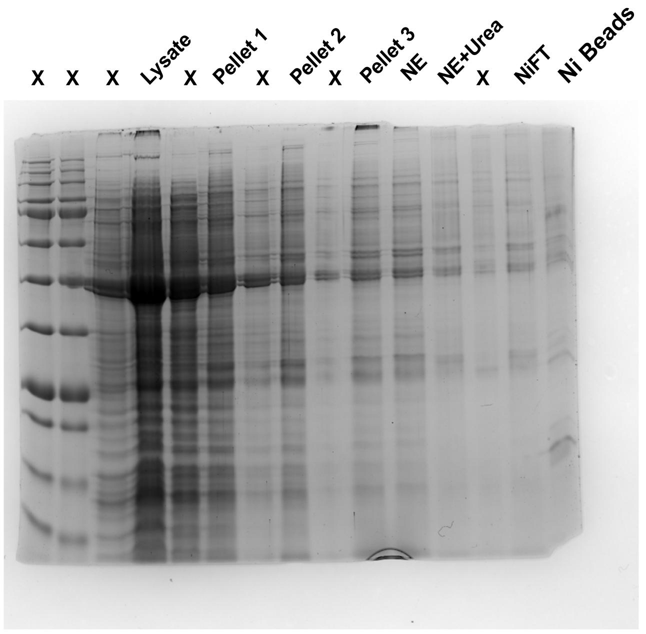

Fig 3D. Coomasie-stained 1-D SDS-polyacrylamide gel of affinity purified SCRTT

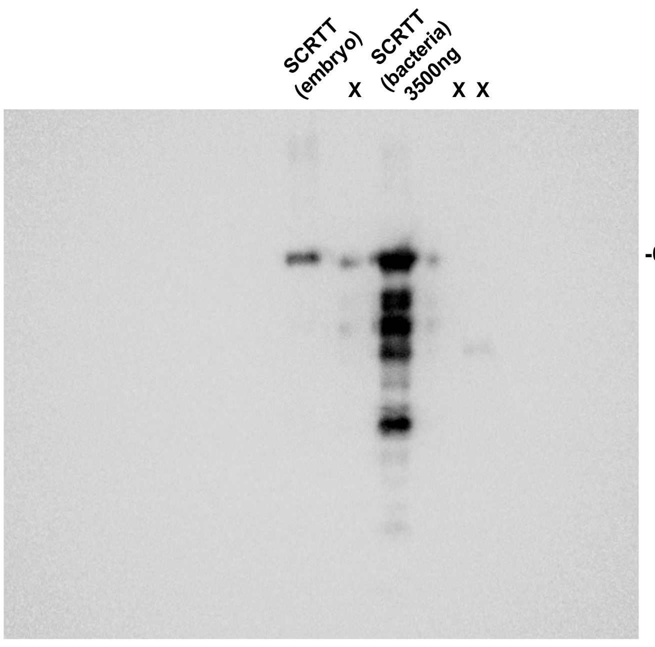

Fig 3E. Comparison of *Drosophila* SCRTT vs. bacterial SCRTT to estimate the amount of protein to be analyzed by MS/MS (Western Blot)
