# Supplementary material for: Post-translational modifications of Drosophila melanogaster HOX protein, Sex combs reduced
Source: PLoS One. 2020 Jan 13;15(1):e0227642. doi: 10.1371/journal.pone.0227642 (PMC6957346; doi:10.1371/journal.pone.0227642)
Supplement: S1 Table — (PDF) [file pone.0227642.s012.pdf]

S1 Table. Post-translational modifications of embryonic SCRTT identified by LC-MS/MS.

Location	Peptide	n*	Protease	Site modified	Modification
12-23	Y.QFVNSLAS(+14.02)C(+57.02)YPQ.Q	1	Chymotrypsin	S19	Methylation
17-24	S.LAS(+14.02)C(+57.02)YPQQ.M	1	Thermolysin	S19	Methylation
17-38	S.LASC(+57.02)YP(+15.99)QQMNPQQNHPGAGNSS.A	1	Thermolysin	P22	Hydroxylation
83-95	Y.TPNLY(+15.99)PNTPQAHY.A	1	Chymotrypsin	Y87	Hydroxylation
86-119	N.LYPNTPQAHYANQAAYGGQGNPD(+15.99)MVDYTQLQPQR.L	1	Trypsin	D108	Hydroxylation
95-119	H.YANQAAYGGQGNP(+15.99)DMVDYTQLQPQR.L	4	Trypsin	P107	Hydroxylation
95-119	H.YANQAAYGGQGNPD(+15.99)MVDYTQLQPQR.L	4	Trypsin	D108	Hydroxylation
95-119	H.YANQAAYGGQGNPD(+43.99)MVDYTQLQPQR.L	1	Trypsin	D108	Carboxylation
96-112	Y.ANQAAYGGQGNP(+15.99)DMVDY.T	1	Chymotrypsin	P107	Hydroxylation
98-119	N.QAAYGGQGNP(+15.99)DMVDYTQLQPQR.L	1	Trypsin	P107	Hydroxylation
98-119	N.QAAYGGQGNPD(+43.99)MVDYTQLQPQR.L	1	Trypsin	D108	Carboxylation
98-119	N.QAAYGGQGNPD(+15.99)MVDYTQLQPQR.L	1	Trypsin	D108	Hydroxylation
99-119	Q.AAYGGQGNPD(+15.99)MVDYTQLQPQR.L	1	Trypsin	D108	Hydroxylation
101-114	A.YGGQGNP(+15.99)DMVDYTQ.L	1	Thermolysin	P107	Hydroxylation
102-112	Y.GGQGNPD(+15.99)MVDY.T	2	Chymotrypsin	D108	Hydroxylation
102-114	Y.GGQGNPD(+15.99)MVDYTQ.L	1	Thermolysin	D108	Hydroxylation
102-115	Y.GGQGNPD(+15.99)MVDYTQL.Q	1	Chymotrypsin	D108	Hydroxylation
102-119	Y.GGQGNPDMVD(+15.99)YTQLQPQR.L	1	Trypsin	D111	Hydroxylation
102-120	Y.GGQGNP(+15.99)DMVDYTQLQPQRL.L	1	Chymotrypsin	P107	Hydroxylation
105-119	Q.GNPDMVD(+15.99)YTQLQPQR.L	1	Trypsin	D111	Hydroxylation
149-169	L.AQQQHPQQQQQQQQANIS(+14.02)C(+57.02)KY.A	1	Chymotrypsin	S166	Methylation

156-169	Q.QQQQQQQANIS(+14.02)C(+57.02)KY.A	1	Chymotrypsin	S166	Methylation
156-169	Q.QQQQQQQANISC(+57.02)K(+14.02)Y.A	1	Chymotrypsin	K168	Methylation
158-169	Q.QQQQQANIS(+14.02)C(+57.02)KY.A	1	Chymotrypsin	S166	Methylation
160-169	Q.QQQANIS(+14.02)C(+57.02)KY.A	1	Chymotrypsin	S166	Methylation
162-169	Q.QANIS(+14.02)C(+57.02)KY.A	1	Chymotrypsin	S166	Methylation
163-169	Q.ANIS(+14.02)C(+57.02)KY.A	2	Chymotrypsin	S166	Methylation
163-169	Q.ANISC(+57.02)K(+14.02)Y.A	1	Chymotrypsin	K168	Methylation
169-213	K.YANDPVTPGGSGGGGVS(+79.97)GSNNNNNSANSNNNNSQSLASPQDLSTR.D	1	Trypsin	S185	Phosphorylation
169-213	K.YANDPVTPGGSGGGGVSGSNNNNNSANSN(+.98)NNNS(+79.97)QSLASPQDLSTR.D	1	Trypsin	S201	Phosphorylation
214-230	R.DISPK(+27.99)LSPSSVVESVAR.S	4	Trypsin	K218	Formylation
214-230	R.DISPK(+42.01)LSPSSVVESVAR.S	2	Trypsin	K218	Acetylation
219-230	K.LSPSS(+42.01)VVESVAR.S	1	Trypsin	S223	Acetylation
219-230	K.LSPSSVVES(+42.01)VAR.S	1	Trypsin	S227	Acetylation
219-231	K.LSPSSVVES(+42.01)VARS.L	1	Trypsin	S227	Acetylation
252-270	N.NNHSGSGVSGGPGNVNVP(+15.99)M.H	1	Chymotrypsin	P269	Hydroxylation
255-270	H.SGSGVSGGPGNVNVP(+15.99)M.H	1	Chymotrypsin	P269	Hydroxylation
259-269	G.VSGGPGN(+.98)VNVP(+15.99).M	1	Thermolysin	P269	Hydroxylation
266-287	N.VNVP(+15.99)MHSPGGGDSDSESDSGNE.A	1	Thermolysin	P269	Hydroxylation
266-294	N.VNVP(+15.99)MHSPGGGDSDSESDSGNEAGSSQNS.G	1	Thermolysin	P269	Hydroxylation
294-307	N.SGNGK(+43.99)KNPPQIYPW.M	1	Chymotrypsin	K298	Carboxylation
300-309	K.NPPQIYP(+15.99)WMK.R	1	Trypsin	P306	Hydroxylation
300-309	K.NPPQIYPW(+43.99)MK.R	1	Trypsin	W307	Carboxylation
300-309	K.NPPQIYPW(+43.99)M(+15.99)K.R	2	Trypsin	W307	Carboxylation
300-309	K.NPPQIYPWMK(+43.99).R	1	Trypsin	K309	Carboxylation
300-309	K.NPPQIYPWM(+15.99)K(+43.99).R	2	Trypsin	K309	Carboxylation
300-310	K.NPPQIYP(+15.99)WMKR.V	1	Trypsin	P306	Hydroxylation
300-310	K.NPPQIYPW(+43.99)MKR.V	2	Trypsin	W307	Carboxylation

300-310	K.NPPQIYPW(+43.99)MK(+42.01)R.V	1	Trypsin	W307<br>K309	Carboxylation<br>Acetylation
300-310	K.NPPQIYPWM(+15.99)K(+27.99)R.V	2	Trypsin	K309	Formylation
300-310	K.NPPQIYPWMK(+27.99)R.V	1	Trypsin	K309	Formylation
300-310	K.NPPQIYPWMK(+43.99)R.V	3	Trypsin	K309	Carboxylation
300-310	K.NPPQIYPWMKR(+15.99).V	1	Trypsin	R310	Hydroxylation
310-326	K.RVHLGTSTVNANGETK(+43.99)R.Q	1	Trypsin	K325	Carboxylation
311-326	R.VHLGT(+79.97)STVN(+.98)ANGETKR.Q	1	Trypsin	T315	Phosphorylation
311-326	R.VHLGTS(+79.97)TVN(+.98)AN(+.98)GETKR.Q	1	Trypsin	S316	Phosphorylation
311-326	R.VHLGTST(+79.97)VNAN(+.98)GETKR.Q	1	Trypsin	T317	Phosphorylation
311-326	R.VHLGTST(+79.97)VN(+.98)AN(+.98)GETKR.Q	1	Trypsin	T317	Phosphorylation
311-326	R.VHLGTSTVNAN(+15.99)GE(+57.02)TKR.Q	1	Trypsin	N321	Hydroxylation
311-326	R.VHLGTSTVNANGE(+43.99)TKR.Q	3	Trypsin	E323	Carboxylation
311-326	R.VHLGTSTVN(+.98)ANGE(+43.99)TKR.Q	1	Trypsin	E323	Carboxylation
311-326	R.V(+57.02)HLGTSTVNANGE(+43.99)TKR.Q	1	Trypsin	E323	Carboxylation
311-326	R.VHLGTSTVNANGE(+43.99)TK(+57.02)R.Q	1	Trypsin	E323	Carboxylation
311-326	R.VHLGTSTVN(+.98)AN(+.98)GET(+79.97)KR.Q	1	Trypsin	T324	Phosphorylation
311-326	R.VHLGTSTVN(+.98)ANGET(+79.97)KR.Q	1	Trypsin	T324	Phosphorylation
311-326	R.VHLGTSTVNAN(+.98)GET(+79.97)KR.Q	2	Trypsin	T324	Phosphorylation
311-326	R.VHLGTSTVNAN(+.98)GETK(+15.99)R.Q	1	Trypsin	K325	Hydroxylation
311-326	R.VHLGTSTVNANGETK(+27.99)R.Q	3	Trypsin	K325	Formylation
311-326	R.VHLGTSTVNAN(+.98)GETK(+27.99)R.Q	3	Trypsin	K325	Formylation
311-326	R.VHLGTSTVN(+.98)ANGETK(+43.99)R.Q	1	Trypsin	K325	Carboxylation
311-326	R.VHLGTSTVNANGE(+57.02)TK(+43.99)R.Q	1	Trypsin	K325	Carboxylation
311-326	R.V(+57.02)HLGTSTVNANGETK(+43.99)R.Q	1	Trypsin	K325	Carboxylation
314-326	L.GTSTVNANGE(+43.99)TKR.Q	1+2^	Trypsin & Chymotrypsin	E323	Carboxylation
314-326	L.GTSTVNANGETK(+43.99)R.Q	1	Chymotrypsin	K325	Carboxylation

329-341	R.TSYTRY(+15.99)QTLELEK.E	1	Trypsin	Y334	Hydroxylation
334-347	R.YQTLELEK(+27.99)EFHFNR.Y	1	Trypsin	K341	Formylation
354-366	R.RIEIAHALC(+57.02)LT(+14.02)ER.Q	1	Trypsin	T364	Methylation
354-366	R.RIEIAHALC(+57.02)LTER(+15.99).Q	1	Trypsin	R366	Hydroxylation
355-366	R.IEIAHALC(+57.02)LT(+14.02)ER.Q	2	Trypsin	T364	Methylation
355-366	R.IEIAHALC(+57.02)LTER(+15.99).Q	1	Trypsin	R366	Hydroxylation
367-375	R.QIK(+27.99)IWFQNR.R	3	Trypsin	K369	Formylation
367-375	R.QIK(+43.99)IWFQNR.R	1	Trypsin	K369	Carboxylation
389-398	M.NIVP(+15.99)YHMGPY.G	1	Chymotrypsin	P392	Hydroxylation
389-398	M.NIVPYHMGPY(+15.99).G	1	Chymotrypsin	Y398	Hydroxylation
431-441	D.IDYK(+42.01)DDDDKEN.L	1	Thermolysin	K434	Acetylation
431-441	D.IDYK(+27.99)DDDDKEN.L	1	Thermolysin	K434	Formylation
431-441	D.IDYKDDDDK(+42.01)EN.L	1	Thermolysin	K439	Acetylation
431-441	D.IDYKDDDDK(+27.99)EN.L	1	Thermolysin	K439	Formylation

* - number of spectrograms in which the peptide was identified

^ - 1 spectrogram for trypsin and 2 for chymotrypsin
