# Supplementary material for: Post-translational modifications of Drosophila melanogaster HOX protein, Sex combs reduced
Source: PLoS One. 2020 Jan 13;15(1):e0227642. doi: 10.1371/journal.pone.0227642 (PMC6957346; doi:10.1371/journal.pone.0227642)
Supplement: S2 Table — (PDF) [file pone.0227642.s013.pdf]

S2 Table. Phosphosites in bovine α-casein identified by MS/MS.

Phosphosites mapped in bovine α-casein	Previous report
S56, S61, S63, S79, S81, S82, S83, S90, S130	Larsen *et al.*, 2005
T64, R134, Y159	-

Phosphosites in green – confidence shown by PEAKS database
