# Supplementary material for: Post-translational modifications of Drosophila melanogaster HOX protein, Sex combs reduced
Source: PLoS One. 2020 Jan 13;15(1):e0227642. doi: 10.1371/journal.pone.0227642 (PMC6957346; doi:10.1371/journal.pone.0227642)
Supplement: S3 Table — (PDF) [file pone.0227642.s014.pdf]

S3 Table. Phosphopeptides of α-casein identified by MS/MS (TiO_2-enriched vs. non-TiO_2).

α-casein phosphopeptides detected without TiO_2 (Total no. of peptides detected = 839; No. of phosphopeptides detected = 158)	α -casein phosphopeptides detected post-TiO_2 (Total no. of peptides detected = 91; No. of phosphopeptides detected = 84)
K.DIGSES(+79.97)TEDQAMEDIK.Q	
K.DIGS(+79.97)ESTEDQAMEDIK.Q	
K.DIGS(+79.97)ES(-18.01)TEDQAMEDIK.Q	
K.DIGS(-18.01)ES(+79.97)TEDQAMEDIK.Q	
K.DIGS(+79.97)ESTEDQAM(+31.99)EDIK.Q	K.DIGS(+79.97)ESTEDQAM(+31.99)EDIK.Q
K.DIGS(+79.97)ESTEDQAM(+15.99)EDIK.Q	K.DIGS(+79.97)ESTEDQAM(+15.99)EDIK.Q
K.Y(+27.99)KVPQLEIVPNS(+79.97)AEER.L	
K.DIGS(+79.97)ES(+79.97)TEDQAMEDIK.Q	K.DIGS(+79.97)ES(+79.97)TEDQAMEDIK.Q
K.DIGS(+79.97)E(+14.02)STEDQAM(+15.99)EDIK.Q	K.DIGS(+79.97)E(+14.02)STEDQAM(+15.99)EDIK .Q
K.D(+57.02)IGSES(+79.97)TEDQAM(+15.99)EDIK.Q	K.D(+57.02)IGSES(+79.97)TEDQAM(+15.99)EDIK .Q
K.DIGSES(+79.97)TEDQAM(+31.99)EDIK.Q	K.DIGSES(+79.97)TEDQAM(+31.99)EDIK.Q
K.DIGSES(+79.97)TEDQAM(+15.99)EDIK.Q	K.DIGSES(+79.97)TEDQAM(+15.99)EDIK.Q
K.DIGS(+79.97)ESTEDQAM(+15.99)EDIK(+57.02).Q	K.DIGS(+79.97)ESTEDQAM(+15.99)EDIK(+57.02) .Q
K.YKVPQLEIVPNS(+79.97)AEE(+14.02)R.L	
K.YKVPQLEIVPN(+.98)S(+79.97)AEER.L	K.YKVPQLEIVPN(+.98)S(+79.97)AEER.L
K.YK(+27.99)VPQLEIVPNS(+79.97)AEER.L	
K.DIGSEST(+79.97)EDQAM(+15.99)EDIK.Q	K.DIGSEST(+79.97)EDQAM(+15.99)EDIK.Q
K.YKVPQLEIVPNS(+79.97)AE(+14.02)ER.L	
K.YK(+42.01)VPQLEIVPNS(+79.97)AEER.L	
K.YKVPQLEIVPNS(+79.97)AEER.L	K.YKVPQLEIVPNS(+79.97)AEER.L
K.D(+57.02)IGS(+79.97)ESTEDQAM(+15.99)EDIK.Q	K.D(+57.02)IGS(+79.97)ESTEDQAM(+15.99)EDIK .Q

K.Y(+57.02)KVPQLEIVPNS(+79.97)AEER.L	K.Y(+57.02)KVPQLEIVPNS(+79.97)AEER.L
K.VPQLEIVPNS(+79.97)AEER.L	K.VPQLEIVPNS(+79.97)AEER.L
K.VPQLEIVPN(+.98)S(+79.97)AEER.L	K.VPQLEIVPN(+.98)S(+79.97)AEER.L
K.VPQLE(+14.02)IVPNS(+79.97)AEER.L	K.VPQLE(+14.02)IVPNS(+79.97)AEER.L
K.DIGS(+79.97)ES(+79.97)TEDQAM(+15.99)EDIK.Q	K.DIGS(+79.97)ES(+79.97)TEDQAM(+15.99)EDIK .Q
K.DIGS(+79.97)EST(+79.97)EDQAM(+15.99)EDIK.Q	K.DIGS(+79.97)EST(+79.97)EDQAM(+15.99)EDIK .Q
K.VPQLEIVPNS(+79.97)AE(+14.02)ER.L	K.VPQLEIVPNS(+79.97)AE(+14.02)ER.L
K.VPQLEIVPNS(+79.97)AE(+57.02)ER.L	K.VPQLEIVPNS(+79.97)AE(+57.02)ER.L
K.DIGSES(+79.97)TEDQ(+.98)AMEDIK.Q	
K.DIGSEST(+79.97)EDQAMEDIK.Q	
K.Y(+43.01)KVPQLEIVPNS(+79.97)AEER.L	
K.YKVPQ(+.98)LEIVPNS(+79.97)AEER.L	
K.VPQLEIVPNS(+79.97)AEE(+14.02)R.L	K.VPQLEIVPNS(+79.97)AEE(+14.02)R.L
K.Y(+57.02)KVPQLEIVPN(+.98)S(+79.97)AEER.L	
K.VPQLE(+21.98)IVPNS(+79.97)AEER.L	K.VPQLE(+21.98)IVPNS(+79.97)AEER.L
K.D(+21.98)IGS(+79.97)ESTEDQAMEDIK.Q	
K.VPQLEIVP(+31.99)NS(+79.97)AEER.L	
K.V(+57.02)PQLEIVPN(+.98)S(+79.97)AEER.L	K.V(+57.02)PQLEIVPN(+.98)S(+79.97)AEER.L
K.YK(+57.02)VPQLEIVPNS(+79.97)AEER.L	K.YK(+57.02)VPQLEIVPNS(+79.97)AEER.L
K.DIGSES(+79.97)T(+79.97)EDQAM(+15.99)EDIK.Q	K.DIGSES(+79.97)T(+79.97)EDQAM(+15.99)EDIK .Q
K.YK(+43.01)VPQLEIVPNS(+79.97)AEER.L	
K.VNELS(+79.97)KDIGSES(+79.97)TEDQAM(+15.99) EDIK.Q	K.VNELS(+79.97)KDIGSES(+79.97)TEDQAM(+15. 99)EDIK.Q
K.V(+27.99)PQLEIVPNS(+79.97)AEER.L	K.V(+27.99)PQLEIVPNS(+79.97)AEER.L
K.V(+57.02)PQLEIVPNS(+79.97)AEER.L	K.V(+57.02)PQLEIVPNS(+79.97)AEER.L

K.VPQLEIVPNS(+79.97)AEE(+57.02)R.L	K.VPQLEIVPNS(+79.97)AEE(+57.02)R.L
K.DIGSES(+79.97)TEDQAM(+15.99)EDIK(+57.02).Q	K.DIGSES(+79.97)TEDQAM(+15.99)EDIK(+57.02).Q
K.YKVPQLE(+57.02)IVPNS(+79.97)AEER.L	K.YKVPQLE(+57.02)IVPNS(+79.97)AEER.L
K.DIGSES(+79.97)TEDQAMEDIK(+43.01).Q	
K.KYKVPQLEIVPNS(+79.97)AEER.L	
K.DIGS(+79.97)ES(+79.97)TEDQAME(+57.02)DIK.Q	
K.DIGS(+79.97)EST(+79.97)EDQAMEDIK.Q	
K.DIGSES(+79.97)TE(+57.02)DQAM(+15.99)EDIK.Q	
K.V(+57.02)PQ(+.98)LEIVPNS(+79.97)AEER.L	K.V(+57.02)PQ(+.98)LEIVPNS(+79.97)AEER.L
K.DIGS(+79.97)E(+57.02)STEDQAM(+15.99)EDIK.Q	
K.YK(+57.02)VPQLEIVPN(+.98)S(+79.97)AEER.L	
K.VNELSKDIGS(+79.97)ES(+79.97)TEDQAM(+15.99)EDIK.Q	K.VNELSKDIGS(+79.97)ES(+79.97)TEDQAM(+15.99)EDIK.Q
D.IGS(+79.97)ES(+79.97)TEDQAM(+15.99)EDIK.Q	D.IGS(+79.97)ES(+79.97)TEDQAM(+15.99)EDIK.Q
G.S(+79.97)ES(+79.97)TEDQAM(+15.99)EDIK.Q	G.S(+79.97)ES(+79.97)TEDQAM(+15.99)EDIK.Q
K.VPQLEIVPNS(+79.97)AEER(-.98).L	K.VPQLEIVPNS(+79.97)AEER(-.98).L
K.VPQLE(+57.02)IVPNS(+79.97)AEER.L	K.VPQLE(+57.02)IVPNS(+79.97)AEER.L
K.YKVPQLE(+21.98)IVPNS(+79.97)AEER.L	
K.Y(+57.02)K(+57.02)VPQLEIVPNS(+79.97)AEER.L	
K.DIGS(+79.97)ES(+79.97)TEDQAMEDIK(+57.02).Q	
K.DIGS(+79.97)ESTEDQAMEDIK(+43.01).Q	
K.VPQLEIVP(+15.99)NS(+79.97)AEER.L	
K.VNELS(+79.97)KDIGS(+79.97)ESTEDQAM(+15.99)EDIK.Q	K.VNELS(+79.97)KDIGS(+79.97)ESTEDQAM(+15.99)EDIK.Q
K.YKVPQLE(+14.02)IVPNS(+79.97)AEER.L	
K.VNELSKDIGS(+79.97)EST(+79.97)EDQAM(+15.99)EDIK.Q	K.VNELSKDIGS(+79.97)EST(+79.97)EDQAM(+15.99)EDIK.Q
K.D(+21.98)IGSES(+79.97)TEDQAMEDIK.Q	

Y.KVPQLEIVPNS(+79.97)AEER.L	
K.DIGSE(+21.98)S(+79.97)TEDQAMEDIK.Q	
K.VPQLEIVPNS(+79.97)AEER(+15.99).L	
K.YK(+57.02)VPQ(+.98)LEIVPNS(+79.97)AEER.L	
R.LK(+43.99)KYKVPQLEIVPNS(+79.97)AEER.L	
K.DIGS(+79.97)EST(+79.97)EDQAME(+57.02)DIK.Q	
K.KYK(+57.02)VPQLEIVPNS(+79.97)AEER.L	
K.VPQLEIVPN(+15.99)S(+79.97)AEER.L	
K.Y(+57.02)KVPQ(+.98)LEIVPNS(+79.97)AEER.L	
K.DIGS(+79.97)ES(+79.97)TE(+57.02)DQAMEDIK.Q	K.DIGS(+79.97)ES(+79.97)TE(+57.02)DQAMEDIK .Q
R.L(+27.99)KKYKVPQLEIVPNS(+79.97)AEER.L	
K.DIGSEST(+79.97)E(+21.98)DQAMEDIK.Q	
K.VNELSK(+57.02)DIGS(+79.97)ES(+79.97)TEDQAM EDIK.Q	
K.YKVPQLEIVPNS(+79.97)A.E	
K.VPQLEIVPNS(+79.97)AE(+57.02)E(+57.02)R.L	
K.YKVPQ(+.98)LE(+14.02)IVPNS(+79.97)AEER.L	
K.DIGSES(+79.97)T(+79.97)EDQAMEDIK.Q	
K.VNELS(+79.97)KDIGS(+79.97)ES(+79.97)TEDQAM (+15.99)EDIK.Q	K.VNELS(+79.97)KDIGS(+79.97)ES(+79.97)TEDQ AM(+15.99)EDIK.Q
K.DIGS(+79.97)ES(+79.97)TEDQAMED(+57.02)IK.Q	
K.YKVPQLEIVPNS(+79.97)AE(+57.02)ER.L	
Q.LE(+14.02)IVPNS(+79.97)AEER.L	Q.LE(+14.02)IVPNS(+79.97)AEER.L
P.QLEIVPNS(+79.97)AEER.L	P.QLEIVPNS(+79.97)AEER.L
K.VPQLEIVPNSAEER(+79.97).L	K.VPQLEIVPNSAEER(+79.97).L
K.Y(+15.99)KVPQLEIVPNS(+79.97)AEER.L	
K.Y(+42.01)KVPQLEIVPNS(+79.97)AEER.L	

K.VNELSKD(+57.02)IGSEST(+79.97)EDQAM(+15.99)EDIK.Q	
K.YKVP(+15.99)QLEIVPNS(+79.97)AEER.L	
K.DIGSES(+79.97)TEDQ(+.98)AM(+15.99)EDIK.Q	K.DIGSES(+79.97)TEDQ(+.98)AM(+15.99)EDIK.Q
S.ES(+79.97)TEDQAM(+15.99)EDIK.Q	S.ES(+79.97)TEDQAM(+15.99)EDIK.Q
K.VPQ(+.98)LE(+14.02)IVPNS(+79.97)AEER.L	
I.GS(+79.97)ES(+79.97)TEDQAM(+15.99)EDIK.Q	I.GS(+79.97)ES(+79.97)TEDQAM(+15.99)EDIK.Q
K.K(+57.02)(+57.02)YKVPQLEIVPNS(+79.97)AEER.L	
K.VPQLEIVPN(+.98)S(+79.97)AE(+57.02)ER.L	
K.Y(+57.02)KVPQLEIVPNS(+79.97)AE(+14.02)ER.L	
L.EIVPNS(+79.97)AEER.L	L.EIVPNS(+79.97)AEER.L
K.VPQLEIVPNS(+79.97)AE(+21.98)ER.L	K.VPQLEIVPNS(+79.97)AE(+21.98)ER.L
K.YKVPQLEIVPN(+.98)S(+79.97)AE(+14.02)ER.L	
K.EKVNELS(+79.97)KDIGSES(+79.97)TEDQAM(+15.99)EDIK.Q	
K.YKVPQLEIVP(+31.99)NS(+79.97)AEER.L	
L.S(+79.97)KDIGS(+79.97)ESTEDQAM(+15.99)EDIK.Q	
K.DIGS(+79.97)ES(+79.97)TED(+57.02)QAMEDIK.Q	
Q.LEIVPNS(+79.97)AEER.L	Q.LEIVPNS(+79.97)AEER.L
Y.K(+57.02)VPQLEIVPNS(+79.97)AEER.L	
R.LK(+27.99)KYKVPQLEIVPNS(+79.97)AEER.L	
L.SKDIGS(+79.97)ES(+79.97)TEDQAM(+15.99)EDIK.Q	
K.YK(+15.99)VPQLEIVPNS(+79.97)AEER.L	
K.YKVPQLEIVPNS(+79.97)AEER(+15.99).L	
K.V(+42.01)PQLEIVPNS(+79.97)AEER.L	
K.V(+57.02)PQLEIVPNS(+79.97)AE(+57.02)ER.L	
K.YKVPQLEIVPNS(+79.97)AE(+21.98)ER.L	

K.YKVPQLE(+57.02)IVPN(+.98)S(+79.97)AEER.L	
I.VPNS(+79.97)AEER.L	
L.EIVPN(+.98)S(+79.97)AEER.L	
K.DIGS(+79.97)ES(+79.97)TEDQN(sub A)MEDIK.Q	
K.YKVPQLEIVPNS(+79.97)AEER(-.98).L	
S.EEIVPNS(+79.97)VEQK.H	S.EEIVPNS(+79.97)VEQK.H
K.QM(+15.99)EAESIS(+79.97)S(+79.97)SEEIVPNSVEQK.H	
K.VNELS(+79.97)KDIGS(+79.97)EST(+79.97)EDQAM(+15.99)EDIK.Q	K.VNELS(+79.97)KDIGS(+79.97)EST(+79.97)EDQAM(+15.99)EDIK.Q
K.EKVNELS(+79.97)KDIGS(+79.97)ESTEDQAM(+15.99)EDIK.Q	
K.YKVPQLE(+14.02)IVPN(+.98)S(+79.97)AEER.L	
K.YKVPQLE(+57.02)IVPNS(+79.97)AEE(+14.02)R.L	
K.E(sub K)YA(sub K)VPQLEIVPNS(+79.97)AEER.L	
K.EKVNELS(+79.97)KDIGS(+79.97)ES(+79.97)TEDQAM(+15.99)EDIK.Q	
K.YKVPQLEIVPNS(+79.97)AE(+57.02)E(+57.02)R.L	
K.Y(+162.05)KVPQLEIVPNS(+79.97)AEER.L	
Q.LEIVPN(+.98)S(+79.97)AEER.L	
K.EKVNELS(+79.97)KDIGS(+79.97)ES(+79.97)TEDQAMEDIK.Q	
K.VP(+15.99)QLEIVPNS(+79.97)AEER.L	
K.V(+42.01)PQLE(+14.02)IVPNS(+79.97)AEER.L	
R.LKKYK(+27.99)VPQLEIVPNS(+79.97)AEER.L	
K.VNELS(+79.97)KDIG.S	
K.DIGSEST(+79.97)EDQAME(+57.02)DIK.Q	
K.QMEAESISSS(+79.97)EE(+14.02)I.V	
K.DIGS(+79.97)ESTE(+21.98)DQAMEDIK.Q	
K.DIGS(+79.97)ESTEDQAME(+57.02)DIK.Q	

Q.LE(+57.02)IVPNS(+79.97)AEER.L	
V.PN(+.98)S(+79.97)AEER.L	
K.EPQ(sub M)IGVNQELAY(+79.97)FYPELFR.Q	
E.EIVPNS(+79.97)VEQK.H	E.EIVPNS(+79.97)VEQK.H
K.DIGS(+79.97)ES(+79.97)TEDQG(sub A)P(sub M)EDIK.Q	
V.PNS(+79.97)AEER.L	V.PNS(+79.97)AEER.L
K.YKVPQLEIVPN(+.98)S(+79.97)AEER(+14.02).L	
K.QMEAESISSS(+79.97)EEI(+14.02).V	
K.VNELS(+79.97)KDIN(sub G)SES(+79.97)TEDQAM(+15.99)EDIK.Q	
K.VNELS(+79.97)K.D	
K.DIGSEST(+79.97)E(+57.02)DQAMEDIK.Q	
K.DIGS(+79.97)ESTEDQAMED(+57.02)IK.Q	
K.DIGS(+79.97)ES(+79.97)TEDQAME(+14.02)DIK.Q	
	K.DIGSEST(+79.97)EDQAM(+15.99)EDIK(+57.02).Q
	K.DIGS(+79.97)ESTED(+57.02)QAM(+15.99)EDIK.Q
	E.S(+79.97)TEDQAM(+15.99)EDIK.Q
	E.ST(+79.97)EDQAM(+15.99)EDIK.Q
	K.VPQLEIVPNS(+79.97)AEER(+14.02).L
	K.VPQ(+.98)LEIVPN(+.98)S(+79.97)AEER.L
	K.DIGS(+79.97)EST(+79.97)EDQAM(+15.99).E
	K.DIGS(+79.97)ES(+79.97)TEDQAM(+15.99).E
	K.DIGS(+79.97)EST(+79.97)EDQ.A
	K.QM(+15.99)EAESISS(+79.97)S(+79.97)EEIVPNSVEQK.H
	K.DIGS(+79.97)ES(+79.97)TEDQAN(sub M)EDIK.Q

	K.DIGS(+79.97)ES(+79.97)TEDQAT(sub M)EDIK.Q
	K.DIGS(+79.97)ES(+79.97)T(+79.97)EDQAM(+15.99)EDIK.Q
	S.S(+79.97)EEIVPNS(+79.97)VEQK.H
	E.IVPNS(+79.97)VEQK.H
	S.EST(+79.97)EDQAM(+15.99)EDIK.Q
	K.DIGS(+79.97)Q(sub E)STEDQAM(+15.99)EDIK.Q
	K.DIGS(+79.97)ES(+79.97)TEDQAM(+15.99)ED.I
	S.SEEIVPNS(+79.97)VEQK.H
	G.S(+79.97)EST(+79.97)EDQAM(+15.99)EDIK.Q
	K.DIGS(+79.97)ES(+79.97)TEDQ.A
	K.DIGS(+79.97)ES(+79.97)TEDQAM(+15.99)E.D
	K.DIGS(+79.97)ESTED(+14.02)Q(+.98)AMEDIK.Q
	K.QM(+15.99)EAES(+79.97)ISSS(+79.97)EEIVPNSVEQK.H
	K.DIGS(+79.97)ESTEDQ.A
	K.DIGSEST(+79.97)EDQAMED(+57.02)IK.Q
	S.S(+79.97)S(+79.97)EEIVPNS(+79.97)VEQK.H
	K.DIGS(+79.97)ES(+79.97)TEDQAL(sub M)EDIK.Q
	K.DIGS(+79.97)ES(+79.97)TEDQC(sub A)MEDIK.Q
	K.DIGSES(+79.97)TEDQ.A
