# Supplementary material for: Post-translational modifications of Drosophila melanogaster HOX protein, Sex combs reduced
Source: PLoS One. 2020 Jan 13;15(1):e0227642. doi: 10.1371/journal.pone.0227642 (PMC6957346; doi:10.1371/journal.pone.0227642)
Supplement: S4 Table — (PDF) [file pone.0227642.s015.pdf]

**S4 Table. SLiMs in *D. melanogaster* SCR**

| Short Linear Motif (SLiM) # | SLiM sequence in SCR | Amino acids | Putative Function |
|---|---|---|---|
| 1 | MDP | 1-3 | N-terminal motif that initiates protein degradation by binding to the UBR-box of N-recognins. This N-degron variant comprises N-terminal Asp or Glu as destabilizing residue. |
| 2 | FAMSSY | 6-11 | NEK2 phosphorylation motif with preferred Phe, Leu or Met in the -3 position to compensate for less favorable residues in the +1 and +2 position. |
| 3 | AMSSY | 7-11 | The USP7 MATH domain binding motif variant based on the MDM2 and p53 interactions. |
| 4 | SSYQF | 9-13 | Phosphopeptide motif which directly interacts with the BRCT (carboxy-terminal) domain of the Breast Cancer Gene BRCA1 with low affinity |
| 5 | SSYQFV | 9-14 | Canonical LIR motif that binds to Atg8 protein family members to mediate processes involved in autophagy. |
| 6 | SYQFV | 10-14 | Canonical LIR motif that binds to Atg8 protein family members to mediate processes involved in autophagy. |
| 7 | YQFV | 11-14 | Tyrosine-based sorting signal responsible for the interaction with mu subunit of AP (Adaptor Protein) complex |
| 8 | YQFVN | 11-15 | CRK family SH2 domain binding motif. |
| 9 | FVNSLA | 13-18 | NEK2 phosphorylation motif with preferred Phe, Leu or Met in the -3 position to compensate for less favorable residues in the +1 and +2 position. |
| 10 | SLASCYP | 16-22 | CK1 phosphorylation site |
| 11 | YPQQ | 21-24 | YXXQ motif found in the cytoplasmic region of cytokine receptors that bind STAT3 SH2 domain. |
| 12 | AGNSS | 34-38 | The USP7 MATH domain binding motif variant based on the MDM2 and p53 interactions. |
| 13 | GNSSAGGS | 35-42 | GSK3 phosphorylation recognition site |
| 14 | AGGSG | 39-43 | The USP7 MATH domain binding motif variant based on the MDM2 and p53 interactions. |
| 15 | AGGSG | 46-50 | The USP7 MATH domain binding motif variant based on the MDM2 and p53 interactions. |
| 16 | SGGTNGG | 55-61 | CK1 phosphorylation site |
| 17 | GAATPG | 66-71 | The Class IV WW domain interaction motif is recognised primarily by the Pin1 phosphorylation-dependent prolyl isomerase. |
| 18 | GAATPGA | 66-72 | Proline-Directed Kinase (e.g. MAPK) phosphorylation site in higher eukaryotes. |
| 19 | YFPA | 75-78 | STAT5 Src Homology 2 (SH2) domain binding motif. |
| 20 | AAYTPN | 80-85 | The Class IV WW domain interaction motif is recognised primarily by the Pin1 phosphorylation-dependent prolyl isomerase. |
| 21 | AAYTPNL | 80-86 | Proline-Directed Kinase (e.g. MAPK) phosphorylation site in higher eukaryotes. |
| 22 | AYTPNLY | 81-87 | Phosphothreonine motif binding a subset of FHA domains that show a preference for a large aliphatic amino acid at the pT+3 position. |

| | | | |
|---|---|---|---|
| 23 | YTPN | 82-85 | STAT5 Src Homology 2 (SH2) domain binding motif. |
| 24 | PNLYP | 84-88 | PxLxP motif is recognized by a subset of MYND domain containing proteins. |
| 25 | NLYPNTP | 85-91 | This is the motif recognized by those SH3 domains with a non-canonical class I recognition specificity. |
| 26 | YPNTPQ | 87-92 | The Class IV WW domain interaction motif is recognised primarily by the Pin1 phosphorylation-dependent prolyl isomerase. |
| 27 | YPNTPQA | 87-93 | Proline-Directed Kinase (e.g. MAPK) phosphorylation site in higher eukaryotes. |
| 28 | PNTPQA | 88-93 | Phospho-dependent motif that mediates docking of CDK substrates and regulators to cyclin-CDK-bound Cks1. |
| 29 | YANQ | 95-98 | GRB2-like Src Homology 2 (SH2) domains binding motif; YXXQ motif found in the cytoplasmic region of cytokine receptors that bind STAT3 SH2 domain. |
| 30 | YGGQ | 101-104 | YXXQ motif found in the cytoplasmic region of cytokine receptors that bind STAT3 SH2 domain. |
| 31 | VDYTQLQ | 110-116 | (ST)Q motif which is phosphorylated by PIKK family members; Ser/Thr residue phosphorylated by the Plk1 kinase |
| 32 | DYTQL | 111-115 | Canonical LIR motif that binds to Atg8 protein family members to mediate processes involved in autophagy. |
| 33 | YTQL | 112-115 | STAT5 Src Homology 2 (SH2) domain binding motif; Tyrosine-based sorting signal responsible for the interaction with mu subunit of AP (Adaptor Protein) complex |
| 34 | YTQLQ | 112-116 | CRK family SH2 domain binding motif. |
| 35 | ANISCK | 163-168 | NEK2 phosphorylation motif with specific set of residues in the +1 and +2 position to compensate for less favorable residues in the -3 position. |
| 36 | YAND | 169-172 | GRB2-like Src Homology 2 (SH2) domains binding motif. |
| 37 | ANDPVTP | 170-176 | This is the motif recognized by those SH3 domains with a non-canonical class I recognition specificity. |
| 38 | DPVTPG | 172-177 | The Class IV WW domain interaction motif is recognised primarily by the Pin1 phosphorylation-dependent prolyl isomerase. |
| 39 | DPVTPGG | 172-178 | Proline-Directed Kinase (e.g. MAPK) phosphorylation site in higher eukaryotes. |
| 40 | DPVTPGGS | 172-179 | GSK3 phosphorylation recognition site |
| 41 | PVTPGG | 173-178 | Phospho-dependent motif that mediates docking of CDK substrates and regulators to cyclin-CDK-bound Cks1. |
| 42 | PVTPGGS | 173-179 | The TPxxS phospho-dependent degron binds the FBW7 F box proteins of the SCF (Skp1_Cullin-Fbox) complex. |
| 43 | VTPGGS | 174-179 | The TPxxS phospho-dependent degron binds the FBW7 F box proteins of the SCF (Skp1_Cullin-Fbox) complex. |
| 44 | PGGSG | 176-180 | The USP7 MATH domain binding motif variant based on the MDM2 and p53 interactions. |
| 45 | SANSNNN | 193-199 | CK1 phosphorylation site |
| 46 | NNNSQSL | 198-204 | (ST)Q motif which is phosphorylated by PIKK family members. |

| | | | |
|---|---|---|---|
| 47 | SLASPQ | 203-208 | The Class IV WW domain interaction motif is recognised primarily by the Pin1 phosphorylation-dependent prolyl isomerase. |
| 48 | SLASPQD | 203-209 | CK1 phosphorylation site; Proline-Directed Kinase (e.g. MAPK) phosphorylation site in higher eukaryotes |
| 49 | DLSTRDIS | 209-216 | GSK3 phosphorylation recognition site |
| 50 | LSTRDIS | 210-216 | Phosphothreonine motif binding a subset of FHA domains that show a preference for a large aliphatic amino acid at the pT+3 position. |
| 51 | RDISPK | 213-218 | The Class IV WW domain interaction motif is recognised primarily by the Pin1 phosphorylation-dependent prolyl isomerase; Short version of the CDK phosphorylation site which shows specificity towards a lysine/arginine residue at the [ST] +2 position. |
| 52 | RDISPKL | 213-219 | Proline-Directed Kinase (e.g. MAPK) phosphorylation site in higher eukaryotes. |
| 53 | RDISPKLS | 213-220 | GSK3 phosphorylation recognition site |
| 54 | PKLSP | 217-221 | PxLxP motif is recognized by a subset of MYND domain containing proteins. |
| 55 | PKLSPS | 217-222 | The Class IV WW domain interaction motif is recognised primarily by the Pin1 phosphorylation-dependent prolyl isomerase. |
| 56 | PKLSPSS | 217-223 | Proline-Directed Kinase (e.g. MAPK) phosphorylation site in higher eukaryotes. |
| 57 | SPSSVVE | 220-226 | CK1 phosphorylation site; Casein kinase 2 (CK2) phosphorylation site; Ser/Thr residue phosphorylated by Plk4 |
| 58 | SPSSVVES | 220-227 | GSK3 phosphorylation recognition site |
| 59 | VVESVARS | 224-231 | GSK3 phosphorylation recognition site |
| 60 | LGGSLA | 237-242 | NEK2 phosphorylation motif with preferred Phe, Leu or Met in the -3 position to compensate for less favorable residues in the +1 and +2 position. |
| 61 | SGVSGGP | 257-263 | CK1 phosphorylation site |
| 62 | PGNVNVP | 263-269 | This is the motif recognized by those SH3 domains with a non-canonical class I recognition specificity. |
| 63 | PMHSPG | 269-274 | The Class IV WW domain interaction motif is recognised primarily by the Pin1 phosphorylation-dependent prolyl isomerase. |
| 64 | PMHSPGG | 269-275 | Proline-Directed Kinase (e.g. MAPK) phosphorylation site in higher eukaryotes. |
| 65 | GGDSDSE | 275-281 | Casein kinase 2 (CK2) phosphorylation site |
| 66 | GGDSDSES | 275-282 | GSK3 phosphorylation recognition site |
| 67 | DSDSESDS | 277-284 | GSK3 phosphorylation recognition site |
| 68 | SESDS | 280-284 | Caspase-3 and Caspase-7 cleavage site. |
| 69 | ESDSGNE | 281-287 | Casein kinase 2 (CK2) phosphorylation site |
| 70 | EAGSSQNS | 287-294 | GSK3 phosphorylation recognition site |
| 71 | AGSSQ | 288-292 | The USP7 MATH domain binding motif variant based on the MDM2 and p53 interactions. |
| 72 | AGSSQNS | 288-294 | (ST)Q motif which is phosphorylated by PIKK family members. |
| 73 | SQNSGNG | 291-297 | CK1 phosphorylation site |

| | | | |
|---|---|---|---|
| 74 | YPWM | 305-308 | The YPWM motif confers binding to the PBX homeobox domain; Tyrosine-based sorting signal responsible for the interaction with mu subunit of AP (Adaptor Protein) complex |
| 75 | KRVHLGTSTVNANGET KRQRT | 309-329 | Bipartite variant of the classical basically charged NLS. |
| 76 | LGTSTV | 313-318 | NEK2 phosphorylation motif with preferred Phe, Leu or Met in the -3 position to compensate for less favorable residues in the +1 and +2 position. |
| 77 | LGTSTVN | 313-319 | Phosphothreonine motif binding a subset of FHA domains that show a preference for a large aliphatic amino acid at the pT+3 position. |
| 78 | YHQF | 402-405 | Tyrosine-based sorting signal responsible for the interaction with mu subunit of AP (Adaptor Protein) complex |
| 79 | IHPSQFA | 407-413 | (ST)Q motif which is phosphorylated by PIKK family members. |
| 80 | SQFAHL | 410-415 | Canonical LIR motif that binds to Atg8 protein family members to mediate processes involved in autophagy. |
| 81 | FAHLSA | 412-417 | The C-terminal class 2 PDZ-binding motif is classically represented by a pattern such as (VYF)X(VIL)* |

Dark red – Amino acid residues that are phosphorylated
Green – Amino acid residues that are hydroxylated
Yellowish green – Amino acid residues that are methylated
Grey – Amino acid residues that are carboxylated
Light blue – Amino acid residues that are acetylated
Purple – Amino acid residues that are acetylated or formylated
Pink – Amino acid residues that are carboxylated or acetylated or formylated
Orange – Amino acid residues that are formylated or carboxylated or hydroxylated

SLiMs underlined are candidate phosphorylation sites.
