# Supplementary material for: Post-translational modifications of Drosophila melanogaster HOX protein, Sex combs reduced
Source: PLoS One. 2020 Jan 13;15(1):e0227642. doi: 10.1371/journal.pone.0227642 (PMC6957346; doi:10.1371/journal.pone.0227642)
Supplement: S5 Table — (PDF) [file pone.0227642.s016.pdf]

S5 Table. Conserved SLiMs in *D. melanogaster* SCR

Short Linear Motif (SLiM) #	SLiM sequence in SCR	Amino acids	Taxonomic level of conservation	Putative Function
2	FAMSSY	6-11	*Drosophila*	NEK2 phosphorylation motif with preferred Phe, Leu or Met in the -3 position to compensate for less favorable residues in the +1 and +2 position.
3	AMSSY	7-11	*Drosophila*	The USP7 MATH domain binding motif variant based on the MDM2 and p53 interactions.
4	SSYQF	9-13	Arthropoda	Phosphopeptide motif which directly interacts with the BRCT (carboxy-terminal) domain of the Breast Cancer Gene BRCA1 with low affinity
5	SSYQFV	9-14	Arthropoda	Canonical LIR motif that binds to Atg8 protein family members to mediate processes involved in autophagy.
6	SYQFV	10-14	Arthropoda	Canonical LIR motif that binds to Atg8 protein family members to mediate processes involved in autophagy.
7	YQFV	11-14	Arthropoda	Tyrosine-based sorting signal responsible for the interaction with mu subunit of AP (Adaptor Protein) complex
8	YQFVN	11-15	Arthropoda	CRK family SH2 domain binding motif.
9	FVNSLA	13-18	Arthropoda	NEK2 phosphorylation motif with preferred Phe, Leu or Met in the -3 position to compensate for less favorable residues in the +1 and +2 position.
10	SLA**S**CY**P**	16-22	*Drosophila*	CK1 phosphorylation site
17	GAATPG	66-71	*Drosophila*	The Class IV WW domain interaction motif is recognised primarily by the Pin1 phosphorylation-dependent prolyl isomerase.
18	GAATPGA	66-72	*Drosophila*	Proline-Directed Kinase (e.g. MAPK) phosphorylation site in higher eukaryotes.
20	AAYTPN	80-85	*Drosophila*	The Class IV WW domain interaction motif is recognised primarily by the Pin1 phosphorylation-dependent prolyl isomerase.
21	AAYTPNL	80-86	*Drosophila*	Proline-Directed Kinase (e.g. MAPK) phosphorylation site in higher eukaryotes.
22	AYTPNL**Y**	81-87	*Drosophila*	Phosphothreonine motif binding a subset of FHA domains that show a preference for a large aliphatic amino acid at the pT+3 position.
24	PNL**Y**P	84-88	*Drosophila*	PxLxP motif is recognized by a subset of MYND domain containing proteins.
25	NL**Y**PNTP	85-91	*Drosophila*	This is the motif recognized by those SH3 domains with a non-canonical class I recognition specificity.
26	**Y**PNTPQ	87-92	*Drosophila*	The Class IV WW domain interaction motif is recognised primarily by the Pin1 phosphorylation-dependent prolyl isomerase.
27	**Y**PNTPQA	87-93	*Drosophila*	Proline-Directed Kinase (e.g. MAPK) phosphorylation site in higher eukaryotes.
28	PNTPQA	88-93	*Drosophila*	Phospho-dependent motif that mediates docking of CDK substrates and regulators to cyclin-CDK-bound Cks1.

31	VDYTQLQ	110-116	Diptera	(ST)Q motif which is phosphorylated by PIKK family members.
32	DYTQL	111-115	Insecta	Canonical LIR motif that binds to Atg8 protein family members to mediate processes involved in autophagy.
33	YTQL	112-115	Insecta	STAT5 Src Homology 2 (SH2) domain binding motif; Tyrosine-based sorting signal responsible for the interaction with mu subunit of AP (Adaptor Protein) complex
34	YTQLQ	112-116	Insecta	CRK family SH2 domain binding motif.
44	PGGSG	176-180	*Drosophila*	The USP7 MATH domain binding motif variant based on the MDM2 and p53 interactions.
47	SLASPQ	203-208	*Drosophila*	The Class IV WW domain interaction motif is recognised primarily by the Pin1 phosphorylation-dependent prolyl isomerase.
48	SLASPQD	203-209	*Drosophila*	CK1 phosphorylation site; Proline-Directed Kinase (e.g. MAPK) phosphorylation site in higher eukaryotes
49	DLSTRDIS	209-216	*Drosophila*	GSK3 phosphorylation recognition site
50	LSTRDIS	210-216	*Drosophila*	Phosphothreonine motif binding a subset of FHA domains that show a preference for a large aliphatic amino acid at the pT+3 position.
51	RDISPK	213-218	*Drosophila*	The Class IV WW domain interaction motif is recognised primarily by the Pin1 phosphorylation-dependent prolyl isomerase; Short version of the CDK phosphorylation site which shows specificity towards a lysine/arginine residue at the [ST] +2 position.
52	RDISPKL	213-219	*Drosophila*	Proline-Directed Kinase (e.g. MAPK) phosphorylation site in higher eukaryotes.
53	RDISPKLS	213-220	*Drosophila*	GSK3 phosphorylation recognition site
54	PKLSP	217-221	*Drosophila*	PxLxP motif is recognized by a subset of MYND domain containing proteins.
55	PKLSPS	217-222	*Drosophila*	The Class IV WW domain interaction motif is recognised primarily by the Pin1 phosphorylation-dependent prolyl isomerase.
56	PKLSPSS	217-223	*Drosophila*	Proline-Directed Kinase (e.g. MAPK) phosphorylation site in higher eukaryotes.
57	SPSSVVE	220-226	*Drosophila*	CK1 phosphorylation site; CK2 phosphorylation site
58	SPSSVVES	220-227	*Drosophila*	GSK3 phosphorylation recognition site
59	VVESVARS	224-231	*Drosophila*	GSK3 phosphorylation recognition site
60	LGGSLA	237-242	*Drosophila*	NEK2 phosphorylation motif with preferred Phe, Leu or Met in the -3 position to compensate for less favorable residues in the +1 and +2 position.
63	PMHSPG	269-274	*Drosophila*	The Class IV WW domain interaction motif is recognised primarily by the Pin1 phosphorylation-dependent prolyl isomerase.
64	PMHSPGG	269-275	*Drosophila*	Proline-Directed Kinase (e.g. MAPK) phosphorylation site in higher eukaryotes.
65	GGDSDSE	275-281	*Drosophila*	CK2 phosphorylation site
66	GGDSDSES	275-282	*Drosophila*	GSK3 phosphorylation recognition site

67	DSDSESDS	277-284	*Drosophila*	GSK3 phosphorylation recognition site
68	SESDS	280-284	*Drosophila*	Caspase-3 and Caspase-7 cleavage site.
69	ESDSGNE	281-287	*Drosophila*	CK2 phosphorylation site
70	EAGSSQNS	287-294	*Drosophila*	GSK3 phosphorylation recognition site
71	AGSSQ	288-292	*Drosophila*	The USP7 MATH domain binding motif variant based on the MDM2 and p53 interactions.
72	AGSSQNS	288-294	*Drosophila*	(ST)Q motif which is phosphorylated by PIKK family members.
73	SQNSGNG	291-297	*Drosophila*	CK1 phosphorylation site
74	Y**P**W**M**	305-308	Bilateria	The YPWM motif confers binding to the PBX homeobox domain; Tyrosine-based sorting signal responsible for the interaction with mu subunit of AP (Adaptor Protein) complex
79	IHPSQFA	407-413	Diptera	(ST)Q motif which is phosphorylated by PIKK family members.
80	SQFAHL	410-415	Insecta	Canonical LIR motif that binds to Atg8 protein family members to mediate processes involved in autophagy.
81	FAHLSA	412-417	Insecta	The C-terminal class 2 PDZ-binding motif is classically represented by a pattern such as (VYF)X(VIL)*

Dark red – Amino acid residues that are phosphorylated
Green – Amino acid residues that are hydroxylated
Yellowish green – Amino acid residues that are methylated
Grey – Amino acid residues that are carboxylated
Light blue – Amino acid residues that are acetylated

SLiMs underlined are candidate phosphorylation sites.
