# Supplementary material for: Post-translational modifications of Drosophila melanogaster HOX protein, Sex combs reduced
Source: PLoS One. 2020 Jan 13;15(1):e0227642. doi: 10.1371/journal.pone.0227642 (PMC6957346; doi:10.1371/journal.pone.0227642)
Supplement: S6 Table — (PDF) [file pone.0227642.s017.pdf]

S6 Table. Accession numbers of animal SCR protein homolog sequences (retrieved from NCBI).

**Organism**	**Taxonomic group**	**SCR**
Drosophila melanogaster	Diptera	NP_524248.2
**Organism**	**Taxonomic group**	***Drosophila melanogaster* HOX homologs**
Drosophila pseudoobscura	Diptera	XP_001359213.3
Drosophila virilis	Diptera	AAO01077.1 (partial) + XP_015027472.1
Anopheles gambiae	Diptera	AAC31944.1
Bombyx mori	Lepidoptera	BAA76868.1
Tribolium castaneum	Coleoptera	AAK16422.1
Apis mellifera	Hymenoptera	XP_623903.2
Daphnia magna	Crustacea	BAJ05331.1
*Tetranychus urticae**	Chelicarata	tetur20g02540
Euperipatoides kanangrensis	Onychophora	CCK73373.1
Capitella teleta	Annelida	ABY67956.1
Gibbula varia	Mollusca	ADJ18234.1 (Hox5)
Antalis entalis	Mollusca	APD15655.1 (Hox5)
Novocrania anomala	Brachiopoda	ANO46567.1
Peronella japonica	Echinodermata	BAO57698.1 (Hox5)
Saccogloccus kowalevskii	Hemichordata	ABK00019.1 (Hox5)
Branchiostoma lanceolatum	Cephalochordata/Amphioxus	ACJ74385.1 (Hox5)
Ciona intestinalis	Urochordata/Tunicata	NP_001027665.1 (Hox5)

* *Tetranychus urticae* HOX protein sequences were retrieved from the *Tetranychus urticae* database in Online Resource for Community Annotation of Eukaryotes (ORCAE) - http://bioinformatics.psb.ugent.be/orcae/overview/Tetur
