# Supplementary material for: Post-translational modifications of Drosophila melanogaster HOX protein, Sex combs reduced
Source: PLoS One. 2020 Jan 13;15(1):e0227642. doi: 10.1371/journal.pone.0227642 (PMC6957346; doi:10.1371/journal.pone.0227642)
Supplement: S1 Fig — Each blue line underneath the primary protein sequence of SCRTT represents a chemically distinct peptide identified by MS/MS analysis. The modifications are indicated by letters or symbols on the blue lines. The C-terminal triple tag sequence is represented by the faded region. On the right is the legend for all modifications shown in the figure. Amino acid substitutions have been excluded from the figure. (A) Analysis of tryptic peptides. A1, A2, A3 and A4 are independent MS/MS analyses. (B) Analysis of chymotryptic peptides. A1 and A2 are independent MS/MS analyses. (C) Analysis of thermolytic peptides. A1 and A2 are independent MS/MS analyses. (PDF) [file pone.0227642.s001.pdf]

A

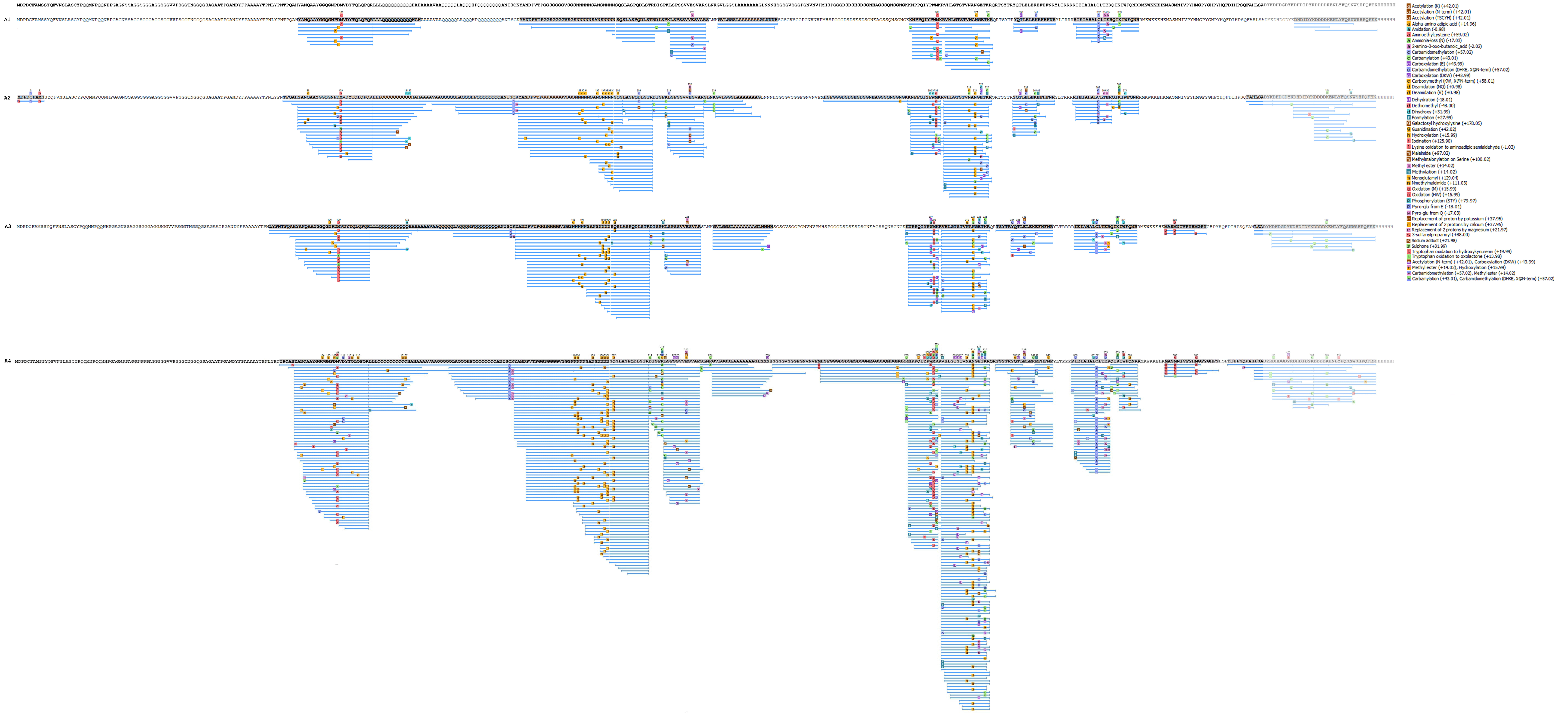
A1
A2
A3
A4
Acetylation (K) (+42.01)
Acetylation (N-term) (+42.01)
Acetylation (TSCYH) (+42.01)
Alpha-amino adipic acid (+14.96)
Amidation (-0.98)
Aminoethylcysteine (+59.02)
Ammonia-loss (N) (-17.03)
2-amino-3-oxo-butanoic_acid (-2.02)
Carbamidomethylation (+57.02)
Carbamylation (+43.01)
Carboxylation (E) (+43.99)
Carbamidomethylation (DHKE, X@N-term) (+57.02)
Carboxylation (DKW) (+43.99)
Carboxymethyl (KW, X@N-term) (+58.01)
Deamidation (NQ) (+0.98)
Deamidation (R) (+0.98)
Dehydration (-18.01)
Dethiomethyl (-48.00)
Dihydroxy (+31.99)
Formylation (+27.99)
Galactosyl hydroxylysine (+178.05)
Guanidination (+42.02)
Hydroxylation (+15.99)
Iodination (+125.90)
Lysine oxidation to aminoadipic semialdehyde (-1.03)
Maleimide (+97.02)
Methylmalonylation on Serine (+100.02)
Methyl ester (+14.02)
Methylation (+14.02)
Monoglutamyl (+129.04)
Nmethylmaleimide (+111.03)
Oxidation (M) (+15.99)
Oxidation (HW) (+15.99)
Phosphorylation (STY) (+79.97)
Pyro-glu from E (-18.01)
Pyro-glu from Q (-17.03)
Replacement of proton by potassium (+37.96)
Replacement of 2 protons by calcium (+37.95)
Replacement of 2 protons by magnesium (+21.97)
3-sulfanylpropanoyl (+88.00)
Sodium adduct (+21.98)
Sulphone (+31.99)
Tryptophan oxidation to hydroxykynurenin (+19.99)
Tryptophan oxidation to oxolactone (+13.98)
Acetylation (N-term) (+42.01), Carboxylation (DKW) (+43.99)
Methyl ester (+14.02), Hydroxylation (+15.99)
Carbamidomethylation (+57.02), Methyl ester (+14.02)
Carbamylation (+43.01), Carbamidomethylation (DHKE, X@N-term) (+57.02)

B

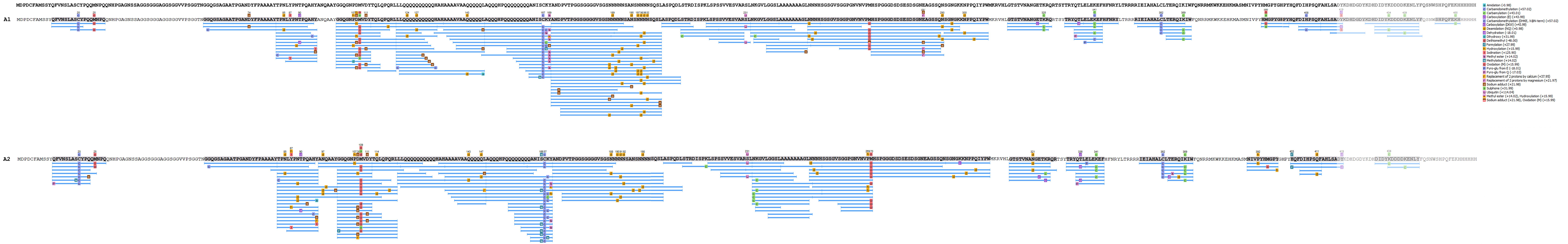

A1
A2
Amidation (-0.98)
Carbamidomethylation (+57.02)
Carbamylation (+43.01)
Carboxylation (E) (+43.99)
Carbamidomethylation (DHKE, X@N-term) (+57.02)
Carboxylation (DKW) (+43.99)
Deamidation (NQ) (+0.98)
Dehydration (-18.01)
Dihydroxy (+31.99)
Dethiomethyl (-48.00)
Formylation (+27.99)
Hydroxylation (+15.99)
Iodination (+125.90)
Methyl ester (+14.02)
Methylation (+14.02)
Oxidation (M) (+15.99)
Pyro-glu from E (-18.01)
Pyro-glu from Q (-17.03)
Replacement of 2 protons by calcium (+37.95)
Replacement of 2 protons by magnesium (+21.97)
Sodium adduct (+21.98)
Sulphone (+31.99)
Ubiquitin (+114.04)
Methyl ester (+14.02), Hydroxylation (+15.99)
Sodium adduct (+21.98), Oxidation (M) (+15.99)

C

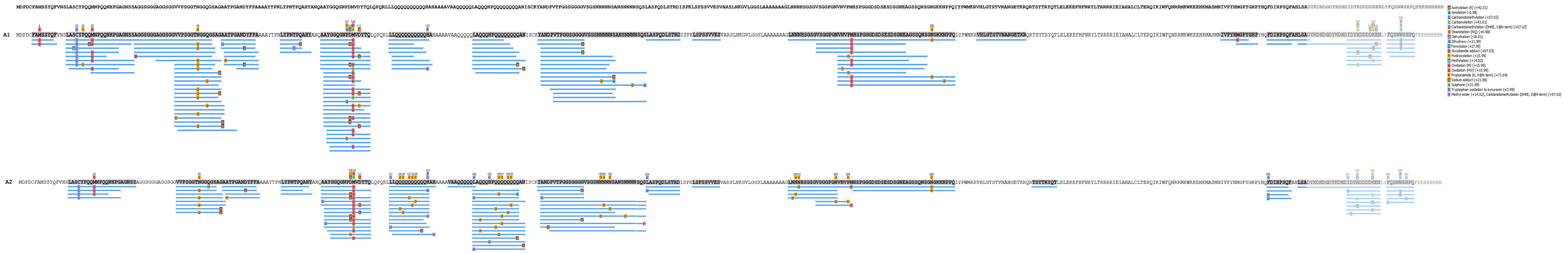

A1
A2
Acetylation (K) (+42.01)
Amidation (-0.98)
Carbamidomethylation (+57.02)
Carbamylation (+43.01)
Carbamidomethylation (DHKE, X@N-term) (+57.02)
Deamidation (NQ) (+0.98)
Dehydration (-18.01)
Dihydroxy (+31.99)
Formylation (+27.99)
Glycidamide adduct (+87.03)
Hydroxylation (+15.99)
Methylation (+14.02)
Oxidation (M) (+15.99)
Oxidation (HW) (+15.99)
Propionamide (K, X@N-term) (+71.04)
Sodium adduct (+21.98)
Sulphone (+31.99)
Tryptophan oxidation to kynurenin (+3.99)
Methyl ester (+14.02), Carbamidomethylation (DHKE, X@N-term) (+57.02)
