# Supplementary material for: Post-translational modifications of Drosophila melanogaster HOX protein, Sex combs reduced
Source: PLoS One. 2020 Jan 13;15(1):e0227642. doi: 10.1371/journal.pone.0227642 (PMC6957346; doi:10.1371/journal.pone.0227642)
Supplement: S2 Fig — Each blue line underneath the primary protein sequence of α-casein represents a chemically distinct tryptic peptide identified by MS/MS analysis. The modifications are indicated by letters or symbols on the blue lines. On the right is the legend for all modifications shown in the figure. Amino acid substitutions have been excluded from the figure. (A) Analysis of α-casein without TiO2 treatment. (B) Analysis of α-casein with TiO2 treatment. (PDF) [file pone.0227642.s002.pdf]

A

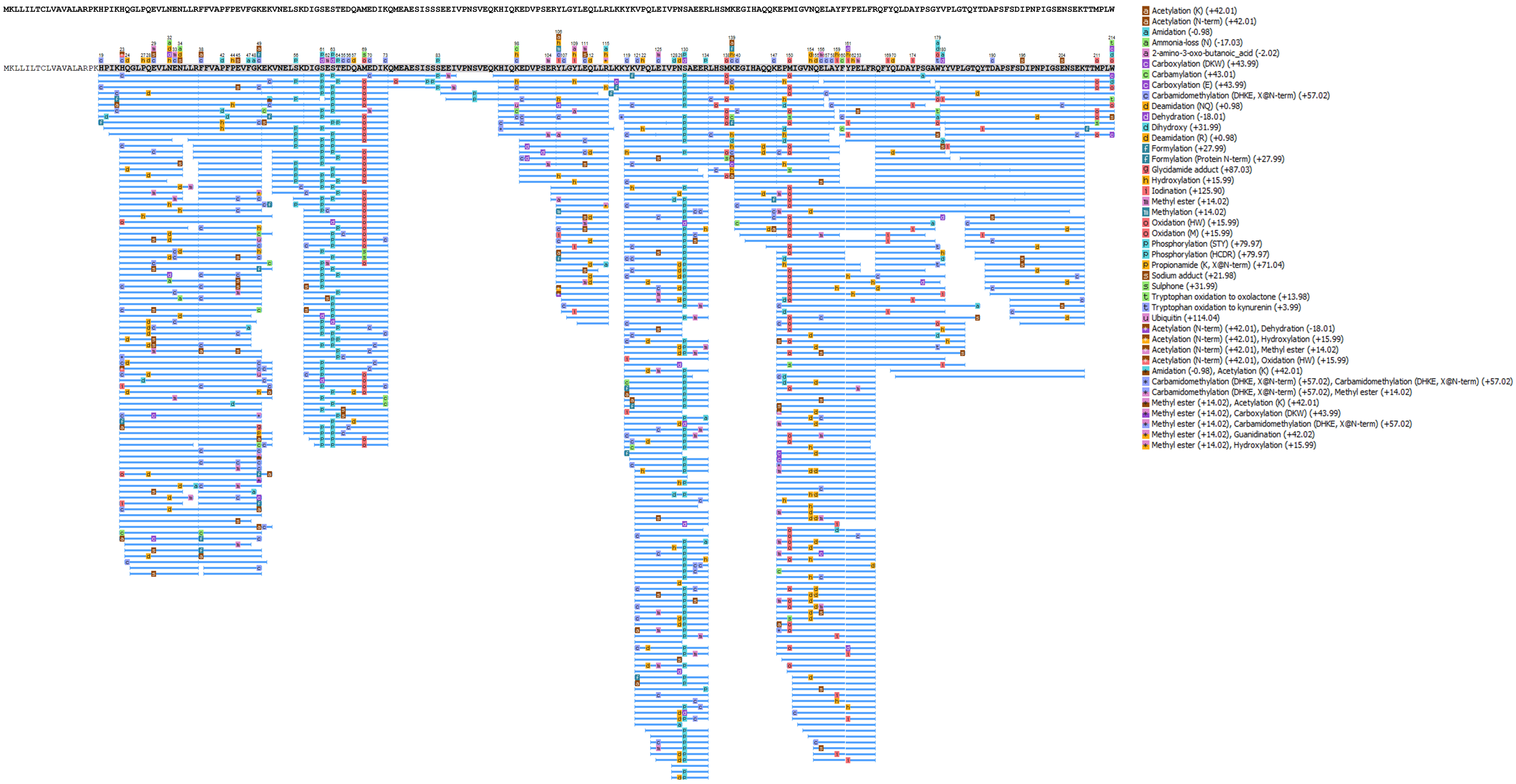
a Acetylation (K) (+42.01)
a Acetylation (N-term) (+42.01)
a Amidation (-0.98)
a Ammonia-loss (N) (-17.03)
a 2-amino-3-oxo-butanoic_acid (-2.02)
c Carboxylation (DKW) (+43.99)
c Carbamylation (+43.01)
c Carboxylation (E) (+43.99)
c Carbamidomethylation (DHKE, X@N-term) (+57.02)
d Deamidation (NQ) (+0.98)
d Dehydration (-18.01)
d Dihydroxy (+31.99)
d Deamidation (R) (+0.98)
f Formylation (+27.99)
f Formylation (Protein N-term) (+27.99)
g Glycidamide adduct (+87.03)
h Hydroxylation (+15.99)
i Iodination (+125.90)
m Methyl ester (+14.02)
m Methylation (+14.02)
o Oxidation (HW) (+15.99)
o Oxidation (M) (+15.99)
p Phosphorylation (STY) (+79.97)
p Phosphorylation (HCDR) (+79.97)
p Propionamide (K, X@N-term) (+71.04)
s Sodium adduct (+21.98)
s Sulphone (+31.99)
t Tryptophan oxidation to oxolactone (+13.98)
t Tryptophan oxidation to kynurenin (+3.99)
u Ubiquitin (+114.04)
* Acetylation (N-term) (+42.01), Dehydration (-18.01)
* Acetylation (N-term) (+42.01), Hydroxylation (+15.99)
* Acetylation (N-term) (+42.01), Methyl ester (+14.02)
* Acetylation (N-term) (+42.01), Oxidation (HW) (+15.99)
* Amidation (-0.98), Acetylation (K) (+42.01)
* Carbamidomethylation (DHKE, X@N-term) (+57.02), Carbamidomethylation (DHKE, X@N-term) (+57.02)
* Carbamidomethylation (DHKE, X@N-term) (+57.02), Methyl ester (+14.02)
* Methyl ester (+14.02), Acetylation (K) (+42.01)
* Methyl ester (+14.02), Carboxylation (DKW) (+43.99)
* Methyl ester (+14.02), Carbamidomethylation (DHKE, X@N-term) (+57.02)
* Methyl ester (+14.02), Guanidination (+42.02)
* Methyl ester (+14.02), Hydroxylation (+15.99)

B

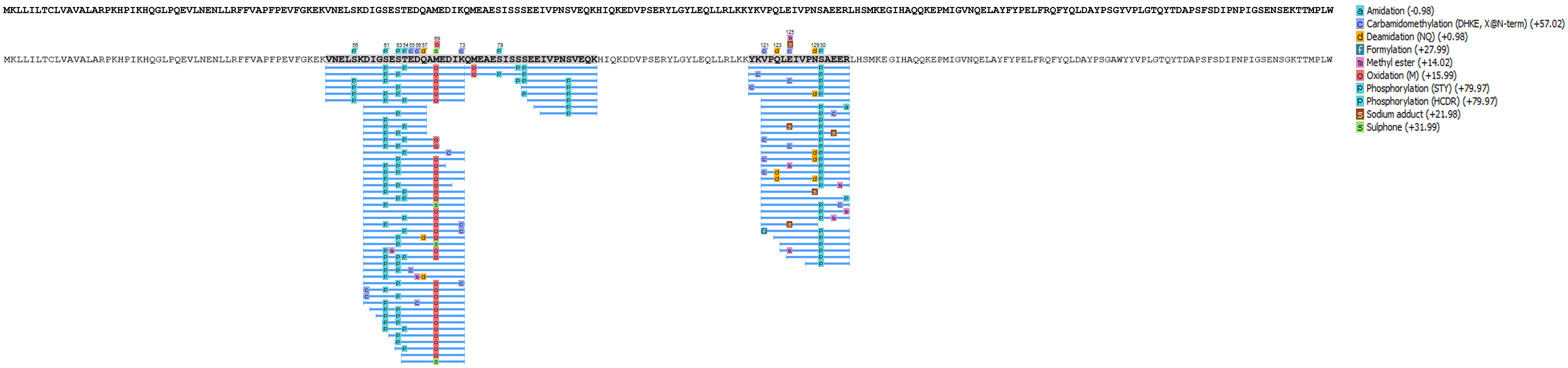

MKLLILTCLVAVALARPKHPIKHQGLPQEVLNENLLRFFVAPFPEVFGKEKVNELSKDIGSESTEDQAMEDIKQMEAESISSSEEIVPNSVEQKHIQKEDVPSERYLGYLEQLLRLKKYKVPQLEIVPNSAEERLHSMKEGIHAQQKEPMIGVNQELAYFYPELFRQFYQLDAYPSGYVPLGTQYTDAPSFSDIPNPIGSENSEKTTMPLW
56
61
63
64
65
66
67
69
73
79
121
123
125
129
30
MKLLILTCLVAVALARPKHPIKHQGLPQEVLNENLLRFFVAPFPEVFGKEKVNELSKDIGSESTEDQAMEDIKQMEAESISSSEEIVPNSVEQKHIQKDDVPSERYLGYLEQLLRLKKYKVPQLEIVPNSAEERLHSMKE GIHAQQKEPMIGVNQELAYFYPELFRQFYQLDAYPSGAWYYVPLGTQYTDAPSFSDIPNPIGSENSGKTTMPLW
a Amidation (-0.98)
c Carbamidomethylation (DHKE, X@N-term) (+57.02)
d Deamidation (NQ) (+0.98)
f Formylation (+27.99)
m Methyl ester (+14.02)
o Oxidation (M) (+15.99)
p Phosphorylation (STY) (+79.97)
p Phosphorylation (HCDR) (+79.97)
s Sodium adduct (+21.98)
s Sulphone (+31.99)
