# Supplementary material for: Post-translational modifications of Drosophila melanogaster HOX protein, Sex combs reduced
Source: PLoS One. 2020 Jan 13;15(1):e0227642. doi: 10.1371/journal.pone.0227642 (PMC6957346; doi:10.1371/journal.pone.0227642)
Supplement: Attachment — Submitted filename: Response to Reviewers.docx [file pone.0227642.s018.docx]

Dear Editor,

We thank Reviewer #1 and Dr. Leslie Pick for their helpful comments on the manuscript, and we are sure that the advice taken will greatly improve the manuscript. The reviewers’ comments are in italics and our responses are in roman.

The page and line numbers mentioned below correspond to the manuscript without track changes with file name ‘Manuscript’.

Reviewer #1

1. The MS however do not experimentally address the functional relevance of any of the PTMs identified.

We interpret the reviewer’s intension here, based on the context of where the comment is made, as part of the summary of our findings and not necessarily criticism that needs to be addressed experimentally as suggested by Dr. Pick. We will address the difficulties in doing functional analysis of SCR when addressing Dr. Pick’s criticism on this topic below.

2. Some Figures with low info content may be combined (Fig.1 to 3).

We thank the reviewer for this suggestion. Figures 1 to 3 from the original manuscript are combined into a single figure (Fig 1).

3. The authors address whether phosphorylation sites preferentially map to SLiM motifs. It is not clear why this specific question is raised, and also why it is addressed only for phosphorylation.

We thank the reviewer for pointing this out. We have clarified our reasons in the introduction (Lines 88-96). We have also included the proportion of SLiMs identified that are annotated as potential phosphosites (64%) in the results (Lines 390-393).

4. Fig. 1 highlights FUNCTIONAL Scr protein domains. The bases for that claim for each motif should be provided. Also it may be useful to complement that figure to show to what extent these domains are conserved over short and/or long evolutionary distances.

We thank the reviewer for this suggestion. We have listed the motifs in the introduction of the revised manuscript with the supporting references to their functional importance lines 53-57. Fig 1A now shows the taxonomic level of conservation of the SCR functional domains/motifs.

5. Fig. 2 shows a single Scr band, raising the issue of whether/how much of these PTMs likely “coexist”. May be a more resolutive gel and less exposed picture would help appreciating the point.

Unfortunately, we do not have a less exposed image for Fig 1B (which was previously Fig 2), and no remaining sample to rerun on a more resolutive gel. The current image is the autoexposed image.

6. Quality of the Figure may be improved for a few panels (in Fig. 3, larvae are apparently not at equivalent stages, and one panel of Fig. 4B is strongly over exposed).

All the larvae are at the first instar larva stage. The brightness and contrast of Fig 1C (previously Fig 3) has been improved. We have also added a lane with a less exposed image (Fig 2C) of the same lane that is overexposed in the present Fig 2B.

7. Discussion, p32, the statement “that the modifications map to functionally important region” is not supported. This point is accurately described in the abstract.

We thank the reviewer for catching this overstatement. We have phrased the conclusion as it is in the abstract (Lines 591 and 592).

8. The authors do not discuss one important limitation that relates the “ectopic” approach. Scr is only expressed very locally in the Drosophila embryo. Following heat shock it is expressed at high levels in all embryonic cells, which may reveal PTMs that do not occur in cells “normally” expressing Scr.

The authors thank the reviewer. This is now addressed in lines 548 and 549.

Reviewer #2 Dr. Leslie Pick

1. The PTMs were analyzed on SCR protein that was expressed throughout the embryo, at high levels and over a very long time period. While this study design is understandable, in that large amounts of protein are needed, it raises the problem that expression outside of the native SCR domain may result in PTMs that do not occur in the cells in which SCR is endogenously expressed. Therefore, the functional significance of the different modifications is unclear. I would like to see at least some functional testing of residues – perhaps at least the phosphorylated residues predicted to be in regions important for function – in order to be able to evaluate the significance of the PTMs identified.

Yes, it would be nice to show functional importance for the phosphorylated residues as Dr. Pick suggests. Two issues make functional analysis difficult in SCR: substoichiometric phosphorylation and differential pleiotropy. Substoichiometric phosphorylation makes it difficult to know in what tissues is SCR phosphorylated if this was the explanation for the substoichiometric levels. If we had found a site in SCR that was stoichiometrically phosphorylated, we may have had more reason to do a functional analysis. Most of the Scr phenotypes described thus far are associated with a limited number of tissues that SCR is expressed in proboscis, salivary glands, male sex combs and embryonic gut; other tissues that express SCR are not well characterized like the nervous system.

Differential pleiotropy also poses a difficulty in analysis of SCR (HOX) function. Differential pleiotropy suggests the HOX proteins outside the HD are composed of many small domains/motifs that make small tissue specific contributions to overall HOX activity. The NANGE motif is one of these motifs, which is surrounded by four phosphosites making it a good candidate for analysis based on position. Although deletion of the NANGE motif has a detectable weak phenotype in suppression of the induction of ectopic proboscises, it is a motif that exhibits differential pleiotropy being strengthened in combination with other mutations in other motifs. Differential pleiotropy makes the analysis of function in HOX proteins very difficult because of the weakness of the phenotypes of single mutants and the genetic interactions with other motifs. In conclusion, the results of a functional genetic analysis of SCR phosphosites may prove inconclusive or difficult to interpret due to the substoichiometric nature of the phosphorylation and differential pleiotropy.

We have added a section to the discussion about these two problems (Lines 579-587).

2. The finding that most of SCR is covered by SLiMs is very surprising to me and I am, quite frankly, skeptical about this result and its potential importance. At a minimum, this needs a lot more explanation. How do you define a SLiM? What are your cut-offs? What are you comparing? Same questions for the conservation. What is compared? How broadly conserved are these? Across drosophilid SCRs? Other species? I would add main text figures on this, and show the sequence alignments with sequences (not just blocks). The Tables in the Supplement are fine but are basically just lists. The main text needs to summarize this information in more than just the schematic shown in the main Figure. I really wonder what the significance of all these is? Would you find this many SLiMs in any protein analyzed? This needs to be tested in order to understand whether this finding is significant.

We thank Dr. Pick for suggesting a figure showing the alignment of the SLiMs identified. The block diagram for SCR SLiMs has been replaced by Fig 3. The SLiMs considered conserved in our analysis are color-coded in red and the numbers beside the SLiMs correspond to the SLiM data in S5 Table which shows the taxonomic level of conservation and putative function. Which SliMs are predicted sites of phosphorylation are also indicated.

The definition of SLiMs is in lines 63-66.

The steps and cut-offs for our SLiM analysis are described in Page 13.

As mentioned in lines 65 and 66, SLiMs have been proposed to be sites of protein-protein interaction, PTMs and cell compartment targeting signals. We have also added statements on the proportion of SCR SLiMs that are putative sites of phosphorylation based on their annotation (Lines 390-393).

3. Line 257: Indicate C-terminal or N-terminal fusion; replace “behind” with “downstream of”

We thank Dr. Pick for this suggestion, and this has been addressed in the revised manuscript. New line numbers are 276 and 277.

4. Line 262: This is an enormous difference. It might be worth stating here which GAL4 was used (even though it's in the methods) and perhaps commenting on whether that is considered a "strong" GAL4 line. Just to point to how generalizable this is - that it is so much stronger (which is something many of us assume but I have not noticed it directly compared like this before to a ubiquitous GAL4 driver)

We thank Dr. Pick for this suggestion. In lines 280 and 281 of the revised manuscript, we have named the driver used armadillo-GAL4, which is thought of as a strong ubiquitous driver.

5. Line 287: It would be helpful if the WT control were imaged in a way that shows the T1 beard. Also, it is now pretty standard to use a UAS-gfp as control rather than a WT animal.

We have replaced (panel III in Fig 1C) with a better cuticle image for WT in order to improve the visibility of the T1 beard. It is difficult to get a clear view of the T1 beard as it is in front of the mouth hooks and head skeleton. For panels I and II for SCR, the larvae have head defects and therefore, the mouth hooks and head skeleton are malformed and do not cover the T1 beard.

6. Line 291: Perhaps worth a mention here or in the methods how you managed the heat shock with 3 grams of embryos - size of tools used to collect, size of mesh - just a few words to point out that this is a larger mass of embryos than your standard collection. Also, please include stage and length of heat shock, here even though it is in the methods

We agree with Dr. Pick, and the size of nylon mesh openings and dimensions of the filter basket used for embryo collection is mentioned in lines 136-138. Lines 298 and 299 have the stage, length and temperature of heat-shock of embryos.

7. Line 300: This is confusing if you are saying that the purified band had less than you need for the MS. You just need more sentences here to explain. It is also notable that a lot of background bands came through the whole purification. Obviously you got the right product in the end but I don't know if you want to comment on that point when discussing Fig. 4. You really cannot see the SCR band above background levels in the Coomassie stained gel.

We agree with Dr. Pick and have added material to this section. We used the known amount of SCRTT purified from bacteria to determine the amount present in our sample purified form Drosophila. The signal strength of the Drosophila expressed protein was compared with the signal of the known amount of bacterially expressed SCRTT protein to determine the concentration of the Drosophila purified sample. We used this information to calculate how much of the Drosophila sample to put on the gel to have 10µg for the MS/MS analysis. Lines 304-312 is rewritten to explain this comparison.

8. Fig. 5: I would make this a supplementary figure. It's kind of distracting to the main story line to see a figure with sequence and then that sequence isn't SCR.

We agree with Dr. Pick and this figure has been moved to the supplemental section and is now S3 Fig.

Additional changes made to manuscript:

1. We noticed that the ELM database which we used to predict SLiMs in SCR protein has been recently updated and therefore, all the results which includes Table 3, Fig 3, S4 and S5 Tables and S10 Fig (ref# 27) have been updated as per the newer version of the database. Please see for updated SLiM analysis in ‘Results’ section on Pages 24 and 25. This resulted in the addition of 2 new SLiMs which are conserved according to our analysis. 2 previous SLiMs have been removed from the database. The multiple sequence alignment was performed again just to make sure that all data reported are up to date. Also, the reference for MAFFT tool has been updated (ref# 66). The accession number of D. pseudoobscura SCR protein has been changed as per NCBI update (S6 Table).
2. Previous supplemental S4 and S5 Tables were removed as they were not cited in the main text. New S4 and S5 Tables contain the updated list of SLiMs used in our analysis. S6 Table now has accession number for D. melanogaster SCR and its animal homologs only. The other HOX accession numbers have been removed from the table.
3. The temperature of heat-shock to induce the expression of SCRTT from the hspScrTT fusion gene was changed to 36.5°C (for preparation of first instar larval cuticles). It was mistakenly mentioned as 37.5°C in the original version of the manuscript (Line 126).
4. Lines 530-542 were added in the ‘Discussion’ section pertaining to SLiM analysis.
5. The reference section has undergone small changes and the in-text citations have been updated accordingly.
