# Supplementary material for: Post-translational modifications of Drosophila melanogaster HOX protein, Sex combs reduced
Source: PLoS One. 2020 Jan 13;15(1):e0227642. doi: 10.1371/journal.pone.0227642 (PMC6957346; doi:10.1371/journal.pone.0227642)
Supplement: Attachment — Submitted filename: Response to Reviewers.docx [file pone.0227642.s019.docx]

Dear Editor,

We thank Reviewer #1 for accepting the revisions to the manuscript and Dr. Leslie Pick (Reviewer #2) for her helpful comments on the manuscript, and we are sure that the advice taken will greatly improve the manuscript. Dr. Pick’s comments are in italics and our responses are in roman.

The page and line numbers mentioned below correspond to the manuscript without track changes with file name ‘Manuscript’.

Reviewer #2 Dr. Leslie Pick

1. The one additional major point I would like to see addressed though is the finding that so much of the SCR protein is comprised of SLiMs.

We have addressed this point in the Discussion section (lines 533-539).

“The ELM database search yielded 81 predicted SLiMs in D. melanogaster SCR that span 63% of the primary sequence of the disordered region of SCR (Table 3). Filtering the predicted SLiMs for evolutionary conservation did reduce the number of SLiMs to 53. As SLiMs have a low information content [20], a database search of SCR sequence may identify false positives, which may explain why a minority of predicted SLiMs were phosphosites. However, this lack of phosphorylation may also suggest that many SLiMs are targets of other PTMs [24-28] like methylation, hydroxylation, carboxylation, acetylation and formylation.”

2. First, the authors should make clear in the text and figure that “conserved” in this case refers to Drosophila species. This is quite different from an earlier figure where conserved included all arthropods - hundreds of millions of years of difference in divergence.

SCR SLiMs predicted by the ELM database (S4 Table) were manually checked for conservation across species which included protostomes and deuterostomes (lines 263-271). 39 out of 53 “conserved” SLiMs are conserved among Drosophilids. However, 14 “conserved” SLiMs are conserved beyond Drosophila like VDYTQLQ (SLiM #31) which is conserved across Diptera, YTQL (SLiM #33) which is conserved across Insecta, SSYQF (SLiM #4) which is conserved across Arthropoda, YPWM (SLiM# 74) which is conserved across Bilateria, etc. The extent of conservation of “conserved” SLiMs is documented in S5 Table.

3. Second, I ask that the authors experimentally (bioinformatically) address the question I raised previously: Would you find this many SLiMs in any protein analyzed? This needs to be tested in order to understand whether this finding is significant, especially since experimental testing of individual sequences is not realistic in this system.

Yes, the low information content of SLiMs does lead to a large number of predicted SLiMs in any protein. We randomly chose 8 D. melanogaster nuclear proteins and submitted their amino acid sequences to the ELM database to find predicted SLiMs in those proteins. 9-75% of the total protein was SLiMs with an average of 42%. For HOX proteins in D. melanogaster, 46-64% were SLiM sequences with an average of 57% (refer to tables below).

| **D. melanogaster nuclear protein** | **UniProt ID** | **Protein size (aa)** | **Predicted SLiM size (aa)** | **% SLiMs** | **Mean % SLiMs** |
| --- | --- | --- | --- | --- | --- |
| Cyclin-dependent kinase 8 | Q9VT57 | 454 | 42 | 9 | 42 ± 21.9 |
| Helicase | Q9VRI0 | 560 | 185 | 33 |  |
| Nucleoporin seh1 | Q7K2X8 | 354 | 98 | 28 |  |
| AT07420p | Q7JWU9 | 249 | 108 | 43 |  |
| RNA-binding protein 8A | Q9V535 | 165 | 86 | 52 |  |
| Histone RNA hairpin-binding protein | Q9VAN6 | 276 | 206 | 75 |  |
| Cyclin-C | P25008 | 267 | 65 | 24 |  |
| Histone chaperone asf1 | Q9V464 | 218 | 160 | 73 |  |

| **D. melanogaster HOX protein** | **Protein (disordered) size (aa)** | **SLiM**  **size (aa)** | **% SLiM** | **Mean % SLiMs** |
| --- | --- | --- | --- | --- |
| LAB | 576 | 366 | 64 | 57 ± 5.5 |
| PB | 724 | 461 | 64 |  |
| DFD | 533 | 287 | 54 |  |
| SCR | 364 | 228 | 63 |  |
| ANTP | 325 | 175 | 54 |  |
| UBX | 336 | 156 | 46 |  |
| ABD-A | 277 | 156 | 56 |  |
| ABD-B(m) | 440 | 251 | 57 |  |
| ABD-B(r) | 217 | 125 | 58 |  |

However, bioinformatic analysis has shown that human disease mutations in intrinsically disordered regions do map frequently to validated and predicted SLiMs indicating that SLiMs are functional. The function of SLiMs has been tested in many proteins both viral and host. We have included this evidence in the introduction when discussing SLiMs (lines 63-76).

“SLiMs or Eukaryotic Linear Motifs (ELMs) are short stretches of protein sequence (typically 3-10 amino acids long containing 2-3 specificity-determining residues) present in regions of intrinsic disorder [20-23]. SLiMs function as sites of protein-protein interaction, PTMs and cell compartment targeting signals [20-28]. Experimentally validated SLiMs have been curated into the ELM database and the database is used to predict SLiMs [28]. Although SLiMs have a low information content and are predicted to occur frequently in the proteome, the 22% of human disease mutations that occur in intrinsically disordered regions of human proteins map frequently to functionally important residues of validated SLiMs and also map frequently to predicted SLiMs suggesting that predicted SLiM sequences are correlated with function [29]. Viral proteins have SLiMs that mimic the SLiMs in host proteins which enables the virus to evade host immune functions [30,31]. Phosphorylation of the PDZ-binding motif (PBM), which is a SLiM, in the disordered C-terminal tail of ribosomal S6 kinase 1 (RSK1) protein is important for epidermal growth factor regulation [32]. The annotation of SLiMs in the ELM database suggest that a significant proportion are targets of phosphorylation.”

4. Are there examples of proteins analyzed in this way for which the function of the predicted SLiMs has been experimentally tested or verified?

We give one of many examples in the introduction. See above. Also, the ELM database is a curated collection of validated SLiMs which is searched to find predicted SLiMs. We also looked for conservation of the SLiMs as an additional criterium for the potential of function.

5. Basically, the conclusion now is based on analysis in one database. I personally am not familiar with the stringency of this database but I am still very skeptical about the finding. I would be happy to be convinced of its validity – it would help our own research enormously – but I need more evidence.

We understand Leslie Pick’s concern, which is why we assessed whether predicted SLiMs were preferential sites of phosphorylation and they are not, suggesting some skepticism about the use of the SLiM database to predict phosphosites beyond just having the three amino acids that are phosphorylated. However, as we now point out in the manuscript, human disease mutations map frequently to SLiMs predicted from the ELM database, suggesting that this database has a use in identifying potential functional protein elements. Short functional sequences are difficult to predict and work with and tools like the ELM database need to be developed and tested for their usefulness.

6. Most of the other points were addressed adequately although I did not some unusual grammar and punctuation in some of the changes – it needs a quick proofreading before resubmission.

The revised manuscript has been thoroughly checked for grammar and punctuation.
